# Simplified Preparation of ppm Pd-Containing Nanoparticles as Catalysts for Chemistry in Water

**DOI:** 10.1021/acscatal.3c00007

**Published:** 2023-02-17

**Authors:** Yuting Hu, Xiaohan Li, Gongzhen Jin, Bruce H. Lipshutz

**Affiliations:** Department of Chemistry & Biochemistry, University of California, Santa Barbara, California 93106, United States

**Keywords:** nanoparticles, cross-couplings, micellar catalysis, chemistry in water, Pd catalysis

## Abstract

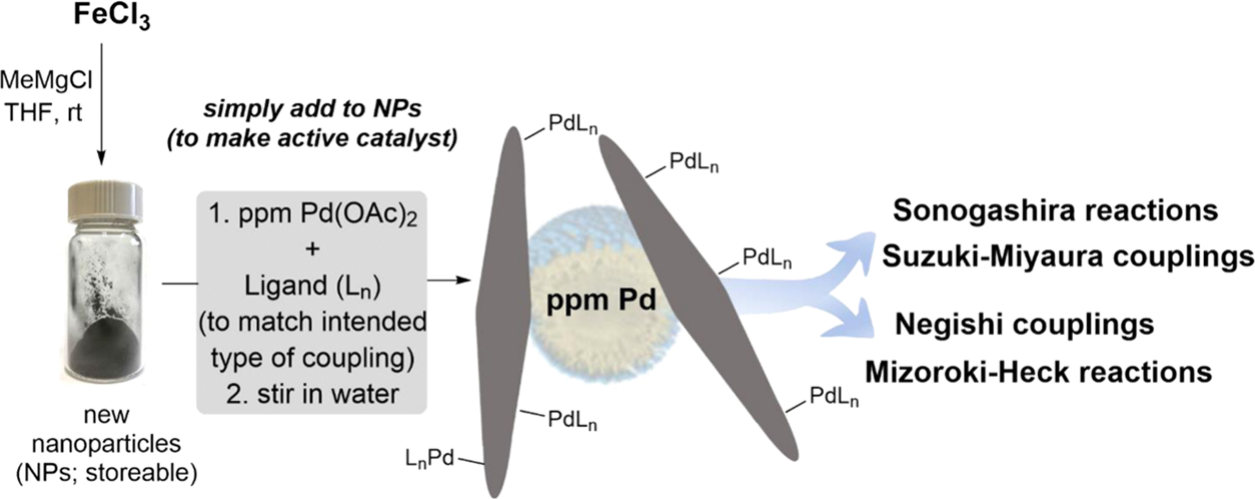

A protocol has been developed that not only simplifies the preparation of nanoparticles (NPs) containing ppm levels of ligated palladium that affect heterogeneous catalysis but also ensures that they afford products of cross-couplings reproducibly due to the freshly prepared nature of each reagent. Four different types of couplings are studied: Suzuki–Miyaura, Sonogashira, Mizoroki–Heck, and Negishi reactions, all performed under mild aqueous micellar conditions. The simplified process relies on the initial formation of stable, storable Pd- and ligand-free NPs, to which is then added the appropriate amount of Pd(OAc)_2_ and ligand-matched to the desired type of coupling, in water.

## Introduction

Back in 2015, a report from these laboratories disclosed a relatively simple preparation of Fe/ppm Pd spherical nanoparticles (NPs) from FeCl_3_ and MeMgCl mixed in tetrahydrofuran (THF) with a controlled amount of Pd(OAc)_2_ and ligand SPhos.^1^ The resulting powdery mustard-colored material could then be used as what is now known to be a precatalyst,^2^ whereupon addition of an aqueous solution containing the designer surfactant TPGS-750-M^3^ (only 2 wt %) led to a reconfigured, rod-shaped catalyst. This resulting heterogeneous reaction mixture is very effective at mediating complex Suzuki–Miyaura couplings in water, where only 320 ppm palladium (i.e., 0.032 mol %) is enough to form sp^2^–sp^2^ bonds under mild conditions, all in water.

**Scheme 1:**
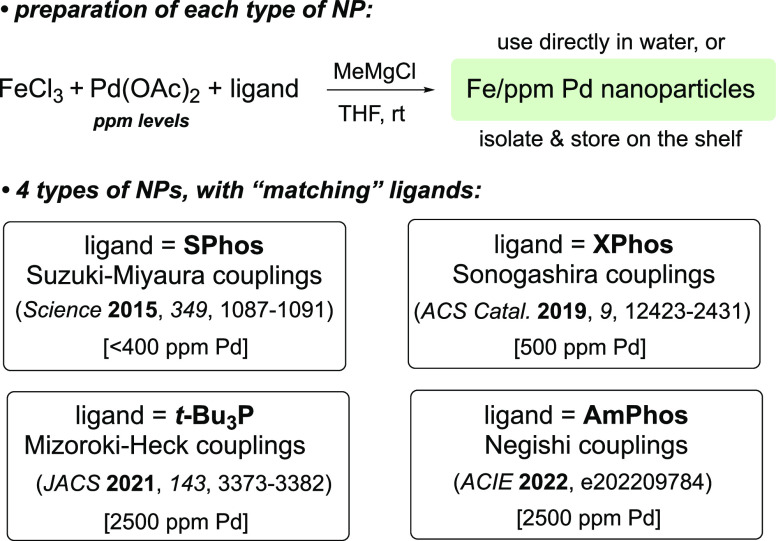
Preparation of Four Individual Types of Nanoparticles for Various Pd-Catalyzed Couplings

Since this initial report,^1^ we have shown that by simply altering the amount of Pd and the corresponding ligand added to the FeCl_3_ in THF, followed by addition of a Grignard reagent, NPs can be fashioned such that C–C bonds can be realized characteristic of Sonogashira,^4^ Mizoroki–Heck,^2^ and, most recently, Negishi couplings.^5,6^ Each reaction type is also run at ppm levels of Pd embedded within each catalyst (i.e., Sonogashira: 500 ppm or 0.05 mol %; Mizoroki–Heck: 1000–2500 ppm or 0.10–0.25 mol %; and Negishi: 2500 ppm or 0.25 mol %; Scheme 1). These NPs were engineered to be both synthetically effective and environmentally responsible, made all the more so given the opportunities for recycling of the aqueous reaction mixtures as well as the seemingly unlimited tandem one-pot sequences involving multiple reaction types, including biocatalytic processes.^7^ Notwithstanding these virtues that include the important observation that low levels of residual metal are to be anticipated in the products, the perspective, especially from industry, remains that NPs on the market are expensive and affected by batch-to-batch variability, thus limiting their widespread use.

This viewpoint is quite reasonable since their storage “on the shelf” (i.e., their handling) can be variable. Moreover, the presence of a phosphine ligand in each is enough to trigger concerns about eventual reactivity given their penchant toward autoxidation. Hence, while in all cases one has the option of preparing NPs and then immediately using them *in situ*, the industry (and the field, in general) is rightly concerned about reproducibility. To fully address these issues, we have developed an alternative protocol, applicable to all four NPs that catalyze C–C bond constructions that eliminates these important potential limitations; this is the subject of the report herein.

## Results and Discussion

**Scheme 2:**
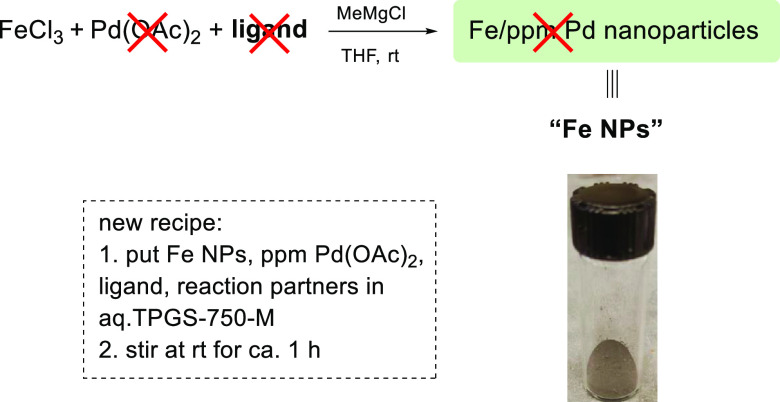
New Procedure Leading to “Generic” Fe NPs: No Pd, No Ligand

The revised protocol involves preparing NPs via treatment of FeCl_3_ with the same Grignard reagent in THF, but in the complete absence of both the Pd(OAc)_2_ and ligand (Scheme 2). This leads to the precipitation of NPs that can either be used directly or, more to the point, be collected (after removal of the solvent *in vacuo*) and stored over time.^8^ Optimization studies (see the Supporting Information, SI) led to this new preparation of NPs for each reaction type.

Regardless of the particular cross-coupling of interest (*vide infra*), these generic NPs are then added to an aqueous micellar medium containing 2 wt % TPGS-750-M (Table 1) at rt, to which is then introduced Pd(OAc)_2_ and the appropriate ligand. By allowing this aqueous mixture to stir at rt under an inert atmosphere, the initially formed NPs undergo “sculpting” (from ca. 5 nm spheres to >100 nm rods) along with equilibration (of the ligand, distributing between the NPs and nanomicelles in the water), arriving at the active catalyst within the reaction mixture.^2^ At this point, the coupling partners need only be added and the mixture stirred vigorously. Control experiments between heteroaryl bromide **1** and boronic acid **2** leading to biaryl product **3** document the essential roles played by all of the ingredients: the Pd, the ligand, and the newly fashioned NPs.^9^

**Table 1:**
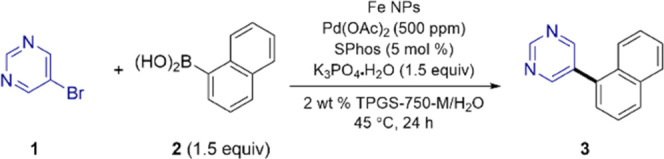
Control Experiments for NP-Catalyzed Suzuki–Miyaura Couplings

entry^a^	variation from above	yield
1	none	98^b^
2	without Fe NPs	<5%^c^
3	without Pd(OAc)_2_	<5%^c^
4	without SPhos	<5%^c^

a 5-Bromopyrimidine (0.2 mmol), naphthalen-1-ylboronic acid (0.3 mmol), Fe NPs (8 mg, 5% Fe NPs), Pd(OAc)_2_ (500 ppm) in pot, K_3_PO_4_·H_2_O (0.3 mmol), 2 wt % TPGS-750-M/H_2_O (0.4 mL), 45 °C, 24 h.

b Isolated yield.

c Yield by ^1^H NMR, ethylene carbonate as the internal standard.
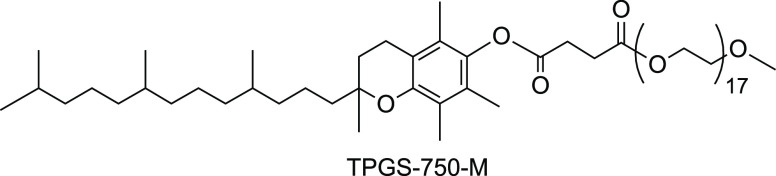

Using this novel procedure for NP catalyst formation, each of the four types of Pd-catalyzed processes was tested for generality, using the same initially prepared NPs. Hence, as illustrated in Table 2, several reactions involving Suzuki–Miyaura, Sonogashira, Mizoroki–Heck, and Negishi couplings were studied. Both aromatic and/or heteroaromatic reaction partners readily participate, leading to products in good-to-high yields, including late-stage functionalization that reveals the effectiveness of this approach. All cases involve sustainable catalyst loadings of only 500–2500 ppm (0.05–0.25 mol %) Pd, while each avoids reaction in waste-generating and environmentally impactful organic solvents.^10^ Instead, water is the main, or exclusive, reaction medium leading to an overall combination used under very mild conditions.^11^ From the perspective of TON, use of only 500 ppm (0.05 mol %) of Pd translates into a value of 2000.

**Table 2:**
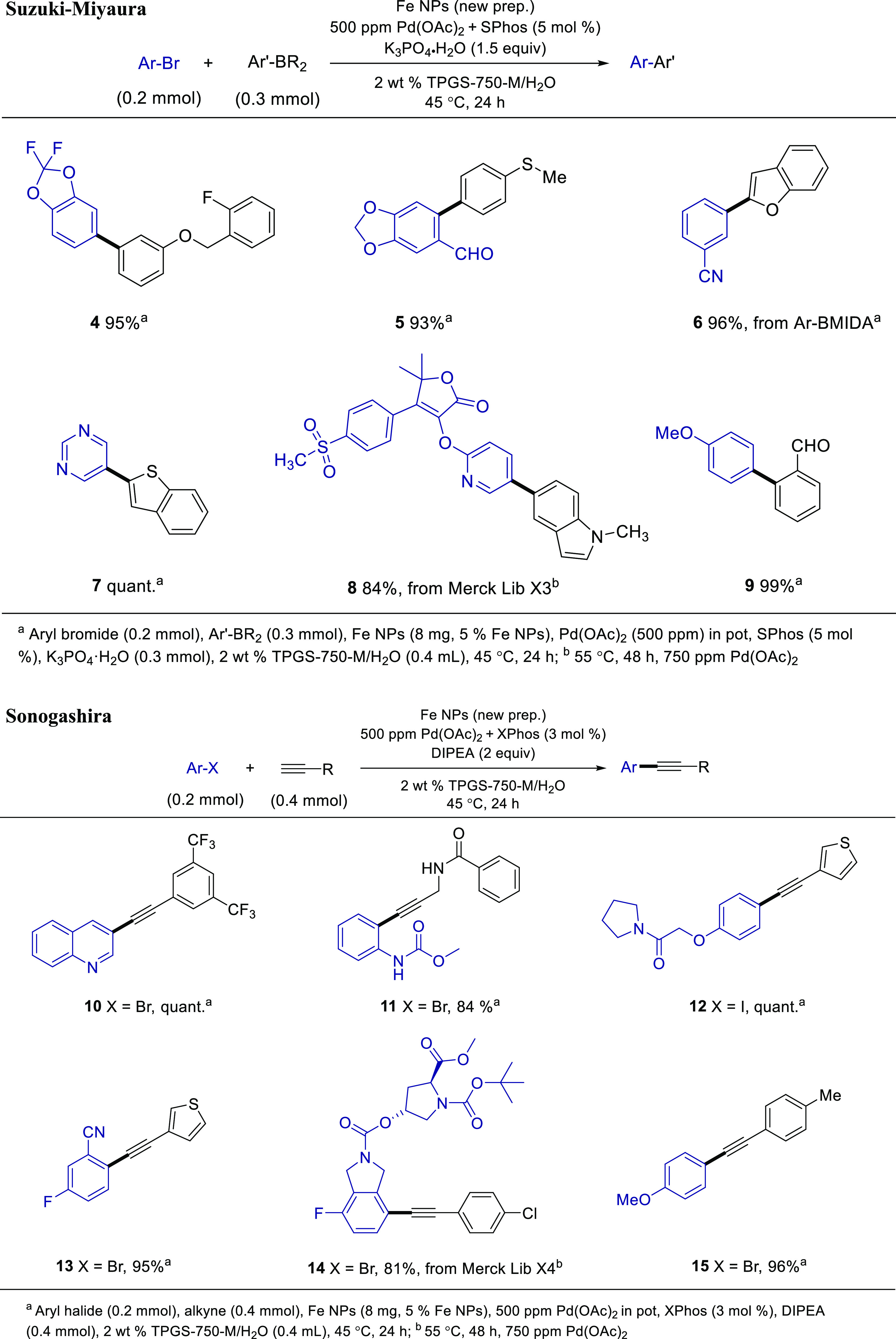

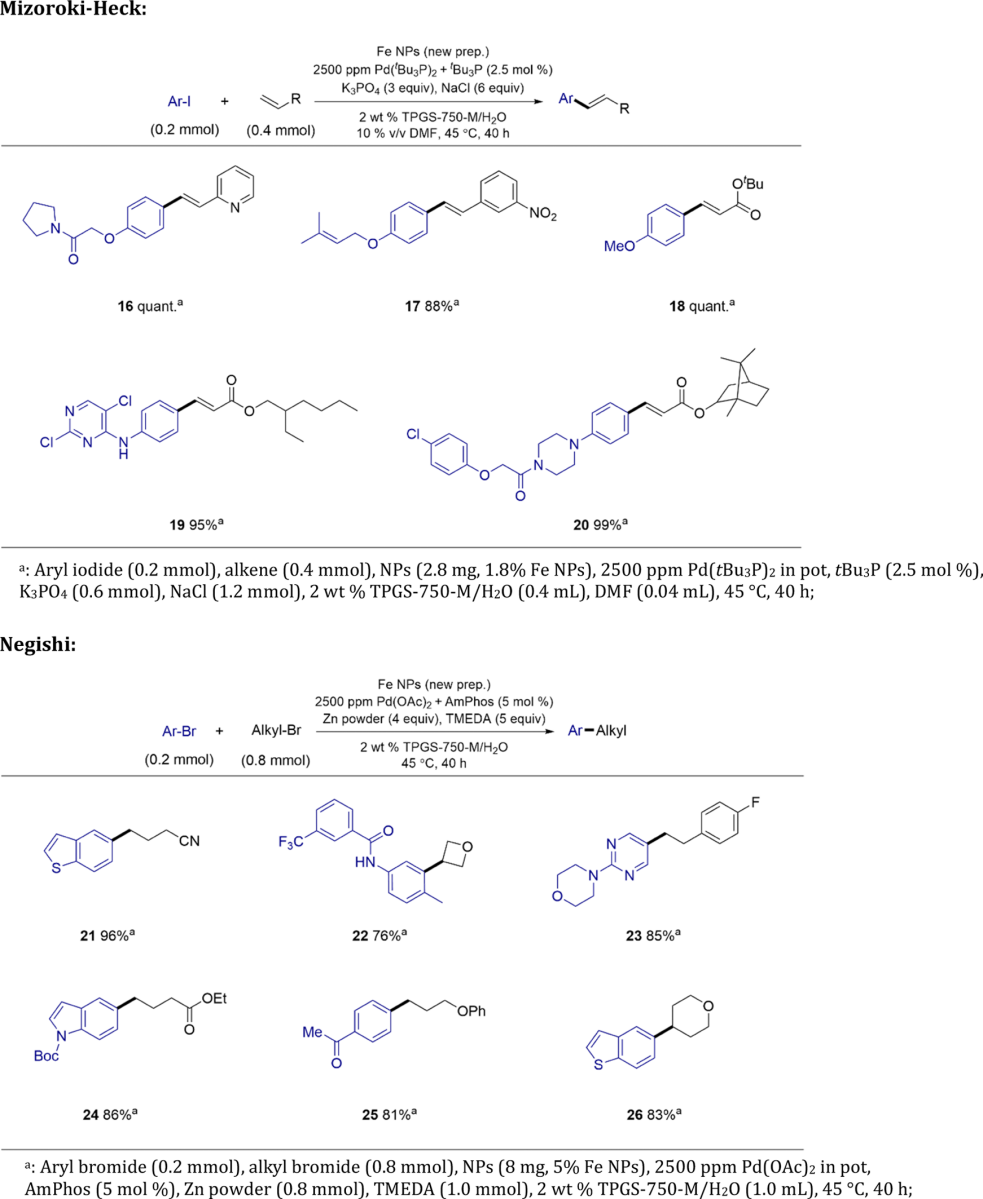
Representative Cases of Coupling Reactions Using the New Preparation of NPs

**Figure 1:**
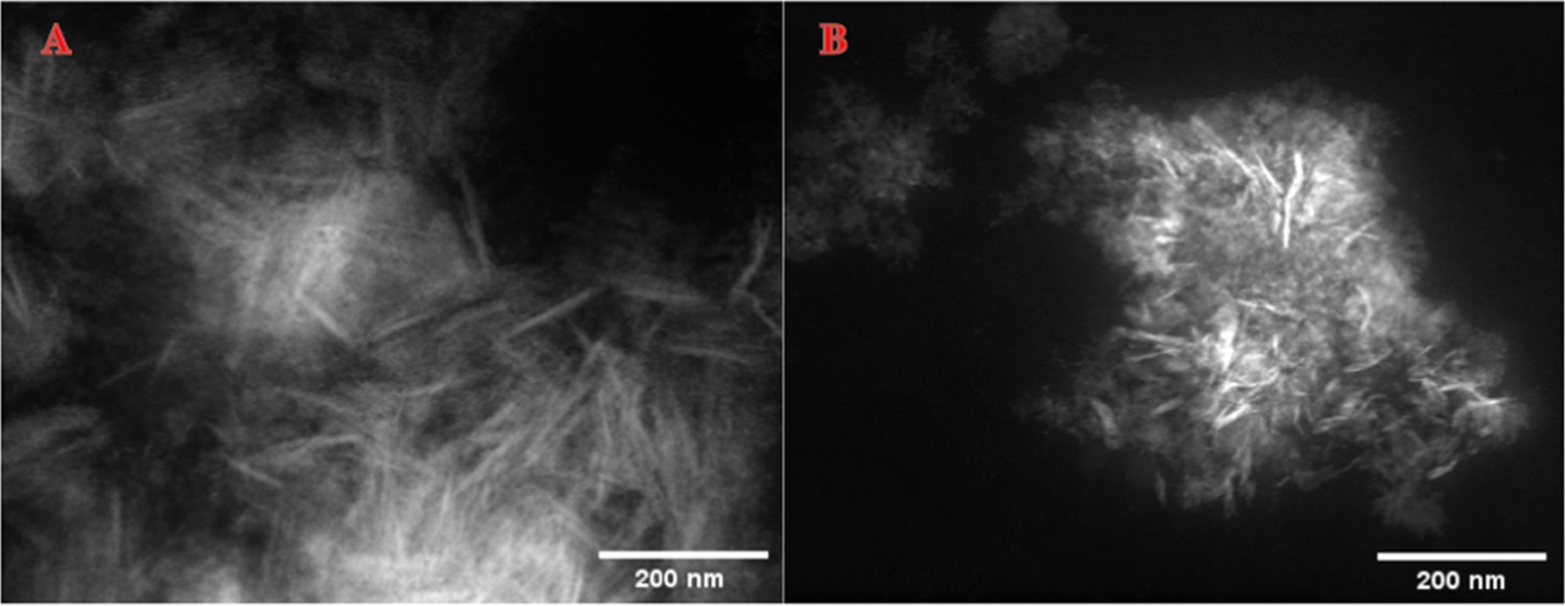
STEM image for the NPs formed via the new route vs those following the original recipe. (A) High-angle annular dark field-scanning transmission electron microscopy (HAADF-STEM) image of Fe NPs with Pd(OAc)_2_ and SPhos in 2 wt % TPGS-750-M/H_2_O following the new approach. (B) Fe/ppm Pd nanocatalyst with SPhos in 2 wt % TPGS-750-M/H_2_O following the original procedure.

Scanning transmission electron microscopy-energy-dispersive X-ray spectroscopy (STEM-EDS) analysis was carried out to further investigate the nature of these NPs. Comparisons between the NPs formed following the original recipe^1^ and those prepared via the new procedure have been made. Hence, after stirring with Pd(OAc)_2_ and SPhos in 2 wt % TPGS-750-M/H_2_O solution, the new Fe NPs (Figure 1A) show the same shape (i.e., nanorods) and size (ca. 100 nm) as those formed using the original protocol (Figure 1B). Moreover, the elementary compositions for the NPs prepared via this alternative route and those resulting from the original procedure both show Fe, P, and Cl (see the SI), suggesting that the same catalytic mixture is being formed using either protocol. This is an especially important finding since the combination of the nanomicelles (containing the reaction partners) together with the NPs (serving as catalysts) leads to the previously established “nano-to-nano” effect.^12^ That is, the MPEG portion of the amphiphile serves as ligands for the palladium on the surface of the NPs, in effect “delivering” the reaction partners to the catalyst. This explains the mild conditions involved in this heterogeneous catalysis, a phenomenon that can only exist in aqueous micellar media.

By selecting one case from each of these four types of couplings that affords products involving newly fashioned sp^2^–sp^2^ or sp^2^–sp^3^ bonds, direct comparisons with reactions run with previously formed NPs have also been carried out. The results shown in Table 3 confirm that in all cases better couplings (within experimental error) are to be expected using the newly prepared NPs made as described herein. This improvement is attributed to the “freshness” of the *in situ-*formed NPs, where palladium is positioned onto the surface of the NPs just prior to use. Moreover, minimization of oxidation of the phosphine ligand is likely a major factor since it plays a crucial role in each coupling (e.g., see Table 1).

**Table 3:**
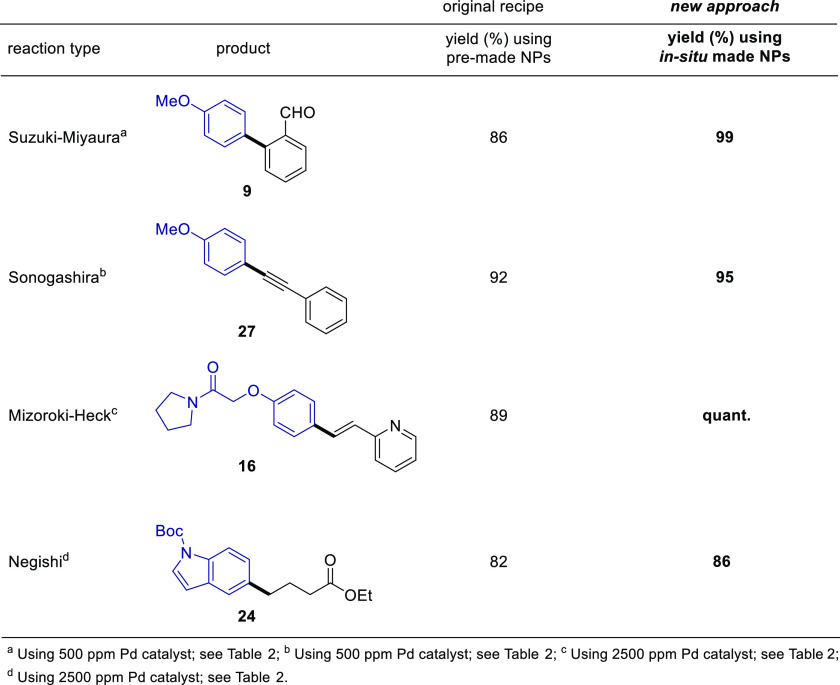
Comparisons between Premade and *In Situ*-Prepared NPs as Catalysts for Coupling Reactions

**Scheme 3:**
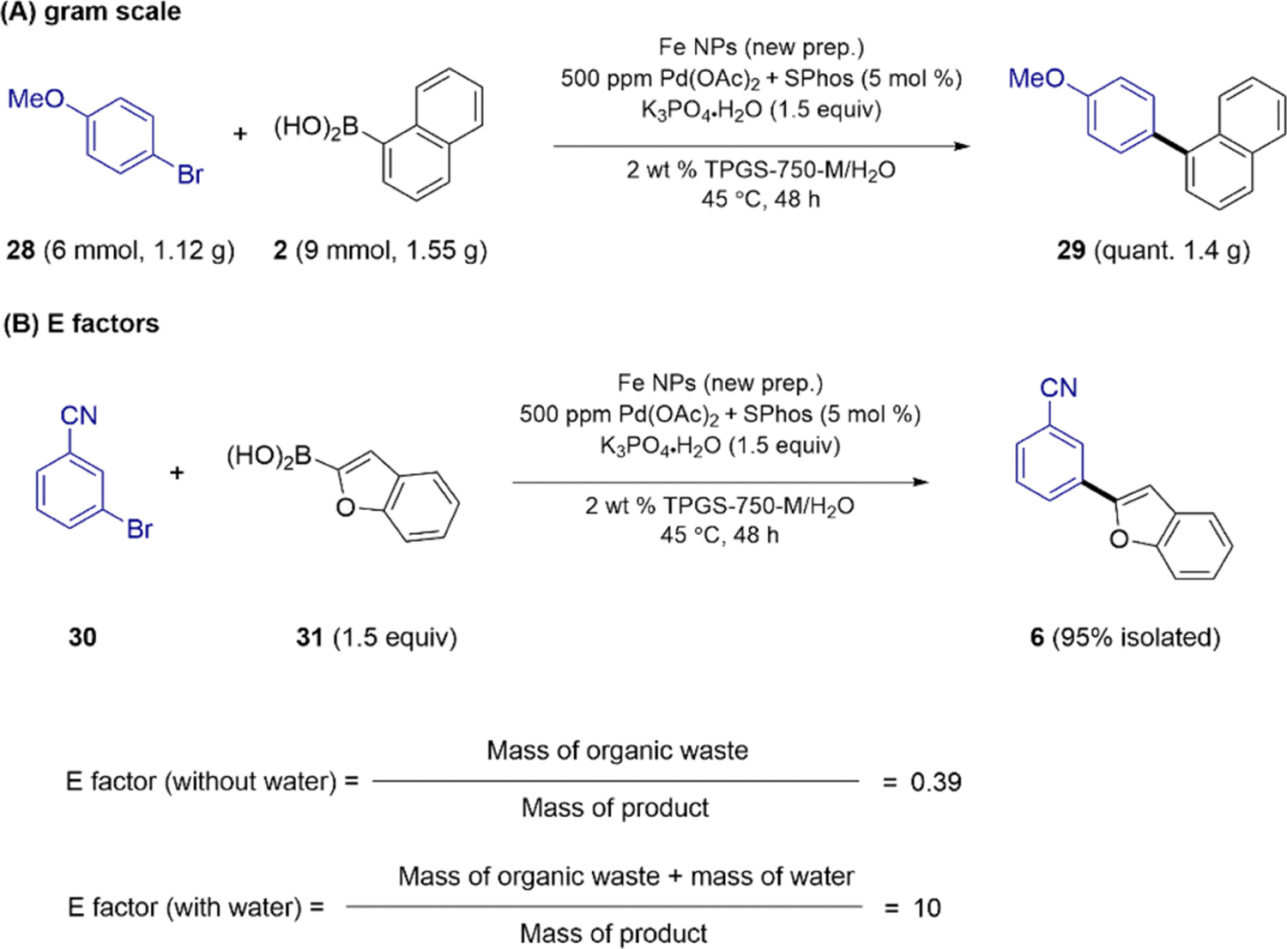
(A) Representative Gram-Scale Coupling; (B) E-Factor Calculations

A gram-scale reaction was carried out, as illustrated in Scheme 3 (A) between **28** and 1.5 equiv of **2**, leading to 1.4 grams of biaryl **29**, formed in quantitative yield. The E factors^13^ (B) associated with these reactions, determined both with and without the water as part of the calculation from the reaction between aryl bromide **30** and boronic acid **31** leading to biaryl **6**, were only 0.39 and 10, respectively, suggestive of the greenness associated with this process.^14^

Another major benefit associated with this new approach to ppm Pd NP catalysis is the availability to change the ligand without making new NPs. Some ligands may have certain limitations, such as air sensitivity or impart poor reactivity to the resulting NPs for specific substrates. As shown in Table 4, the facility with which ligands can be varied offered the opportunity to evaluate alternative, air-stable ferrocene-based ligands recently introduced by Colacot and co-workers,^15^ and are now items of commerce from Sigma-Aldrich.^16^ These completely avoid the issues noted above. Using ligand **L1** as an alternative to SPhos provided far better results for the two Suzuki–Miyaura reactions tested, leading to biaryls **32** and **33**. Likewise, using ligand **L2** instead of Fu’s ligand removes the air-sensitivity issue associated with *t*-Bu_3_P^17^ while significantly improving the NP catalyst reactivity, affording enhanced isolated yields of unsaturated amide **34** and enoate **35**. Other ligands tested, in particular QPhos, which has recently become available in quantity,^18^ were not competitive in any of these four reaction types.

**Table 4:**
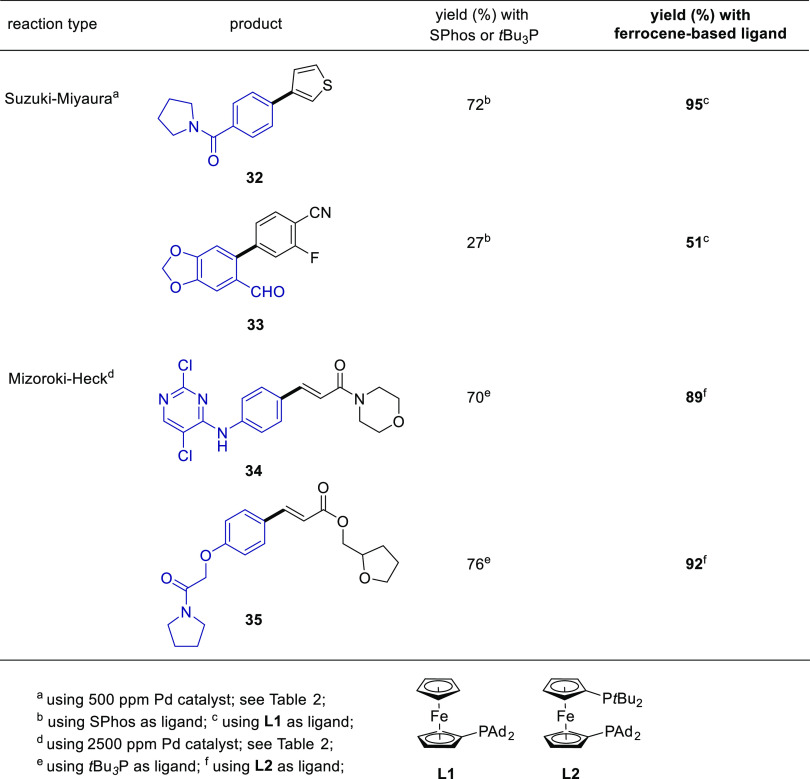
Comparisons between Ligands Used in Suzuki–Miyaura and Mizoroki–Heck Couplings

**Scheme 4:**
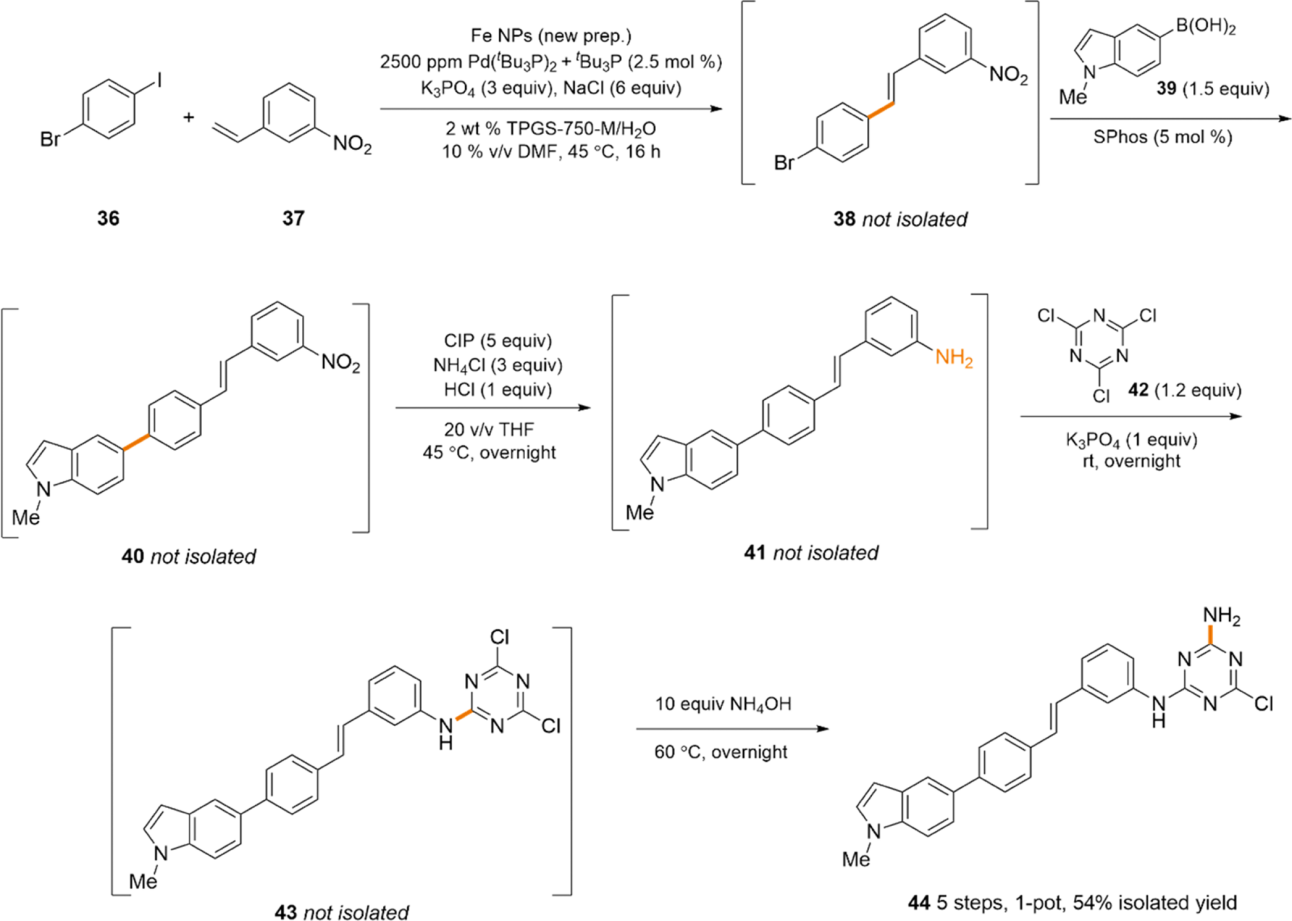
Sequential Reactions, Including Two ppm Pd NP-Catalyzed Couplings, All in Water

While the toolbox now available for running reactions in water has grown,^19^ so has the number of processes in the biocatalysis area that, likewise, benefit just from the presence of a surfactant in the aqueous medium.^21^ Independent of the use of micellar catalysis applied to chemocatalysis, simply having, e.g., TPGS-750-M (2 wt %, or 20 mg/mL) present can dramatically enhance both the rate of an enzymatic step and the extent of conversion, thereby enabling chemoenzymatic catalysis that is “green” in each component (i.e., both the chemo- and biocatalysis steps). This phenomenon has now been documented for reactions involving KREDs,^20^ EREDs,^21^ ATAs,^22^ IREDs,^23^ and ester-forming lipases,^24^ all in water. As illustrated in Scheme 4, the field has also advanced to include tandem processes that go well beyond the more commonly used chemocatalysis/biocatalysis (i.e., two steps in either order).^7^ In the sequence shown, five steps are carried out, all in one pot, and all in water. An initial Mizoroki–Heck coupling between aryl dihalide **36** and styrene **37** takes place exclusively at the iodide, giving chalcone **38**. A Suzuki–Miyaura coupling then occurs on the aryl bromide portion of **38** upon addition of an indoleboronic acid **39**, together with SPhos, leading to the coupled product **40**. It is especially worthy of note here that only the ligand associated with mediating these couplings (i.e., SPhos) was added; *no additional Pd was needed*, as the originally used NPs for the Mizoroki–Heck coupling could be reused by providing the ligand and simply allowing for the re-equilibration, in the aqueous medium, of the *in situ*-derived catalyst needed. Thus, with proper sequencing of reactions, the palladium present already at the ppm level in water can be used further to great advantage, leading to “metal economy.” Without isolation, reduction of the aryl nitro group in **40** using carbonyl iron powder (CIP)^25^ leads to the corresponding aniline **41**, which then readily participates in a mono-S_N_Ar addition^26^ to cyanuric chloride (**42**) to form the intermediate **43**. Lastly, addition of excess NH_4_OH provides the ammonia that then undergoes a second S_N_Ar addition to **43**, ultimately arriving at polycyclic **44**, representing a five-step, one-pot sequence, with the final product being isolated in 54% overall yield. To the best of our knowledge, no other NP catalyst can be used in such a fashion (in the same reaction vessel, in the same medium, and with metal economy).

## Conclusions

In summary, a new protocol for preparing nanoparticles (NPs) has been developed that enables their use for several Pd-catalyzed coupling reactions performed in water at the ppm level of precious metal. This is an especially important advance in that this protocol:

(1) generalizes and standardizes the route to NPs that can be tailored to the intended coupling without concerns over reproducibility;
(2) minimizes opportunities for both the palladium and the associated phosphine to undergo unwanted oxidation, clearly resulting in improved overall reaction efficiencies;
(3) allows for use of the Pd present in the aqueous reaction mixture, in ppm amounts, to be reused for other types of couplings, thereby maximizing “palladium economy;” and
(4) suggests that newly fashioned ligands may be even better matched to these newly prepared and standardized NPs than those previously used, further enhancing and broadening their effectiveness as catalysts.

Based on this new preparation of ligated NP precursors to catalysts that function very effectively under aqueous micellar catalysis conditions, several additional transition metals (e.g., Rh, Ir, etc.) can easily replace Pd, thereby forming new NPs for evaluation at the ppm level of usage in a variety of reactions of synthetic value. Such studies are now underway in these laboratories, the results from which will be reported in due course.

## Supporting Information Available

Procedures for optimizations and couplings, analyses, recycling, and E Factor determination (PDF)
