# Supplementary material for: Simplified Preparation of ppm Pd-Containing Nanoparticles as Catalysts for Chemistry in Water
Source: ACS Catal. 2023 Feb 17;13(5):3179–86. doi: 10.1021/acscatal.3c00007 (PMC9990150; doi:10.1021/acscatal.3c00007)
Supplement: Supplementary file 1 — cs3c00007_si_001.pdf [file cs3c00007_si_001.pdf]

Supporting Information

Simplified Preparation of *ppm Pd*-Containing Nanoparticles as Catalysts for Chemistry in Water

Yuting Hu,† Xiaohan Li,† Gongzhen Jin,† and Bruce. H. Lipshutz*†

† Department of Chemistry and Biochemistry, University of California, Santa Barbara, California 93106, USA

*lipshutz@chem.ucsb.edu

Table of Contents

General Information S3

Optimization S4

Optimization of preparation of different NPs S4

Optimization on reaction conditions for Suzuki reaction S6

Optimization on reaction conditions for Sonogashira reaction S7

Optimization on ferrrocene-based ligand on four different reactions S7

Titration of MeMgCl in THF solution with LiCl/I_2 .... S9

General Procedure for Suzuki coupling reactions S9

General Procedure for Sonogashira coupling reactions S10

General Procedure for Mizoroki-Heck coupling reactions S10

General Procedure for Negishi coupling reactions S11

Analysis for nanoparticles S12

STEM images for new Fe NPs with SPhos S12

Energy-dispersive X-ray Spectroscopy (EDS) analysis for new Fe NPs with SPhos S13

STEM images for Fe/ppm Pd NPs with SPhos from original recipe S14

EDS analysis for Fe/ppm Pd NPs with SPhos from original recipe S15

E Factor, residual metal, and recycling studies S16

E Factor S16

Gram scale reaction S17

Recycling reactions S18

1-Pot sequence of reactions S21

Analytical data for products S23

References S44

^{1}H NMR, ^{13}C NMR and ^{19}F NMR spectra S45

General Information

All commercial reagents were used without further purification unless otherwise noted. The THF used for the preparation of NPs was distilled using a sodium benzophenone ketyl system. All other solvents were used as received, such as MeOH, EtOAc, hexanes, and Et_2O, unless otherwise noted, and purchased from Fisher Scientific. $FeCl_3$ (anhydrous, 98%) was purchased from Alfa Aesar (Lot number: D06Q37) and stored in an argon purged glove box. Methylmagnesium chloride was purchased from Sigma-Aldrich (product number: 189901) and was titrated precisely. Ligands were either purchased from Sigma-Aldrich, Combi-Block or received from Johnson Matthey. Palladium acetate was received from Johnson Matthey. $Pd(^tBu_3P)_2$ was purchased from Sigma-Aldrich. All palladium catalysts and ligands were stored in an argon purged glove box. K_3PO_4•H_2O was purchased from Sigma-Aldrich (⩾95%, product number: 04249). DIPEA was purchased from Sigma-Aldrich (ReagentPlus®, ⩾99%, product number: D125806). K_3PO_4 was purchased from Fisher Chemical (FCC, 97%, catalog number: 18-605-684). NaCl was purchased from Fisher Chemical (Crystalline/Certified ACS, catalog number: S271-500). Zinc powder was purchased from Alfa Aesar (~100 mesh, 99.9%, Lot number: Z21B027) and stored in an argon purged glove box. TMEDA was purchased from Sigma-Aldrich (⩾99.5%, purified by redistillation, product number: 411019). A solution of 2 wt % TPGS-750-M/H_2O solution was prepared by dissolving TPGS-750-M in degassed HPLC grade water and was stored in Schlenk flask under argon. TPGS-750-M was made as previously described[1] and is available from Sigma-Aldrich (catalog number 733857). A standard 2 wt % aqueous solution of TPGS-750-M was typically prepared on a 100 g scale by dissolving 2 g of the TPGS-750-M wax into 98 g of thoroughly degassed[1] (steady stream of argon, minimum of 12 h bubbling time with stirring and heating) HPLC grade water in a Schlenk flask equipped with a stir bar, and allowed to dissolve overnight with vigorous stirring under argon pressure (NOTE: Do not attempt to degas the aqueous phase with surfactant present; vigorous foaming will occur). The 2 wt % TPGS-750-M/H_2O solution, once prepared, was kept in a Schlenk flask. Thin layer chromatography (TLC) was done using Silica Gel 60 F254 plates (0.25 mm thick) purchased from Merck. Column chromatography was done in glass columns using Silica gel 60 (EMD, 40-63 μm) or with pre-packed 25-gram KP-Sil Biotage$^{\Theta}$ SNAP Cartridges on the Biotage$^{\Theta}$ Isolera

One autocolumn. GC-MS data was recorded on an Agilent Technologies 7890A GC system coupled with Agilent Technologies 5975C mass spectrometer using HP-5MS column (30 m × 0.250 mm, 0.25 μ) purchased from Agilent Technologies. ^{1}H, ^{13}C, and ^{19}F NMR spectra were recorded at 25 °C on an Agilent Technologies 400 MHz, a Varian Unity Inova 500 MHz, Varian Unity Inova 600 MHz, Bruker Avance III HD 400 MHz or Bruker Avance NEO 500 MHz spectrometer in $CDCl_3$ with residual $CHCl_3$ (^{1}H = 7.26 ppm, ^{13}C = 77.16 ppm) or in DMSO-d_6 with residual $(CH_3)_2SO$ (^{1}H = 2.50 ppm, ^{13}C = 39.52 ppm) as internal standards. Chemical shifts are reported in parts per million (ppm). NMR Data are reported as follows: chemical shift, multiplicity (s = singlet, d = doublet, dd = doublet of doublets, ddd = doublet of doublet of doublets, t = triplet, td = triplet of doublets, q = quartet, quin = quintet, m = multiplet), coupling constant (if applicable), and integration. Chemical shifts in 13C NMR spectra are reported in ppm on the δ scale from the central peak of residual $CDCl_3$ (77.16 ppm) or the central peak of DMSO-d_6 (39.52 ppm). High-resolution mass analyses (HRMS) were recorded on Waters GCT Premier GC TOF or Agilent 6230 TOF LC/MS System. STEM images were obtained using ThermoFisher Talos G2 200X TEM/STEM w/ChemiSTEM EDS.

Optimization

Optimization of the preparation of different NPs

General procedure for preparation of only Fe NPs-1:

In an oven dried 25 mL microwave reaction vial purged with argon, covered with a rubber septum containing a PTFE-coated magnetic stir bar, 250 mg $FeCl_3$ (1.54 mmol) and 4 mL dry THF were added under a stream of dry argon. The mixture was stirred for 15 min. While maintaining a dry atmosphere at rt, 3.1 mL of 1 M solution of MeMgCl in THF was very slowly (1 drop/2 sec) added to the reaction mixture. After complete addition of the Grignard reagent, the mixture was stirred for an additional 15 min at rt. THF were removed by rotary evaporator, and the solid-state Fe NPs-1 were transferred and stored in glove box.

General procedure for preparation of Fe/ppm Pd NPs-2

In an oven dried 25 mL microwave reaction vial purged with argon, covered with a rubber septum containing a PTFE-coated magnetic stir bar, 250 mg $FeCl_3$ (1.54 mmol) and 3 mg $Pd(OAc)_2$ (0.0133 mmol) were added, 4 mL dry THF were added under a stream of dry argon. The mixture was stirred for 15 min. While maintaining a dry atmosphere at rt, 3.1 mL of 1 M solution of MeMgCl in THF was very slowly (1 drop/2 sec) added to the reaction mixture. After complete addition of the Grignard reagent, the mixture was stirred for an additional 15 min at rt. THF were removed by rotary evaporator, and the solid-state Fe/ppm Pd NPs-2 were transferred and stored in glove box.

General procedure for preparation of Fe NPs-3

In an oven dried 25 mL microwave reaction vial purged with argon, covered with a rubber septum containing a PTFE-coated magnetic stir bar, 250 mg $FeCl_3$ (1.54 mmol), 632 mg SPhos (1.54 mmol) and 4 mL dry THF were added under a stream of dry argon. The mixture was stirred for 15 min. While maintaining a dry atmosphere at rt, 3.1 mL of 1 M solution of MeMgCl in THF was very slowly (1 drop/2 sec) added to the reaction mixture. After complete addition of the Grignard reagent, the mixture was stirred for an additional 15 min at rt. THF were removed by rotary evaporator, and the solid-state Fe NPs-3 were transferred and stored in glove box.

Table S1. Results from variations in NP-catalyzed Suzuki-Miyaura reactions

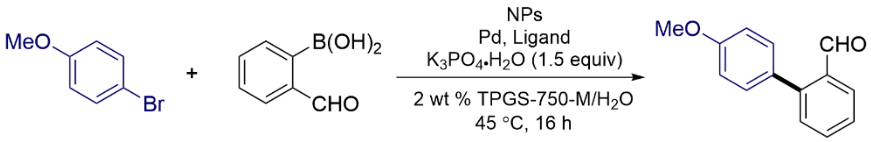

Entry[a]	NPs composition	Pd[b]	Ligand[b]	Yield[c]
A	5 % $FeCl_3$ (NPs-1)	320 ppm $Pd(OAc)_2$	3 % SPhos	71
B	5 % $FeCl_3$, 320 ppm $Pd(OAc)_2$ (NPs-2)	-	3 % SPhos	42
C	5 % $FeCl_3$, 3 % SPhos (NPs-3)	320 ppm $Pd(OAc)_2$	-	11

[a] Conditions: 4-bromoanisole (0.2 mmol), (2-formylphenyl)boronic acid (0.3 mmol), K_3PO_4•H_2O (0.3 mmol), NPs, Pd, Ligand, 2 wt % TPGS-750-M/H_2O (0.4 mL), 45 °C ,16 h

[b] Added directly into reaction vessel

[c] Isolated yield

Additional optimization followed the procedure using only Fe NPs-1

Optimization of reaction conditions for a Suzuki-Miyaura reaction

Table S2. Optimization of Pd and Ligand loadings for a Suzuki-Miyaura reaction

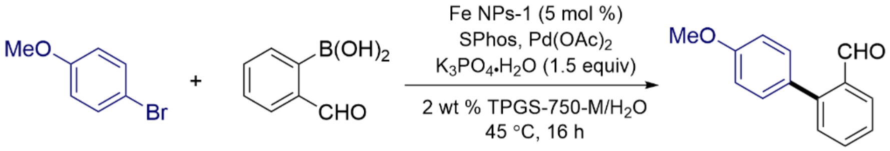

Entry[a]	Pd	Ligand	Yield
A	320 ppm $Pd(OAc)_2$	3 % SPhos	71[b]
B	320 ppm $Pd(OAc)_2$	5 % SPhos	87[c]
C	320 ppm $Pd(OAc)_2$	7 % SPhos	82[c]
D	320 ppm $Pd(OAc)_2$	10 % SPhos	88[c]
E	500 ppm $Pd(OAc)_2$	5 % SPhos	99[b]

[a] Conditions: 4-bromoanisole (0.2 mmol), (2-formylphenyl)boronic acid (0.3 mmol), K_3PO_4•H_2O (0.3 mmol), Fe NPs-1 (8 mg, containing 5 mol % $FeCl_3$), $Pd(OAc)_2$, SPhos, 2 wt % TPGS-750-M/H_2O (0.4 mL), 45 °C ,16 h

[b] Isolated yield

[c] Yield by 1H NMR, ethylene carbonate as internal standard

Optimization of conditions for a Sonogashira reaction

Table S3. Optimization for a Sonogashira reaction

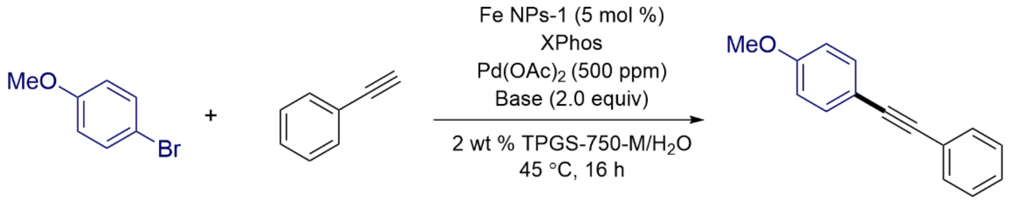

Entry[a]	Base	Equiv. of alkyne	Amount of XPhos	Yield[b]
A	K_3PO_4	1.5 equiv.	5 mol %	62
B	Et_3N	1.5 equiv.	5 mol %	66
C	DIPEA	1.5 equiv.	5 mol %	74
D	DIPEA	2.0 equiv.	5 mol %	83
E	DIPEA	2.0 equiv.	10 mol %	70
F	DIPEA	2.0 equiv.	3 mol %	99(98)
G	DIPEA	2.0 equiv.	1 mol %	96

[a] Conditions: 4-bromoanisole (0.2 mmol), ethynylbenzene, Base (0.4 mmol), Fe NPs-1 (8 mg, containing 5 mol % $FeCl_3$), $Pd(OAc)_2$ (500 ppm), Sphos, 2 wt % TPGS-750-M/H_2O (0.4 mL), 45 °C ,16 h

[b] Yield determined by HPLC,1-fluoro-4-(trifluoromethyl)benzene as internal standard, isolated yield in parentheses

Optimization on ferrocene-based ligands on four different reactions

Table S4. Optimization on ferrocene-based ligands on four different reactions

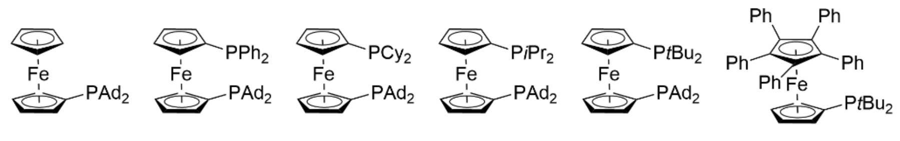

LS1 LS2 LS3 LS4 LS5 QPhos

reaction type	product	Conversion with LS1[e]	Conversion with LS2[e]	Conversion with LS3[e]	Conversion with LS4[e]	Conversion with LS5[e]	Conversion with QPhos[e]
Suzuki-Miyaura[a]	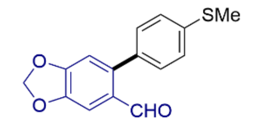	**>95**	<5	10	15	<5	86
Sonogashira[b]	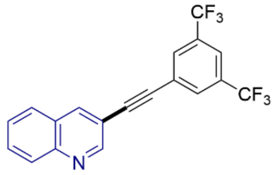	<5	<5	<5	<5	<5	40
Mizoroki-Heck[c]	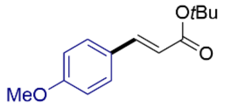	>95	>95	>95	>95	>95	80
	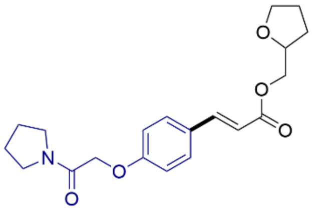	72	82	80	92	**>95**	-
Negishi[d]	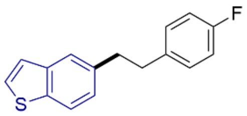	<5	<5	<5	<5	<5	<5

[a] Aryl bromide (0.2 mmol), Ar'-BR2 (0.3 mmol), Fe NPs (8 mg, 5 % Fe NPs), Pd(OAc)2 (500 ppm) in pot, Ligand (5 mol %), K3PO4·H2O (0.3 mmol), 2 wt % TPGS-750-M/H2O (0.4 mL), 45 °C, 24 h;

[b] Aryl bromide (0.2 mmol), alkyne (0.3 mmol), Fe NPs (8 mg, 5 % Fe NPs), Pd(OAc)2 (500 ppm) in pot, Ligand (5 mol %), DIPEA (0.4 mmol), 2 wt % TPGS-750-M/H2O (0.4 mL), 45 °C, 24 h;

[c] Aryl iodide (0.2 mmol), alkene (0.4 mmol), Fe NPs (2.8 mg, 5 % Fe NPs), 2500 ppm Pd(tBu3P)2 in pot, Ligand (2.5 mol %), K3PO4 (0.6 mmol), NaCl (1.2 mmol), 2 wt % TPGS-750-M/H2O (0.4 mL), DMF(0.04 mL), 45 °C, 40 h;

[d] Aryl bromide (0.2 mmol), alkyl bromide (0.6 mmol), Fe NPs (8 mg, 5 % Fe NPs), 2500 ppm Pd(OAc)2 in pot, Ligand (5 mol %), Zn powder (0.8 mmol), TMEDA (1.0 mmol), 2 wt % TPGS-750-M/H2O (0.4 mL), 45 °C, 40 h;

[e] Conversion based on 1H NMR, 1,3,5-trimethoxy benzene as internal standard

Titration of MeMgCl in THF with LiCl/I_2

To an oven dried 25 mL round bottom flask, anhydrous LiCl (424 mg, 10 mmol) was added under an argon atmosphere in the glovebox. The flask was sealed with a rubber septum, and 20 mL dry THF was added by syringe and the mixture was stirred at rt until the LiCl was completely dissolved, resulting in the formation of a 0.5 M solution of LiCl in THF.

A 10 mL microwave vial equipped with a magnetic stirring bar and a septum was heated with a heat gun under reduced pressure and cooled to rt under an argon atmosphere. In the glovebox, the dry microwave vial was charged with accurately weighed I_2 (127 mg, 0.5 mmol) and capped with a rubber septum. The saturated solution of LiCl in THF (2 mL) was added and stirring was started. After the iodine was completely dissolved, the resulting brown solution was cooled to 0 °C in an ice bath. Another 5 mL round bottom flask equipped with a magnetic stirring bar and a septum was heated with a heat gun under reduced pressure and cooled to rt under an argon atmosphere. To this round bottom flask, methyl magnesium chloride solution (from Sigma-Aldrich, catalog No. 189901; 3 mL) and dry THF (6 mL) were added by syringe and then stirred. To the vial with I_2, the methyl magnesium chloride solution was added dropwise via a 1.00 mL syringe (0.01 mL graduation) until the brown color disappeared. The amount consumed contains 1 equiv of the methyl magnesium chloride relative to iodine. The MeMgCl solution was titrated five times.

General procedure for Suzuki-Miyaura coupling reactions

To a flame dried 1-dram vial equipped with an oven dried stir bar was added aryl bromide (0.2 mmol, 1 equiv), organoboron compound (0.3 mmol, 1.5 equiv) and K_3PO_4•H_2O (69.2 mg, 0.3 mmol, 1.5 equiv). The vial was then transferred inside of a glove box. Fe NPs (8.0 mg, 5 mol % $FeCl_3$) and SPhos (4.1 mg, 5 mol %, or another ligand) was added into the vial in the glovebox. The vial was then sealed with a rubber septum inside of the glovebox. 500 ppm of $Pd(OAc)_2$ was added as a stock solution in THF, followed by the addition of 0.4 mL 2 wt % TPGS-750-M/H_2O solution by syringe and the mixture was stirred vigorously

at 45 °C for 16 h (unless otherwise noted). Then EtOAc was added, and the mixture stirred gently for 2 min at rt. Stirring was then stopped and the organic layer was decanted via pipette after centrifugation. The same extraction procedure was repeated twice. The combined organic extracts were dried under reduced pressure and purified by flash chromatography over silica gel.

General procedure for Sonogashira coupling reactions

To a flame dried 1-dram vial equipped with an oven dried stir bar was added aryl halide (0.2 mmol, 1 equiv). The vial was then transferred inside of a glove box. Fe NPs (8.0 mg, 5 mol % $FeCl_3$) and XPhos (2.8 mg, 3 mol %, or another ligand) was added into the vial in the glovebox. The vial was then sealed with a rubber septum inside of the glovebox. 500 ppm of $Pd(OAc)_2$ was added as a stock solution in THF, followed by the addition of alkyne (0.4 mmol, 2.0 equiv), DIPEA (0.07 mL, 0.4 mmol, 2.0 equiv) and 0.4 mL 2 wt % TPGS-750-M/H_2O solution by syringe and the mixture was stirred vigorously at 45 °C for 16 h (unless otherwise noted). Then EtOAc was added, and the mixture stirred gently for 2 min at rt. Stirring was then stopped and the organic layer was decanted via pipette after centrifugation. The same extraction procedure was repeated twice. The combined organic extracts were dried under reduced pressure and purified by flash chromatography over silica gel.

General procedure for Mizoroki-Heck coupling reactions

To a flame dried 1-dram vial equipped with an oven dried stir bar was added aryl iodide (0.2 mmol, 1 equiv), alkene (if it is solid), NaCl (70.2 mg, 1.2 mmol, 6 equiv) and K_3PO_4 (127.4 mg, 0.6 mmol, 3 equiv). The vial was then transferred inside of a glove box. Fe NPs (2.8 mg, 1.8 mol % $FeCl_3$) and tBu$_3$P (1.5 mg, 2.5 mol %; or another ligand) was added into the vial in the glovebox. The vial was then sealed with a rubber septum inside of the glovebox. 2500 ppm of Pd(tBu$_3$P)$_2$ was added as a stock solution in DCM. The DCM was removed by vacuum and the vial was refilled with argon. The vial was then added 0.4 mL 2 wt % TPGS-750-M/H_2O solution, alkene (if it is liquid) and 0.04 mL DMF by syringe and the mixture was stirred

vigorously at 45 °C for 40 h (unless otherwise noted). Then EtOAc was added, and the mixture stirred gently for 2 min at rt. Stirring was then stopped and the organic layer was decanted via pipette after centrifugation. The same extraction procedure was repeated twice. The combined organic extracts were dried under reduced pressure and purified by flash chromatography over silica gel.

General procedure for Negishi coupling reactions

To a flame dried 1-dram vial equipped with an oven dried stir bar was added aryl bromide (0.2 mmol, 1 equiv). The vial was then transferred inside of a glove box. Fe NPs (8.0 mg, 5 mol % $FeCl_3$), AmPhos (2.7 mg, 5 mol %; or another ligand) and zinc powder (52 mg, 0.8 mmol, 4 equiv) was added into the vial in the glovebox. The vial was then sealed with a rubber septum inside of the glovebox. 2500 ppm of $Pd(OAc)_2$ was added as a stock solution in THF, followed by the addition of alkyl bromide (0.8 mmol, 4 equiv), TMEDA (0.15 mL, 1.0 mmol, 5 equiv) and 1.0 mL 2 wt % TPGS-750-M/H_2O solution by syringe and the mixture was stirred vigorously at 45 °C for 40 h (unless otherwise noted). Then EtOAc was added, and the mixture stirred gently for 2 min at rt. Stirring was then stopped and the organic layer was decanted via pipette after centrifugation. The same extraction procedure was repeated twice. The combined organic extracts were dried under reduced pressure and purified by flash chromatography over silica gel.

Analyses of nanoparticles (NPs)

STEM images for new Fe NPs with SPhos

The Fe NPs (4 mg) and SPhos (4.1 mg, 0.01 mmol) was added to a vial in the glove box, and the vial was covered with a septum. 500 ppm $Pd(OAc)_2$ (0.0225 mg, 0.0001 mmol) were added by stock solution in THF. 2 wt % TPGS-750-M/H_2O (2 mL) was inserted into the vial via syringe and the mixture was stirred for 15 min.

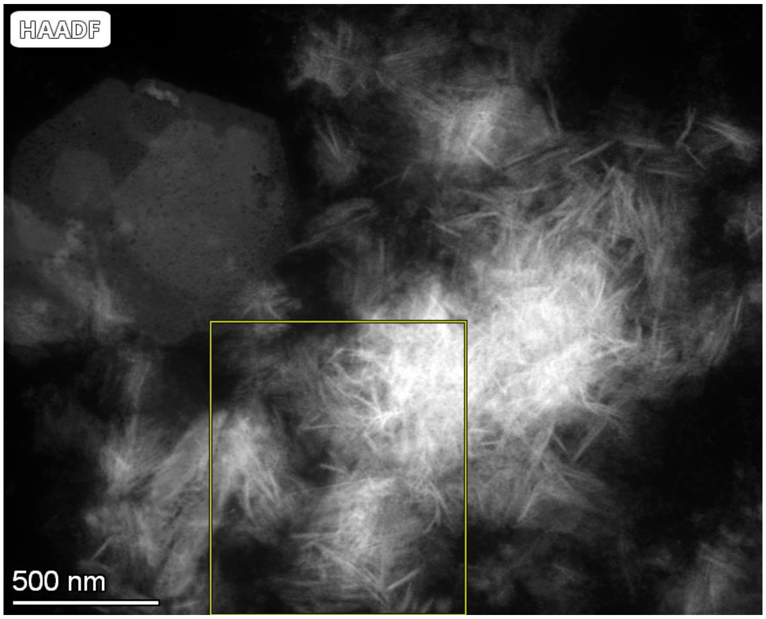

Figure S1, HAADF image showing new Fe NPs with SPhos in 2 wt % TPGS-750-M/H_2O in a 500 nm scale

Energy-Dispersive X-ray Spectroscopy (EDS) analysis for new Fe NPs with SPhos

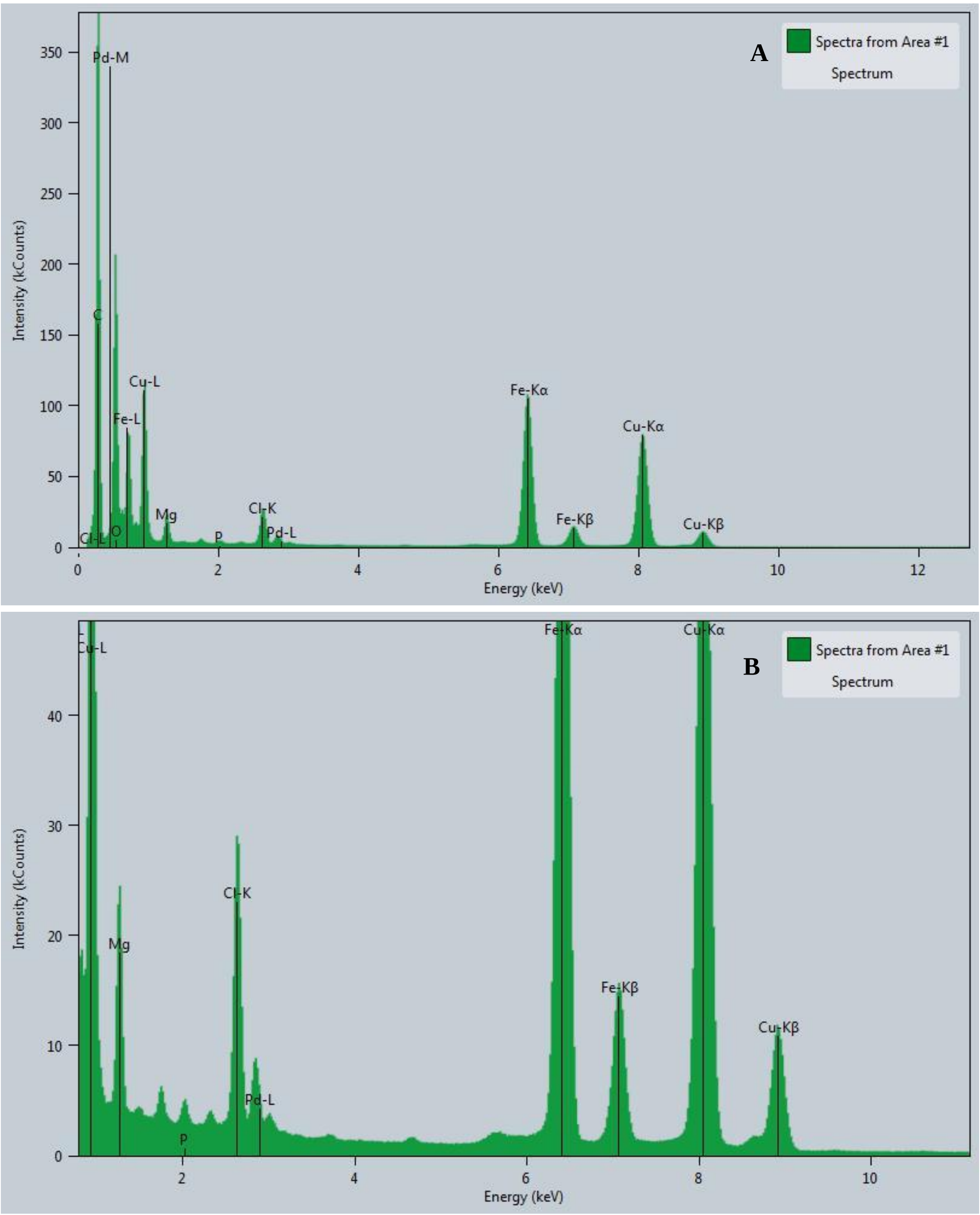

Figure S2, EDS analysis showing new Fe NPs with SPhos in 2 wt % TPGS-750-M/H_2O

STEM images for Fe/ppm Pd NPs with SPhos from original recipe

The Fe/ppm Pd NPs were synthesized as reported.[1] The Fe/ppm Pd NPs (4 mg) was added to a vial in the glove box, and the vial was covered with a septum. 2 wt % TPGS-750-M/H_2O (2 mL) was inserted into the vial via syringe and the mixture was stirred for 15 min.

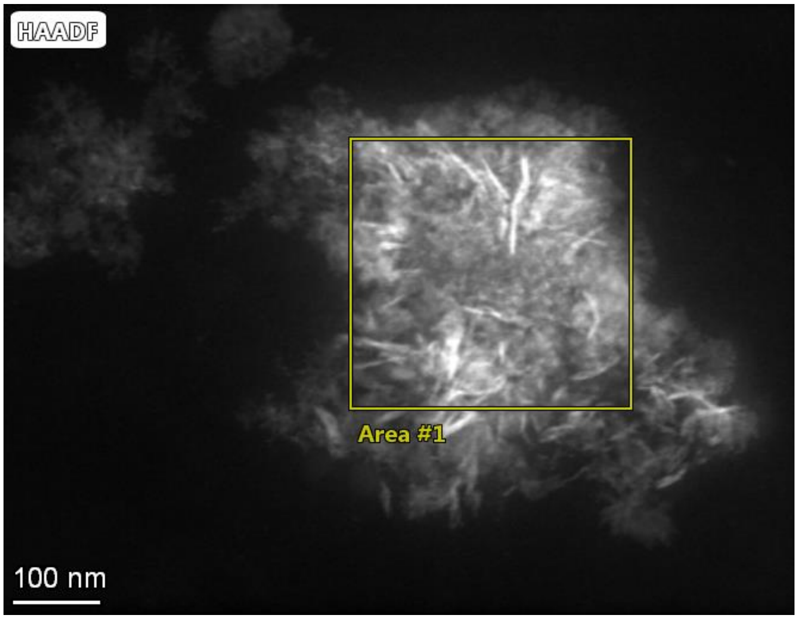

Figure S3, HAADF image showing original Fe/ppm Pd NPs with SPhos in 2 wt % TPGS-750-M/H_2O in a 100 nm scale

EDS analysis for Fe/ppm Pd NPs with SPhos from the original recipe[1]

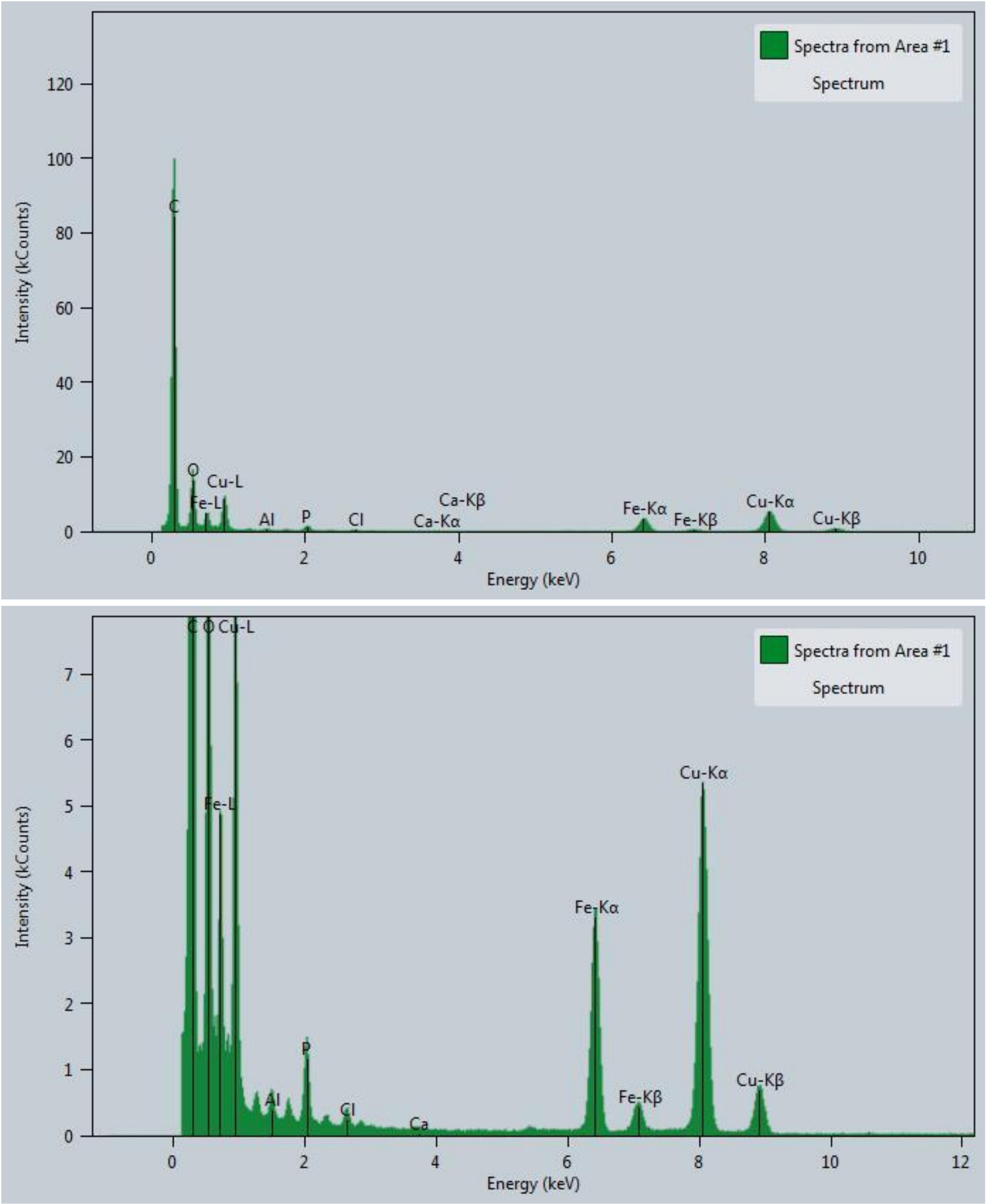

Figure S4, EDS analysis showing original Fe/ppm Pd NPs with SPhos in 2 wt % TPGS-750-M/H_2O

E Factor, gram scale, and recycling studies

E Factor

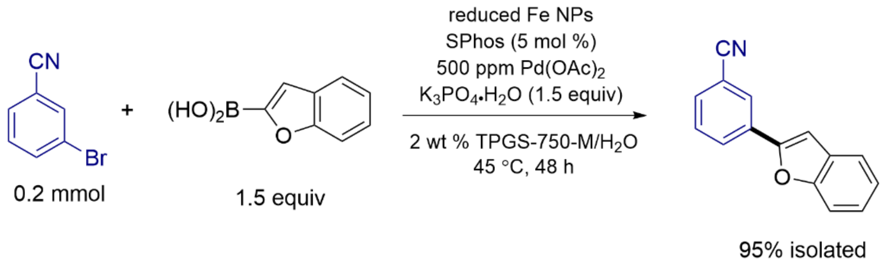

To a flame dried 1-dram vial equipped with an oven dried stir bar was added 3-bromobenzonitrile (36.4 mg, 0.2 mmol, 1 equiv), benzofuran-2-ylboronic acid (48.6 mg, 0.3 mmol, 1.5 equiv) and $K_3PO_4{\bullet}H_2O$ (69.2 mg, 0.3 mmol, 1.5 equiv). The vial was then transferred inside of a glove box. Fe NPs (8.0 mg, 5 mol % $FeCl_3$) and SPhos (4.1 mg, 5 mol %) was added into the vial in the glovebox. The vial was then sealed with a rubber septum inside of the glovebox. 500 ppm of $Pd(OAc)_2$ was added as a stock solution in THF, followed by the addition of 0.4 mL 2 wt % TPGS-750-M/H_2O solution by syringe and the mixture was stirred vigorously at 45 °C for 48 h. Stirring was then stopped and the liquid was decanted carefully via pipette after centrifugation. The solid were dried under reduced pressure and purified by flash chromatography over silica gel to get 3-(benzofuran-2-yl)benzonitrile (41.7 mg, 95 %) as a white solid.

E Factor calculation:

$$\text{E Factor (without water)} = \frac{\text{mass of organic waste}}{\text{mass of product}}$$

$$= \frac{\text{mass of excess boronic acid}}{\text{mass of product}}$$

$$= \frac{16.2\text{ mg}}{41.7\text{ mg}}$$

$$= 0.39$$

$$\text{E Factor (with water)} = \frac{\text{mass of organic waste + mass of water}}{\text{mass of product}}$$

$$= \frac{\text{mass of excess boronic acid + mass of water}}{\text{mass of product}}$$

$$= \frac{16.2\text{ mg} + 400\text{ mg}}{41.7\text{ mg}}$$

$$= 9.98$$

Gram scale reaction

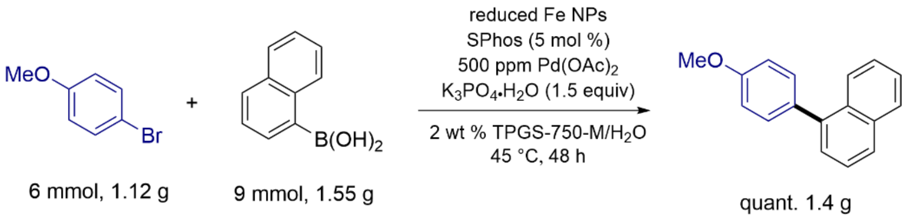

To a oven dried 50 mL round bottom flask with an oven dried stir bar was added naphthalen-1-ylboronic acid (1.55 g, 9 mmol, 1.5 equiv), $K_3PO_4{\bullet}H_2O$ (2.08 g, 9 mmol, 1.5 equiv). The flask was then transferred inside of a glove box. Fe NPs (240 mg, 5 mol % $FeCl_3$) and SPhos (123 mg, 5 mol %) was added into the vial in the glovebox. The vial was then sealed with a rubber septum inside of the glovebox. 500 ppm of $Pd(OAc)_2$ was added as a stock solution in THF, followed by the addition of 12 mL 2 wt % TPGS-750-M/H_2O solution by syringe and the mixture was stirred vigorously at 45 °C for 48 h. Then EtOAc was added, and the mixture stirred gently for 2 min at rt. Stirring was then stopped and the mixture were transferred into separatory funnel. The organic layer was collected. The same extraction procedure was repeated twice. The combined organic extracts were dried under reduced pressure and purified by flash chromatography over silica gel to get 1-(4-methoxyphenyl)naphthalene (1.4 g, quant.) as a white solid.

Recycling reactions

Scheme S1, recycle study

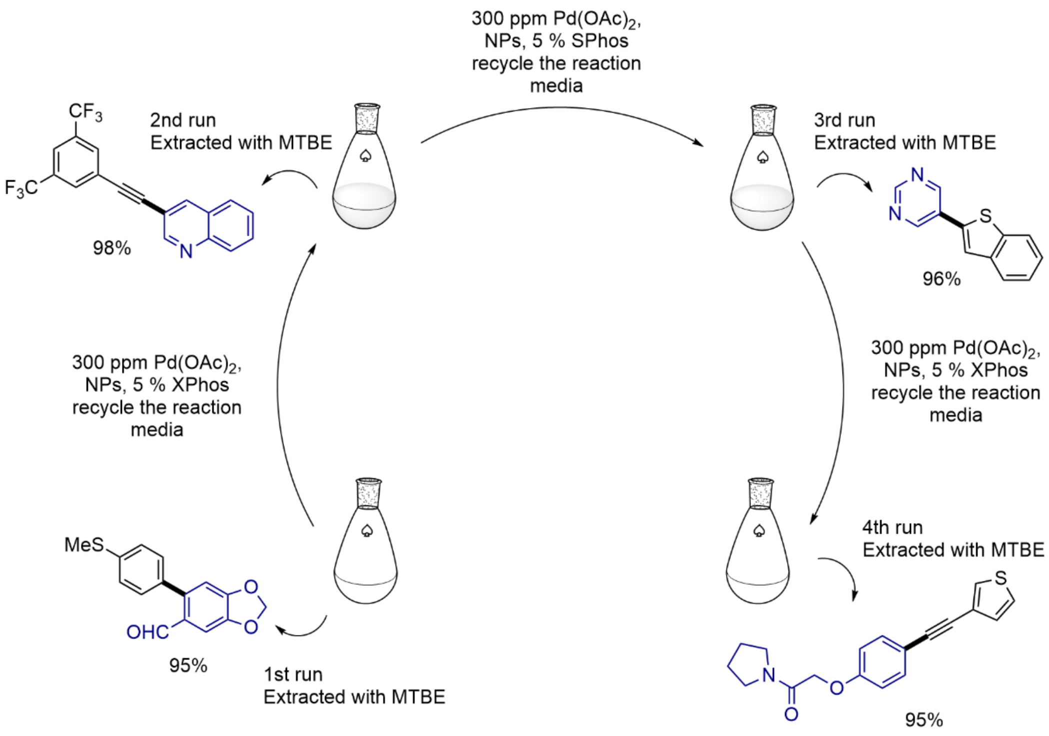

1st run:

To a flame dried 1-dram vial equipped with an oven dried stir bar was added 6-bromobenzo[d][1,3]dioxole-5-carbaldehyde (0.2 mmol, 45.8 mg, 1 equiv), (4-(methylthio)phenyl)boronic acid (0.3 mmol, 50.4 mg, 1.5 equiv) and K_3PO_4•H_2O (69.2 mg, 0.3 mmol, 1.5 equiv). The vial was then transferred inside of a glove box. Fe NPs (8.0 mg, 5 mol % $FeCl_3$) and SPhos (4.1 mg, 5 mol %) was added into the vial in the glovebox. The vial was then sealed with a rubber septum inside of the glovebox. 500 ppm of $Pd(OAc)_2$ was added as a stock solution in THF, followed by the addition of 0.4 mL 2 wt % TPGS-750-M/H_2O solution by syringe and the mixture was stirred vigorously at 45 °C for 16 h. Then, 0.4 mL MTBE was added and the mixture stirred gently for 2 min at rt. Stirring was then stopped and the organic layer was decanted via pipette after centrifugation. The same extraction procedure was repeated twice. The combined organic extracts were

dried under reduced pressure and purified by flash chromatography over silica gel with Hex/EtOAc: 95/5 to obtain 6-(4-(methylthio)phenyl)benzo[d][1,3]dioxole-5-carbaldehyde. (51.7 mg, 95%).

2nd run:

To the same vial, Fe NPs (8 mg, 5 mol % $FeCl_3$), XPhos (2.8 mg. 3 mol %) were added under argon. The vial was then sealed with a rubber septum under an argon flow. Then, 3-bromoquinoline (27 μL, 0.2 mmol, 1 equiv), 1-ethynyl-3,5-bis(trifluoromethyl)benzene (0.07 mL, 0.4 mmol, 2 equiv), and DIPEA (0.07 mL, 0.4 mmol, 2 equiv) were added to the vial via syringe. 300 ppm of $Pd(OAc)_2$ was added as a stock solution in THF. The vial was then stirred vigorously at 45 °C for 16 h. Then, 0.4 mL MTBE was added, and the mixture stirred gently for 2 min at rt. Stirring was then stopped and the organic layer was decanted via pipette after centrifugation. The same extraction procedure was repeated twice. The combined organic extracts were dried under reduced pressure and purified by flash chromatography over silica gel with hexanes/EtOAc : 95/5 to obtain 3-((3,5-bis(trifluoromethyl)phenyl)ethynyl)quinoline. (71.4 mg, 98%).

3rd run:

To the same vial, Fe NPs (8 mg, 5 mol % $FeCl_3$), SPhos (4.1 mg. 5 mol %), 5-bromopyrimidine (31.8 mg, 0.2 mmol, 1 equiv), benzo[b]thiophen-2-ylboronic acid (53.4 mg, 0.3 mmol, 1.5 equiv) and $K_3PO_4{\bullet}H_2O$ (69.2 mg, 0.3 mmol, 1.5 equiv) were added under argon. The vial was then sealed with a rubber septum under an argon flow. Then 300 ppm of $Pd(OAc)_2$ was added as a stock solution in THF. The vial was then stirred vigorously at 45 °C for 16 h. Then, 0.4 mL MTBE was added, and the mixture stirred gently for 2 min at rt. Stirring was then stopped and the organic layer was decanted via pipette after centrifugation. The same extraction procedure was repeated twice. The combined organic extracts were dried under reduced pressure and purified by flash chromatography over silica gel with hexanes/EtOAc:70/30 to 5-(benzo[b]thiophen-2-yl)pyrimidine. (40.3 mg, 95%).

4th run:

To the same vial, Fe NPs (8 mg, 5 mol % $FeCl_3$), XPhos (2.8 mg. 3 mol %) and 2-(4-iodophenoxy)-1-(pyrrolidin-1-yl)ethan-1-one (66.2 mg, 0.2 mmol, 1 equiv) were added under argon. The vial was then sealed with a rubber septum under an argon flow. Then 3-ethynylthiophene (39 μL, 0.4 mmol, 2 equiv) and DIPEA (0.07 mL, 0.4 mmol, 2 equiv) were added to the vial via syringe. 300 ppm of $Pd(OAc)_2$ was added as a stock solution in THF followed by 0.15 mL 2 wt % TPGS-750-M aqueous solution to maintain the solvent volume. The vial was then stirred vigorously at 45 °C for 16 h. Then, 0.4 mL MTBE was added, and the mixture stirred gently for 2 min at rt. Stirring was then stopped and the organic layer was decanted via pipette after centrifugation. The same extraction procedure was repeated twice. The combined organic extracts were dried under reduced pressure and purified by flash chromatography over silica gel with hexanes/EtOAc:75/25 to obtain 1-(pyrrolidin-1-yl)-2-(4-(thiophen-3-ylethynyl)phenoxy)ethan-1-one. (59.6 mg, 96%).

1-Pot sequence of reactions

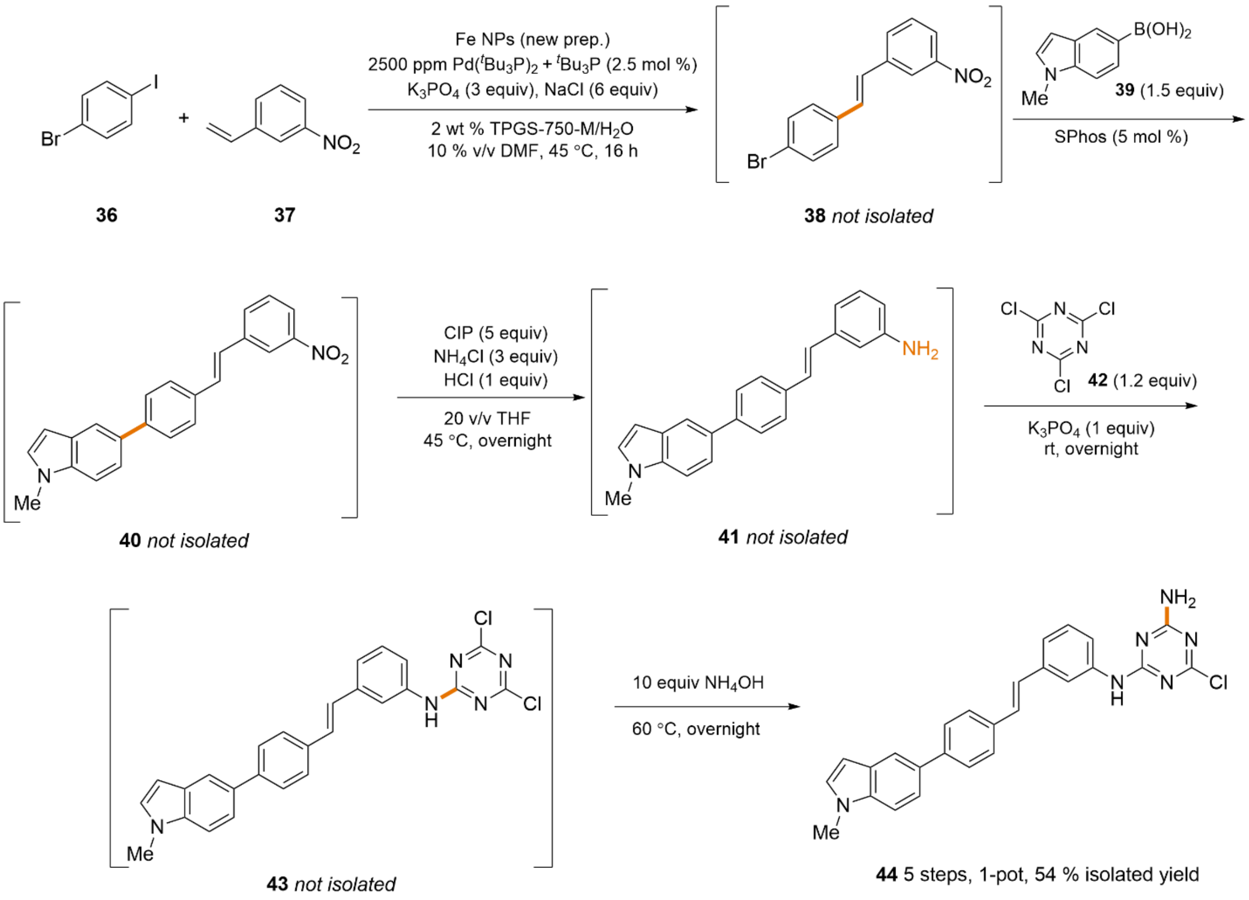

Scheme S2. Tandem reaction sequence in water: 5-steps, 1-pot

Step 1: To a flame dried 1-dram vial equipped with an oven dried stir bar was added 1-bromo-4-iodobenzene (141.5 mg, 0.5 mmol), K_3PO_4 (318 mg, 1.5 mmol, 3 equiv), and NaCl (175.5 mg, 3.0 mmol, 6 equiv) and the vial was then transferred into a glove box to which was added P(*t*Bu)$_3$ (2.5 mg, 0.0125 mmol, 2.5 mol %) inside of an argon-purged glove box. The vial was sealed with a rubber septum and removed from the glove box. Then, Pd(*t*Bu$_3$P)$_2$ (0.64 mg, 0.00125 mmol, 2500 ppm) were added by stock solution in DCM. The DCM was removed by vacuum and the vial was refilled with argon. 1-Nitro-3-vinylbenzene (0.14 mL, 1.0 mmol, 2 equiv), DMF (0.1 mL) and 2 wt % TPGS-750-M/H_2O (1.0 mL) was

added to the vial via syringe and the vial were stirred vigorously under constant argon pressure at 45 °C for 16 h. The progress of the reaction was monitored by TLC.

Step 2: After complete consumption of starting material, the septum was removed. SPhos (10.3 mg, 0.025 mmol, 5 mol %) and (1-methyl-1H-indol-5-yl)boronic acid (131.3 mg, 0.75 mmol, 1.5 equiv) was added to the vial under argon flow. The vial was sealed with a rubber septum and stirred vigorously under constant argon pressure at 45 °C for overnight. The progress of the reaction was monitored by TLC.

Step 3: After complete consumption of starting material, the septum was removed. Carbonyl iron powder (CIP; 140 mg, 2.5 mmol, 5 equiv) and NH_4Cl (80.3 mg, 1.5 mmol, 3 equiv) were added to the vial under an argon flow. The vial was sealed with a rubber septum. 1 M HCl (0.5 mL, 0.5 mmol, 1 equiv) and THF (0.2 mL) were added via syringe and the contents of the vial were stirred vigorously under constant argon pressure at 45 °C for overnight. The progress of the reaction was monitored by TLC.

Step 4: After complete consumption of starting material, the septum was removed. 2,4,6-Trichloro-1,3,5-triazine (110 mg, 0.6 mmol, 1.2 equiv) and K_3PO_4 (106 mg, 0.5 mmol, 1 equiv) were added to the vial under an argon flow. The vial was sealed with a rubber septum and the contents stirred vigorously under constant argon pressure at rt overnight. The progress of the reaction was monitored by TLC.

Step 5: After complete consumption of starting material, the septum was removed. 28% NH_4OH aqueous solution (0.33 mL, 5 mmol, 10 equiv) was added via syringe and the contents of the vial were stirred vigorously under constant argon pressure at 60 °C for overnight. The reaction was then monitored by thin-layer chromatography until completion. Then, 2.0 mL EtOAc was added to the mixture after which it was stirred *gently* for 2 min at rt. Stirring was then stopped and the organic layer was decanted via a pipette after centrifugation. The same extraction procedure was repeated four times. The combined organic extracts were dried over anhydrous Na_2SO_4 and the solvent removed *in vacuo*, with the resulting crude material being purified by flash chromatography over silica gel with EtOAc/hexanes: 50/50 to afford (*E*)-6-chloro-N^2-(3-(4-(1-methyl-1*H*-indol-5-yl)styryl)phenyl)-1,3,5-triazine-2,4-diamine (122.2 mg, 54% overall yield) as a red solid.

Analytical data for products

2,2-Difluoro-5-(3-((2-fluorobenzyl)oxy)phenyl)benzo[d][1,3]dioxole (4)

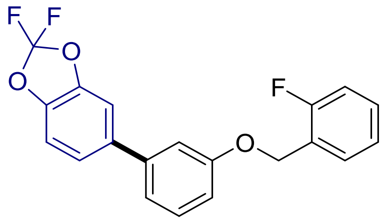

5-Bromo-2,2-difluorobenzo[d][1,3]dioxole (47.4 mg, 27 μL, 0.2 mmol), (3-((2-fluorobenzyl)oxy)phenyl)-boronic acid (121.8 mg, 0.3 mmol), $Pd(OAc)_2$ (0.0225 mg, 0.0001 mmol), SPhos (4.1 mg, 0.01 mmol), Fe NPs (8 mg, 5% $FeCl_3$) and $K_3PO_4{\cdot}H_2O$ (69.2 mg, 0.3 mmol) in 0.4 mL 2 wt % TPGS-750-M/H_2O were reacted at 45 °C for 24 h yielding 68.1 mg (95%) of 2,2-difluoro-5-(3-((2-fluorobenzyl)oxy)phenyl)-benzo[d][1,3]dioxole as a yellow oil (hexanes/EtOAc: 90/10).

^{1}H NMR (400 MHz, chloroform-*d*) δ 7.35 – 7.28 (m, 4H), 7.25 (dd, *J* = 7.7, 2.1 Hz, 1H), 7.12 – 7.05 (m, 3H), 7.04 – 6.97 (m, 3H), 5.09 (s, 2H).

^{13}C NMR (101 MHz, chloroform-*d*) δ 164.31, 161.86, 155.27, 143.66, 142.93, 139.64 (d, $J_{(C\text{-}F)}$ = 8 Hz), 134.59, 131.07, 130.24 (d, $J_{(C\text{-}F)}$ = 9 Hz), 130.11, 129.25, 124.99, 122.32 (d, $J_{(C\text{-}F)}$ = 3 Hz), 121.81, 114.82 (d, $J_{(C\text{-}F)}$ = 21.2 Hz), 113.88 (d, $J_{(C\text{-}F)}$ = 22.2 Hz), 113.44, 111.15, 109.02, 69.92 (d, $J_{(C\text{-}F)}$ = 2 Hz).

^{19}F NMR (376 MHz, chloroform-*d*) δ -49.94, -112.70.

HRMS(EI): Calcd. for $C_{20}H_{13}F_3O_3^+$: 358.0817 Found: 358.0816.

6-(4-(Methylthio)phenyl)benzo[d][1,3]dioxole-5-carbaldehyde (5)

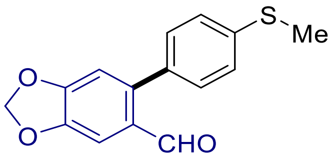

6-Bromobenzo[d][1,3]dioxole-5-carbaldehyde (45.8 mg, 0.2 mmol), (4-(methylthio)phenyl)boronic acid (50.4 mg, 0.3 mmol), $Pd(OAc)_2$ (0.0225 mg, 0.0001 mmol), SPhos (4.1 mg, 0.01 mmol), Fe NPs (8 mg, 5% $FeCl_3$) and $K_3PO_4{\cdot}H_2O$ (69.2 mg, 0.3 mmol) in 0.4 mL 2 wt % TPGS-750-M/H_2O were reacted at 45 °C for 24 h yielding 50.8 mg (93 %) of 6-(4-(methylthio)phenyl)benzo[d][1,3]dioxole-5-carbaldehyde as a white solid (hexanes/EtOAc : 90/10).

^{1}H NMR (400 MHz, chloroform-*d*) δ 9.75 (s, 1H), 7.45 (s, 1H), 7.31 (d, *J* = 8.4 Hz, 2H), 7.25 (d, *J* = 8.4 Hz, 2H), 6.81 (s, 1H), 6.08 (s, 2H), 2.53 (s, 3H).

^{13}C NMR (126 MHz, chloroform-*d*) δ 190.63, 152.25, 147.89, 143.12, 139.25, 134.17, 130.60, 128.93, 126.19, 110.24, 106.48, 102.23, 15.67.

Spectral data matched those previously reported.[2]

3-(Benzofuran-2-yl)benzonitrile (6)

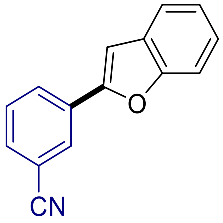

3-Bromobenzonitrile (36.4 mg, 0.2 mmol), benzofuran-2-MIDA boronate (81.9 mg, 0.3 mmol), $Pd(OAc)_2$ (0.0225 mg, 0.0001 mmol), SPhos (4.1 mg, 0.01 mmol), Fe NPs (8 mg, 5% $FeCl_3$) and $K_3PO_4{\cdot}H_2O$ (69.2 mg, 0.3 mmol) in 0.4 mL 2 wt % TPGS-750-M/H_2O were reacted at 45 °C for 24 h yielding 42.0 mg (96%) of 3-(benzofuran-2-yl)benzonitrile as a white solid (hexanes/EtOAc : 95/5).

^{1}H NMR (400 MHz, chloroform-*d*) δ 7.98 (t, *J* = 1.5 Hz, 1H), 7.91 (dt, *J* = 7.8, 1.5 Hz, 1H), 7.83 (ddd, *J* = 16.6, 6.8, 2.5 Hz, 2H), 7.61 (m, 2H), 7.53 (t, *J* = 7.8 Hz, 1H), 7.42 – 7.34 (m, 2H).

^{13}C NMR (101 MHz, chloroform-*d*) δ 141.39, 140.44, 139.80, 135.77, 131.45, 130.67, 129.96, 129.89, 125.29, 125.06, 124.16, 122.53, 121.20, 118.60, 113.41.

Spectral data matched those previously reported.[3]

5-(Benzo[b]thiophen-2-yl)pyrimidine (7)

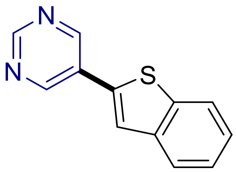

5-Bromopyrimidine (31.8 mg, 0.2 mmol), benzo[b]thiophen-2-ylboronic acid (53.4 mg, 0.3 mmol), $Pd(OAc)_2$ (0.0225 mg, 0.0001 mmol), SPhos (4.1 mg, 0.01 mmol), Fe NPs (8 mg, 5% $FeCl_3$) and K_3PO_4• H_2O (69.2 mg, 0.3 mmol) in 0.4 mL 2 wt % TPGS-750-M/H_2O were reacted at 45 °C for 24 h yielding 42.6 mg (100%) of 5-(benzo[b]thiophen-2-yl)pyrimidine as a yellow solid (hexanes/EtOAc : 70/30).

^{1}H NMR (400 MHz, chloroform-*d*) δ 9.17 (s, 1H), 9.04 (s, 2H), 7.85 (ddd, *J* = 14.2, 6.6, 3.0 Hz, 2H), 7.65 (s, 1H), 7.44 – 7.35 (m, 2H).

^{13}C NMR (126 MHz, chloroform-*d*) δ 157.92, 154.05, 140.21, 140.03, 136.20, 128.80, 125.60, 125.23, 124.27, 122.58, 122.05.

Spectral data matched those previously reported.[4]

5,5-Dimethyl-3-((5-(1-methyl-1H-indol-5-yl)pyridin-2-yl)oxy)-4-(4-(methylsulfonyl)phenyl)furan-2(5H)-one (8)

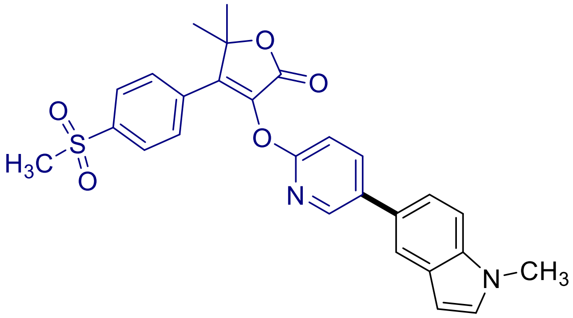

3-((5-Bromopyridin-2-yl)oxy)-5,5-dimethyl-4-(4-(methylsulfonyl)phenyl)furan-2(5H)-one (87.7 mg, 0.2 mmol), (1-methyl-1H-indol-5-yl)boronic acid (52.5 mg, 0.3 mmol), $Pd(OAc)_2$ (0.0338 mg, 0.00015 mmol), SPhos (4.1 mg, 0.01 mmol), Fe NPs (8 mg, 5% $FeCl_3$) and K_3PO_4•H_2O (69.2 mg, 0.3 mmol) in 0.4 mL 2

wt % TPGS-750-M/H_2O were reacted at 55 °C for 48 h yielding 82.1 mg (84%) of 5,5-dimethyl-3-((5-(1-methyl-1H-indol-5-yl)pyridin-2-yl)oxy)-4-(4-(methylsulfonyl)phenyl)furan-2(5H)-one as a white solid (hexanes/EtOAc : 75/25).

^{1}H NMR (400 MHz, chloroform-*d*) δ 8.37 (d, *J* = 2.3 Hz, 1H), 8.01 (d, *J* = 8.5 Hz, 2H), 7.95 (dd, *J* = 8.5, 2.5 Hz, 1H), 7.80 (d, *J* = 8.5 Hz, 2H), 7.75 (s, 1H), 7.44 – 7.34 (m, 2H), 7.10 (d, *J* = 3.1 Hz, 1H), 7.06 (d, *J* = 8.5 Hz, 1H), 6.54 (d, *J* = 3.0 Hz, 1H), 3.83 (s, 3H), 3.06 (s, 3H), 1.79 (s, 6H).

^{13}C NMR (101 MHz, chloroform-*d*) δ 165.97, 160.01, 148.24, 145.61, 141.31, 138.84, 138.07, 136.42, 135.08, 134.78, 129.84, 129.04, 129.02, 128.60, 127.92, 120.94, 119.36, 110.77, 109.83, 101.38, 84.36, 44.39, 32.98, 26.46.

HRMS(EI): Calcd. for $C_{27}H_{24}N_2O_5SH^+$: 489.1484 Found: 489.1488.

4'-Methoxy-[1,1'-biphenyl]-2-carbaldehyde (9)

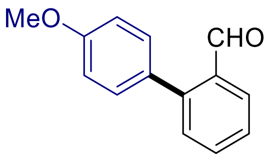

4-Bromoanisole (37.4 mg, 25 μL, 0.2 mmol), (2-formylphenyl)boronic acid (45.0 mg, 0.3 mmol), $Pd(OAc)_2$ (0.0225 mg, 0.0001 mmol), SPhos (4.1 mg, 0.01 mmol), Fe NPs (8 mg, 5% $FeCl_3$) and K_3PO_4• H_2O (69.2 mg, 0.3 mmol) in 0.4 mL 2 wt % TPGS-750-M/H_2O were reacted at 45 °C for 24 h yielding 42.0 mg (99%) 4'-methoxy-[1,1'-biphenyl]-2-carbaldehyde as a white solid (hexanes/EtOAc : 95/5).

^{1}H NMR (400 MHz, chloroform-*d*) δ 10.00 (s, 1H), 8.01 (d, *J* = 8.7 Hz, 1H), 7.62 (td, *J* = 7.5, 1.4 Hz, 1H), 7.50 – 7.41 (m, 2H), 7.31 (d, *J* = 8.7 Hz, 2H), 7.01 (d, *J* = 8.7 Hz, 2H), 3.88 (s, 3H).

^{13}C NMR (126 MHz, chloroform-*d*) δ 192.83, 159.84, 145.80, 133.90, 133.67, 131.44, 130.93, 130.16, 127.76, 127.52, 114.08, 55.54.

Spectral data matched those previously reported.[5]

3-((3,5-Bis(trifluoromethyl)phenyl)ethynyl)quinoline (10)

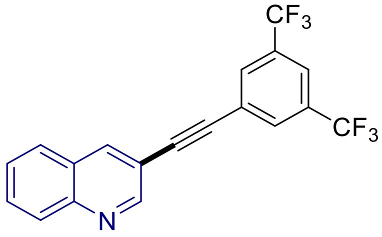

3-Bromoquinoline (47.4 mg, 27 μL, 0.2 mmol), 1-ethynyl-3,5-bis(trifluoromethyl)benzene (121.8 mg, 65 μL, 0.4 mmol), $Pd(OAc)_2$ (0.0225 mg, 0.0001 mmol), XPhos (2.9 mg, 0.006 mmol), Fe NPs (8 mg, 5% $FeCl_3$) and DIPEA (51.7 mg, 0.07 mL, 0.4 mmol) in 0.4 mL 2 wt % TPGS-750-M/H_2O were reacted at 45 °C for 24 h yielding 73.1 mg (100 %) of 3-((3,5-bis(trifluoromethyl)phenyl)ethynyl)quinoline as a yellow oil (hexanes/EtOAc : 80/20).

^{1}H NMR (600 MHz, chloroform-*d*) δ 9.01 (d, *J* = 2.1 Hz, 1H), 8.35 (d, *J* = 1.6 Hz, 1H), 8.12 (d, *J* = 8.4 Hz, 1H), 8.02 (s, 2H), 7.86 (s, 1H), 7.82 (d, *J* = 8.1 Hz, 1H), 7.79 – 7.74 (m, 1H), 7.60 (t, *J* = 7.3 Hz, 1H).
^{13}C NMR (126 MHz, chloroform-*d*) δ 151.82, 147.40, 139.21, 132.29 (q, $J_{(C-F)}$ = 34 Hz) , 131.70, 130.87, 129.66, 127.90, 127.21, 127.15, 125.14, 123.04 (q, $J_{(C-F)}$ = 274 Hz), 122.21 (hept, $J_{(C-F)}$ = 4 Hz), 116.16, 90.10, 89.43.
^{19}F NMR (376 MHz, chloroform-*d*) δ -63.14.
HRMS(EI): Calcd. for $C_{19}H_9F_6NH^+$: 366.0717 Found: 366.0722.

Methyl (2-(3-benzamidoprop-1-yn-1-yl)phenyl)carbamate (11)

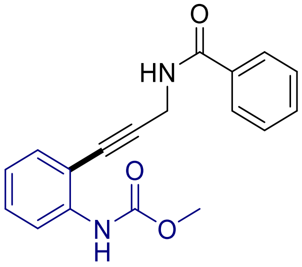

Methyl (2-bromophenyl)carbamate (46 mg, 0.2 mmol), *N*-(prop-2-yn-1-yl)benzamide (63.7 mg, 0.4 mmol), $Pd(OAc)_2$ (0.0225 mg, 0.0001 mmol), XPhos (2.9 mg, 0.006 mmol), Fe NPs (8 mg, 5% $FeCl_3$) and DIPEA (51.7 mg, 0.07 mL, 0.4 mmol) in 0.4 mL 2 wt % TPGS-750-M/H_2O were reacted at 45 °C for 24 h yielding 51.6 mg (84%) of methyl (2-(3-benzamidoprop-1-yn-1-yl)phenyl)carbamate as a yellow oil (hexanes/ EtOAc : 70/30).

^{1}H NMR (400 MHz, chloroform-*d*) δ 8.12 (d, *J* = 8.3 Hz, 1H), 7.82 (d, *J* = 7.3 Hz, 2H), 7.52 (t, *J* = 7.3 Hz, 1H), 7.44 (t, *J* = 7.4 Hz, 3H), 7.38 – 7.28 (m, 2H), 6.97 (t, *J* = 7.5 Hz, 1H), 6.63 (br, 1H), 4.52 (d, *J* = 5.3 Hz, 2H), 3.76 (s, 3H).

^{13}C NMR (126 MHz, chloroform-*d*) δ 167.42, 153.89, 139.55, 133.88, 132.04, 131.99, 130.03, 128.77, 127.21, 122.62, 117.99, 110.87, 92.08, 79.02, 52.52, 30.82.

HRMS(EI): Calcd. for $C_{18}H_{16}N_2O_3H^+$: 309.1239 Found: 309.1244.

1-(Pyrrolidin-1-yl)-2-(4-(thiophen-3-ylethynyl)phenoxy)ethan-1-one (12)

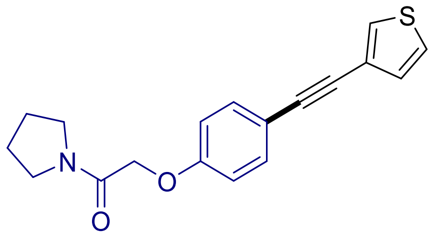

2-(4-Iodophenoxy)-1-(pyrrolidin-1-yl)ethan-1-one (66.2 mg, 0.2 mmol), 3-ethynylthiophene (43.3 mg, 39 μL, 0.4 mmol), $Pd(OAc)_2$ (0.0225 mg, 0.0001 mmol), XPhos (2.9 mg, 0.006 mmol), Fe NPs (8 mg, 5% $FeCl_3$) and DIPEA (51.7 mg, 0.07 mL, 0.4 mmol) in 0.4 mL 2 wt % TPGS-750-M/H_2O were reacted at 45 ° C for 24 h yielding 62.4 mg (100 %) of 1-(pyrrolidin-1-yl)-2-(4-(thiophen-3-ylethynyl)phenoxy)ethan-1-one as a yellow solid (hexanes/EtOAc : 70/30).

^{1}H NMR (400 MHz, chloroform-*d*) δ 7.48 (dd, *J* = 3.0, 1.1 Hz, 1H), 7.46 – 7.42 (m, 2H), 7.29 (dd, *J* = 5.0, 3.0 Hz, 1H), 7.17 (dd, *J* = 5.0, 1.1 Hz, 1H), 6.95 – 6.89 (m, 2H), 4.64 (s, 2H), 3.52 (td, *J* = 6.8, 4.6 Hz, 4H), 1.97 (p, *J* = 7.1, 6.6 Hz, 2H), 1.86 (p, *J* = 6.5 Hz, 2H).

^{13}C NMR (101 MHz, chloroform-*d*) δ 166.30, 158.14, 133.19, 129.99, 128.31, 125.41, 122.62, 116.41, 114.86, 88.69, 83.57, 68.13, 46.39, 46.17, 26.41, 23.93.

HRMS(EI): Calcd. for $C_{18}H_{17}NO_2SNa^+$: 334.0878 Found: 334.0894.

5-Fluoro-2-(thiophen-3-ylethynyl)benzonitrile (13)

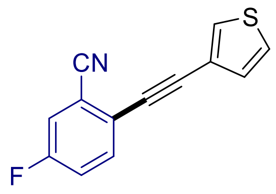

2-Bromo-5-fluorobenzonitrile (40 mg, 0.2 mmol), 3-ethynylthiophene (43.3 mg, 39 μL, 0.4 mmol), $Pd(OAc)_2$ (0.0225 mg, 0.0001 mmol), XPhos (2.9 mg, 0.006 mmol), Fe NPs (8 mg, 5% $FeCl_3$) and DIPEA (51.7 mg, 0.07 mL, 0.4 mmol) in 0.4 mL 2 wt % TPGS-750-M/H_2O were reacted at 45 °C for 24 h yielding 43 mg (95%) of 5-fluoro-2-(thiophen-3-ylethynyl)benzonitrile as a yellow oil (hexanes/EtOAc : 90/10).

^{1}H NMR (400 MHz, chloroform-*d*) δ 7.64 (dd, *J* = 3.0, 1.2 Hz, 1H), 7.59 (dd, *J* = 8.8, 5.2 Hz, 1H), 7.36 (dd, *J* = 7.9, 2.6 Hz, 1H), 7.33 (dd, *J* = 5.0, 3.0 Hz, 1H), 7.31 – 7.26 (m, 1H), 7.25 (dd, *J* = 5.0, 1.2 Hz, 1H).

^{13}C NMR (126 MHz, chloroform-*d*) δ 161.33 (d, $J_{(C-F)}$ = 253.3 Hz), 134.19 (d, $J_{(C-F)}$ = 8.8 Hz), 130.52, 129.95, 125.90, 123.89 (d, $J_{(C-F)}$ = 3.8 Hz), 121.06, 120.57 (d, $J_{(C-F)}$ = 21.4 Hz), 119.81 (d, $J_{(C-F)}$ = 25.2 Hz), 116.72 (d, $J_{(C-F)}$ = 10.1 Hz), 116.52 (d, $J_{(C-F)}$ = 2.5 Hz), 91.13, 84.30.

^{19}F NMR (376 MHz, chloroform-*d*) δ -108.80.

HRMS(EI): Calcd. for $C_{13}H_6FNSNa^+$: 250.0103 Found: 250.0110.

1-(*t*-Butyl) 2-methyl (2*S*,4*R*)-4-((4-((4-chlorophenyl)ethynyl)-7-fluoroisoindoline-2-carbonyl)oxy)pyrrolidine-1,2-dicarboxylate (14)

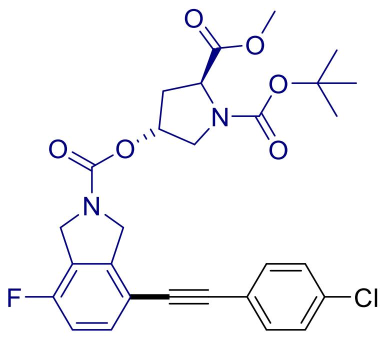

1-(*t*-Butyl)-2-methyl-(2*S*,4*R*)-4-((4-bromo-7-fluoroisoindoline-2-carbonyl)oxy)pyrrolidine-1,2-dicarboxylate (97.5 mg, 0.2 mmol), 1-chloro-4-ethynylbenzene (54.6 mg, 0.4 mmol), $Pd(OAc)_2$ (0.0338 mg, 0.00015 mmol), XPhos (2.9 mg, 0.006 mmol), Fe NPs (8 mg, 5% $FeCl_3$), and DIPEA (51.7 mg, 0.07 mL, 0.4 mmol) in 0.4 mL 2 wt % TPGS-750-M/H_2O were reacted at 55 °C for 48 h yielding 88 mg (81%) of 1-(*t*-butyl)-2-methyl-(2*S*,4*R*)-4-((4-((4-chlorophenyl)ethynyl)-7-fluoroisoindoline-2-carbonyl)oxy) pyrrolidine-1,2-dicarboxylate (hexanes/EtOAc : 75/25).

^{1}H NMR (400 MHz, chloroform-*d*) δ 7.50 (d, *J* = 3.6 Hz, 1H), 7.44 (m, 2H), 7.40 – 7.31 (m, 2H), 7.04 – 6.94 (m, 1H), 5.34 (s, 1H), 4.84 (d, *J* = 17.6 Hz, 2H), 4.76 (m, 2H), 4.44 (m, *J* = 35.7, 7.6 Hz, 1H), 3.76 (m, 5H), 2.56 – 2.41 (m, 1H), 2.25 (m, 1H), 1.44 (m, 9H).

^{13}C NMR (126 MHz, chloroform-*d*) δ 173.28, 173.00, 158.78, 158.62, 156.77, 156.62, 154.55, 154.52, 153.95, 153.92, 153.86, 142.39, 142.35, 134.93, 133.33, 133.28, 133.02, 132.98, 131.73, 128.96, 128.95, 123.99, 123.84, 121.23, 121.18, 115.17, 115.07, 115.01, 114.91, 92.35, 86.14, 80.76, 80.69, 74.13, 74.06, 73.39, 73.37, 58.21, 57.78, 53.57, 53.11, 52.85, 52.54, 52.34, 50.45, 50.01, 37.12, 36.12, 36.08, 28.51, 28.39.

^{19}F NMR (376 MHz, chloroform-*d*) δ -114.97, -115.07, -115.30, -115.51.

HRMS(EI): Calcd. for $C_{28}H_{28}ClFN_2O_6Na^+$: 565.1517 Found: 565.1520.

1-Methoxy-4-(*p*-tolylethynyl)benzene (15)

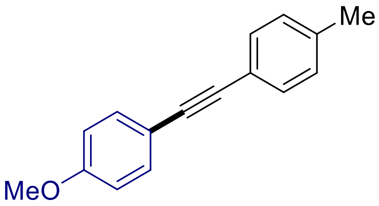

1-Bromoanisole (37.4 mg, 25 μL, 0.2 mmol), 1-ethynyl-4-methylbenzene (46.5 mg, 51 μL, 0.4 mmol), $Pd(OAc)_2$ (0.0225 mg, 0.0001 mmol), XPhos (2.9 mg, 0.006 mmol), Fe NPs (8 mg, 5% $FeCl_3$), and DIPEA (51.7 mg, 0.07 mL, 0.4 mmol) in 0.4 mL 2 wt % TPGS-750-M/H_2O were reacted at 45 °C for 24 h yielding 42.8 mg (96%) of 1-methoxy-4-(*p*-tolylethynyl)benzene as a yellow crystal (hexanes/EtOAc : 97/3).

^{1}H NMR (500 MHz, chloroform-*d*) δ 7.46 (d, *J* = 8.9 Hz, 2H), 7.40 (d, *J* = 8.1 Hz, 2H), 7.14 (d, *J* = 7.9 Hz, 2H), 6.87 (d, *J* = 8.8 Hz, 2H), 3.83 (s, 3H), 2.36 (s, 3H).

^{13}C NMR (126 MHz, chloroform-*d*) δ 159.63, 138.16, 133.12, 131.48, 129.22, 120.64, 115.75, 114.11, 88.79, 88.33, 55.45, 21.64.

Spectral data matched those previously reported.[6]

(*E*)-2-(4-(2-(Pyridin-2-yl)vinyl)phenoxy)-1-(pyrrolidin-1-yl)ethan-1-one (16)

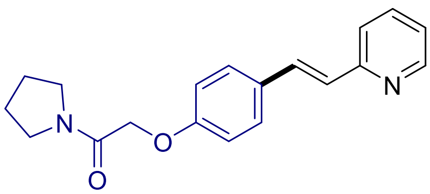

2-(4-Iodophenoxy)-1-(pyrrolidin-1-yl)ethan-1-one (66.2 mg, 0.2 mmol), 2-vinylpyridine (42 mg, 43 μL, 0.4 mmol), Pd(*t*$Bu_3P)_2$ (0.26 mg, 0.0005 mmol), *t*Bu_3P (1 mg, 0.005 mmol), Fe NPs (2.8 mg, 1.8% $FeCl_3$), K_3PO_4 (127.4 mg, 0.6 mmol), and NaCl (70.2 mg, 1.2 mmol) in 0.4 mL 2 wt % TPGS-750-M/H_2O with

0.04 mL DMF were reacted at 45 °C for 40 h yielding 61.7 mg (100%) of (*E*)-2-(4-(2-(pyridin-2-yl)vinyl)phenoxy)-1-(pyrrolidin-1-yl)ethan-1-one as a yellow solid (hexanes/EtOAc : 60/40).

^{1}H NMR (500 MHz, chloroform-*d*) δ 8.56 (d, *J* = 4.6 Hz, 1H), 7.64 – 7.58 (m, 1H), 7.55 (d, *J* = 16.1 Hz, 1H), 7.49 (d, *J* = 8.7 Hz, 2H), 7.33 (d, *J* = 7.9 Hz, 1H), 7.12 – 7.06 (m, 1H), 7.02 (d, *J* = 16.1 Hz, 1H), 6.93 (d, *J* = 8.6 Hz, 2H), 4.62 (s, 2H), 3.50 (q, *J* = 6.8 Hz, 4H), 1.94 (p, *J* = 6.8 Hz, 2H), 1.82 (p, *J* = 6.8 Hz, 2H).

^{13}C NMR (126 MHz, chloroform-*d*) δ 166.36, 158.35, 155.88, 149.64, 136.59, 132.14, 130.31, 128.55, 126.29, 121.92, 121.84, 114.97, 68.06, 46.28, 46.07, 26.31, 23.85.

HRMS(EI): Calcd. for $C_{19}H_{20}N_2O_2H^+$: 309.1603 Found: 309.1601.

(*E*)-1-(4-((3-Methylbut-2-en-1-yl)oxy)styryl)-3-nitrobenzene (17)

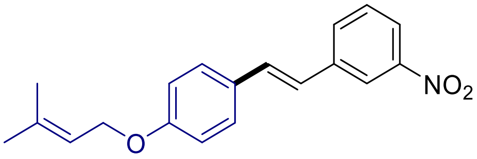

1-Iodo-4-((3-methylbut-2-en-1-yl)oxy)benzene (57.6 mg, 0.2 mmol), 1-nitro-3-vinylbenzene (59.7 mg, 56 μL, 0.4 mmol), Pd(*t*Bu$_3$P)$_2$ (0.26 mg, 0.0005 mmol), *t*Bu$_3$P (1 mg, 0.005 mmol), Fe NPs (2.8 mg, 1.8% $FeCl_3$), K_3PO_4 (127.4 mg, 0.6 mmol) and NaCl (70.2 mg, 1.2 mmol) in 0.4 mL 2 wt % TPGS-750-M/H_2O with 0.04 mL DMF were reacted at 45 °C for 40 h yielding 54.3 mg (88%) of (*E*)-1-(4-((3-methylbut-2-en-1-yl)oxy)styryl)-3-nitrobenzene as a yellow solid (hexanes/EtOAc : 90/10).

^{1}H NMR (500 MHz, chloroform-*d*) δ 8.33 (t, *J* = 1.8 Hz, 1H), 8.06 (ddd, *J* = 8.1, 2.1, 0.8 Hz, 1H), 7.76 (d, *J* = 7.8 Hz, 1H), 7.49 (m, 3H), 7.18 (d, *J* = 16.3 Hz, 1H), 6.99 (d, *J* = 16.3 Hz, 1H), 6.94 (d, *J* = 8.7 Hz, 2H), 5.51 (tt, *J* = 6.7, 1.3 Hz, 1H), 4.55 (d, *J* = 6.7 Hz, 2H), 1.81 (s, 3H), 1.76 (s, 3H).

^{13}C NMR (126 MHz, chloroform-*d*) δ 159.45, 148.87, 139.70, 138.64, 132.12, 131.47, 129.62, 129.07, 128.26, 123.96, 121.69, 120.70, 119.56, 115.15, 65.01, 25.98, 18.37.

HRMS(EI): Calcd. for $C_{19}H_{19}NO_3Na^+$: 332.1263 Found: 332.1259.

_t_-Butyl (_E_)-3-(4-methoxyphenyl)acrylate (18)

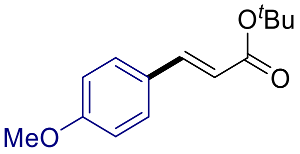

4-Iodoanisole (46.8 mg, 0.2 mmol), *t*-butyl acrylate (51.3 mg, 59 μL, 0.4 mmol), Pd(tBu$_3$P)$_2$ (0.26 mg, 0.0005 mmol), tBu$_3$P (1 mg, 0.005 mmol), Fe NPs (2.8 mg, 1.8% $FeCl_3$), K_3PO_4 (127.4 mg, 0.6 mmol), and NaCl (70.2 mg, 1.2 mmol) in 0.4 mL 2 wt % TPGS-750-M/H_2O with 0.04 mL DMF were reacted at 45 °C for 40 h yielding 46.9 mg (100%) of *t*-butyl (*E*)-3-(4-methoxyphenyl)acrylate as a white solid (hexanes/EtOAc : 90/10).

^{1}H NMR (400 MHz, chloroform-*d*) δ 7.54 (d, *J* = 15.9 Hz, 1H), 7.45 (d, *J* = 8.7 Hz, 2H), 6.89 (d, *J* = 8.8 Hz, 2H), 6.24 (d, *J* = 15.9 Hz, 1H), 3.83 (s, 3H), 1.53 (s, 9H).

^{13}C NMR (126 MHz, chloroform-*d*) δ 166.83, 161.26, 143.34, 129.69, 127.55, 117.86, 114.39, 80.36, 55.49, 28.38.

Spectral data matched those previously reported.[7]

2-Ethylhexyl (_E_)-3-(4-((2,5-dichloropyrimidin-4-yl)amino)phenyl)acrylate (19)

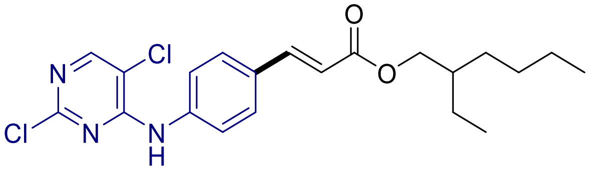

2,5-Dichloro-*N*-(4-iodophenyl)pyrimidin-4-amine (73.2 mg, 0.2 mmol), 2-ethylhexyl acrylate (73.7 mg, 83 μL, 0.4 mmol), Pd(tBu$_3$P)$_2$ (0.26 mg, 0.0005 mmol), tBu$_3$P (1 mg, 0.005 mmol), Fe NPs (2.8 mg, 1.8% $FeCl_3$), K_3PO_4 (127.4 mg, 0.6 mmol), and NaCl (70.2 mg, 1.2 mmol) in 0.4 mL 2 wt % TPGS-750-M/H_2O with 0.04 mL DMF were reacted at 45 °C for 40 h yielding 80.3 mg (95 %) of 2-ethylhexyl (*E*)-3-(4-((2,5-dichloropyrimidin-4-yl)amino)phenyl)acrylate as a yellow oil (hexanes/EtOAc : 50/50).

^{1}H NMR (400 MHz, chloroform-*d*) δ 8.22 (s, 1H), 7.72 – 7.59 (m, 3H), 7.55 (d, *J* = 8.6 Hz, 2H), 7.38 (s, 1H), 6.40 (d, *J* = 16.0 Hz, 1H), 4.22 – 4.03 (m, 2H), 1.65 (m, 1H), 1.47 – 1.27 (m, 8H), 0.91 (m, 6H).

^{13}C NMR (126 MHz, chloroform-*d*) δ 167.32, 158.30, 156.17, 155.04, 143.53, 138.71, 131.14, 129.15, 120.98, 117.94, 114.09, 67.14, 38.98, 30.58, 29.07, 23.97, 23.10, 14.19, 11.15.

HRMS(EI): Calcd. for $C_{21}H_{25}Cl_2N_3O_2Na^+$: 444.1222 Found: 444.1222.

(1*R*,4*S*)-1,7,7-Trimethylbicyclo[2.2.1]heptan-2-yl (*E*)-3-(4-(4-(2-(4-chlorophenoxy)acetyl)piperazin-1-yl)phenyl)acrylate (20)

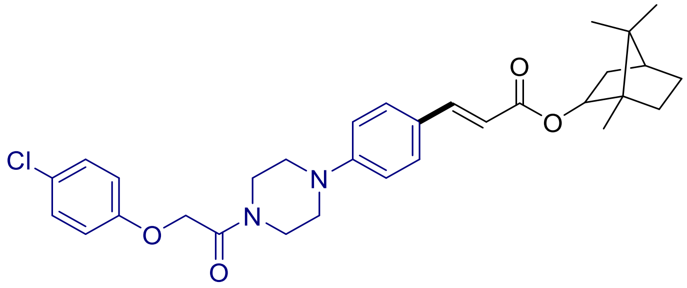

2-(4-Chlorophenoxy)-1-(4-(4-iodophenyl)piperazin-1-yl)ethan-1-one (91.3 mg, 0.2 mmol), (1*R*,4*S*)-1,7,7-trimethylbicyclo[2.2.1]heptan-2-yl acrylate (83.3 mg, 85 μL, 0.4 mmol), Pd(*t*Bu$_3$P)$_2$ (0.26 mg, 0.0005 mmol), *t*Bu$_3$P (1 mg, 0.005 mmol), Fe NPs (2.8 mg, 1.8% $FeCl_3$), K_3PO_4 (127.4 mg, 0.6 mmol) and NaCl (70.2 mg, 1.2 mmol) in 0.4 mL 2 wt % TPGS-750-M/H_2O with 0.04 mL DMF were reacted at 45 °C for 40 h yielding 106.3 mg (99%) of (1*R*,4*S*)-1,7,7-trimethylbicyclo[2.2.1]heptan-2-yl (*E*)-3-(4-(4-(2-(4-chlorophenoxy)acetyl)piperazin-1-yl)phenyl)acrylate as a yellow oil (hexanes/EtOAc : 60/40).

^{1}H NMR (500 MHz, chloroform-*d*) δ 7.55 (d, *J* = 15.9 Hz, 1H), 7.43 (d, *J* = 8.7 Hz, 2H), 7.24 (d, *J* = 8.9 Hz, 2H), 6.89 (d, *J* = 8.9 Hz, 2H), 6.85 (d, *J* = 8.7 Hz, 2H), 6.25 (d, *J* = 15.9 Hz, 1H), 4.78 (dd, *J* = 7.3, 4.1 Hz, 1H), 4.71 (s, 2H), 3.75 (d, *J* = 19.0 Hz, 4H), 3.33 – 3.18 (m, 4H), 1.89 – 1.66 (m, 4H), 1.57 (td, *J* = 12.2, 4.0 Hz, 1H), 1.27 – 1.03 (m, 5H), 0.87 (m, 6H).

^{13}C NMR (126 MHz, chloroform-*d*) δ 167.06, 166.30, 156.43, 151.86, 143.89, 129.68, 129.60, 126.86, 126.21, 116.00, 115.83, 115.62, 80.91, 68.02, 48.95, 48.67, 48.15, 47.06, 45.17, 45.08, 41.87, 38.98, 33.85, 27.17, 20.26, 20.12, 11.61.

HRMS(EI): Calcd. for $C_{31}H_{37}ClN_2O_4Na+$: 559.2339 Found: 559.2338.

4-(Benzo[b]thiophen-5-yl)butanenitrile (21)

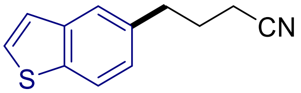

5-Bromobenzo[b]thiophene (42.6 mg, 0.2 mmol), 4-bromobutanenitrile (118.4 mg, 80 μL, 0.8 mmol), $Pd(OAc)_2$ (0.11 mg, 0.0005 mmol), AmPhos (2.7 mg, 0.01 mmol), Fe NPs (8 mg, 5% $FeCl_3$), Zn powder (52 mg, 0.8 mmol) and TMEDA (116.2 mg, 0.15 mL, 1.0 mmol) in 1.0 mL 2 wt % TPGS-750-M/H_2O were reacted at 45 °C for 40 h yielding 38.5 mg (96%) of 4-(benzo[b]thiophen-5-yl)butanenitrile as a yellow oil (hexanes/EtOAc : 90/10).

^1H NMR (400 MHz, chloroform-*d*) δ 7.82 (d, *J* = 8.3 Hz, 1H), 7.67 – 7.61 (m, 1H), 7.45 (d, *J* = 5.4 Hz, 1H), 7.30 (d, *J* = 5.4 Hz, 1H), 7.18 (dd, *J* = 8.3, 1.4 Hz, 1H), 2.91 (t, *J* = 7.4 Hz, 2H), 2.33 (t, *J* = 7.1 Hz, 2H), 2.04 (p, *J* = 7.2 Hz, 2H).

^{13}C NMR (126 MHz, chloroform-*d*) δ 140.12, 138.04, 135.86, 127.05, 125.09, 123.65, 123.31, 122.75, 119.63, 34.33, 27.25, 16.41.

HRMS(EI): Calcd. for $C_{12}H_{11}NSNa^+$: 224.0510 Found: 224.0510.

N-(4-Methyl-3-(oxetan-3-yl)phenyl)-3-(trifluoromethyl)benzamide (22)

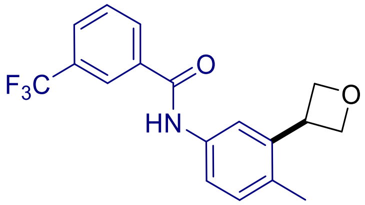

N-(3-Bromo-4-methylphenyl)-3-(trifluoromethyl)benzamide (71.6 mg, 0.2 mmol), 3-bromooxetane (109.6 mg, 62 μL, 0.8 mmol), $Pd(OAc)_2$ (0.11 mg, 0.0005 mmol), AmPhos (2.7 mg, 0.01 mmol), Fe NPs (8 mg, 5 % $FeCl_3$), Zn powder (52 mg, 0.8 mmol) and TMEDA (116.2 mg, 0.15 mL, 1.0 mmol) in 1.0 mL 2 wt % TPGS-750-M/H_2O were reacted at 45 °C for 40 h yielding 50.6 mg (76%) of *N*-(4-methyl-3-(oxetan-3-yl)phenyl)-3-(trifluoromethyl)benzamide as a white powder (hexanes/EtOAc : 70/30).

^{1}H NMR (400 MHz, DMSO-d_6) δ 10.43 (s, 1H), 8.35 – 8.24 (m, 2H), 7.96 (d, *J* = 7.7 Hz, 1H), 7.84 – 7.74 (m, 2H), 7.69 – 7.61 (m, 1H), 7.15 (d, *J* = 8.2 Hz, 1H), 4.96 (dd, *J* = 8.3, 5.7 Hz, 2H), 4.73 – 4.63 (m, 2H), 4.48 (q, *J* = 7.8 Hz, 1H), 2.09 (s, 3H).

^{13}C NMR (126 MHz, DMSO-d_6) δ 163.83, 139.61, 137.06, 135.82, 131.80, 131.08, 130.18, 129.73, 129.22 (q, $J_{(C-F)}$ = 32.8 Hz), 128.11 (q, $J_{(C-F)}$ = 3.8 Hz), 124.21 (q, $J_{(C-F)}$ = 3.8 Hz), 124.04 (q, $J_{(C-F)}$ = 273.4 Hz), 118.68, 117.83, 76.15, 36.70, 18.42.

^{19}F NMR (376 MHz, DMSO-*d*6) δ -61.09.

HRMS(EI): Calcd. for $C_{18}H_{16}F_3NO_2H^+$: 336.1211 Found: 336.1208.

4-(5-(4-Fluorophenethyl)pyrimidin-2-yl)morpholine (23)

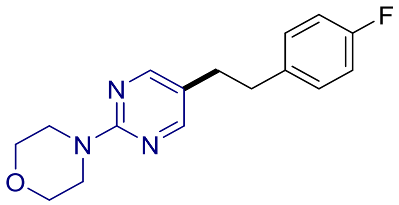

4-(5-Bromopyrimidin-2-yl)morpholine (48.8 mg, 0.2 mmol), 1-(2-bromoethyl)-4-fluorobenzene (162.4 mg, 0.11 mL, 0.8 mmol), $Pd(OAc)_2$ (0.11 mg, 0.0005 mmol), AmPhos (2.7 mg, 0.01 mmol), Fe NPs (8 mg, 5% $FeCl_3$), Zn powder (52 mg, 0.8 mmol), and TMEDA (116.2 mg, 0.15 mL, 1.0 mmol) in 1.0 mL 2 wt % TPGS-750-M/H_2O were reacted at 45 ° C for 40 h yielding 48.7 mg (76%) of 4-(5-(4-fluorophenethyl)pyrimidin-2-yl)morpholine as a yellow oil (hexanes/EtOAc : 70/30).

^{1}H NMR (400 MHz, chloroform-*d*) δ 8.07 (s, 2H), 7.08 (ddd, *J* = 8.3, 5.3, 2.5 Hz, 2H), 7.00 – 6.92 (m, 2H), 3.75 (m, 8H), 2.82 (t, *J* = 7.2 Hz, 2H), 2.72 (t, *J* = 7.2 Hz, 2H).

^{13}C NMR (126 MHz, chloroform-*d*) δ 162.56, 161.34 (d, $J_{(C-F)}$ = 55.4 Hz), 157.71, 136.37 (d, $J_{(C-F)}$ = 3.8 Hz), 130.05 (d, $J_{(C-F)}$ = 7.6 Hz), 122.42, 115.41 (d, $J_{(C-F)}$ = 21.4 Hz), 66.96, 44.53, 36.84, 31.61.

^{19}F NMR (376 MHz, chloroform-*d*) δ -117.04.

HRMS(EI): Calcd. for $C_{16}H_{18}FN_3OH^+$: 288.1512 Found: 288.1526.

***t*-Butyl 5-(4-ethoxy-4-oxobutyl)-1H-indole-1-carboxylate (24)**

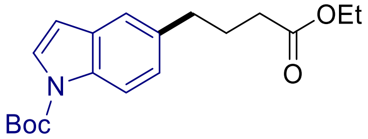

t-Butyl 5-bromo-1H-indole-1-carboxylate (59.2 mg, 0.2 mmol), ethyl 4-bromobutanoate (156 mg, 0.8 mmol), $Pd(OAc)_2$ (0.11 mg, 0.0005 mmol), AmPhos (2.7 mg, 0.01 mmol), Fe NPs (8 mg, 5% $FeCl_3$), Zn powder (52 mg, 0.8 mmol) and TMEDA (116.2 mg, 0.15 mL, 1.0 mmol) in 1.0 mL 2 wt % TPGS-750-M/H_2O were reacted at 45 °C for 40 h yielding 56.9 mg (86%) of *t*-butyl 5-(4-ethoxy-4-oxobutyl)-1H-indole-1-carboxylate as a yellow oil (hexanes/EtOAc : 90/10).

^{1}H NMR (600 MHz, chloroform-*d*) δ 8.14 – 7.91 (br, 1H), 7.60 – 7.50 (m, 1H), 7.37 – 7.34 (m, 1H), 7.13 (dd, *J* = 8.5, 1.6 Hz, 1H), 6.51 (d, *J* = 3.6 Hz, 1H), 4.12 (q, *J* = 7.1 Hz, 2H), 2.74 (t, *J* = 7.5 Hz, 2H), 2.32 (t, *J* = 7.5 Hz, 2H), 1.99 (p, *J* = 7.5 Hz, 2H), 1.67 (s, 9H), 1.25 (t, *J* = 7.1 Hz, 3H).

^{13}C NMR (126 MHz, chloroform-*d*) δ 173.77, 149.96, 135.91, 133.86, 130.93, 126.19, 125.12, 120.58, 115.10, 107.24, 83.67, 60.39, 35.15, 33.79, 28.35, 27.16, 14.40.

Spectral data matched those previously reported.[8]

1-(4-(3-Phenoxypropyl)phenyl)ethan-1-one (24)

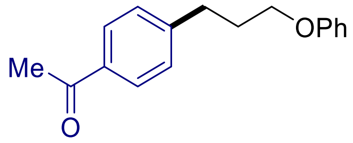

1-(4-Bromophenyl)ethan-1-one (40 mg, 0.2 mmol), (3-bromopropoxy)benzene (172 mg, 0.13 mL, 0.8 mmol), $Pd(OAc)_2$ (0.11 mg, 0.0005 mmol), AmPhos (2.7 mg, 0.01 mmol), Fe NPs (8 mg, 5% $FeCl_3$), Zn powder (52 mg, 0.8 mmol), and TMEDA (116.2 mg, 0.15 mL, 1.0 mmol) in 1.0 mL 2 wt % TPGS-750-M/H_2O were reacted at 45 °C for 40 h yielding 41.1 mg (81%) of 1-(4-(3-phenoxypropyl)phenyl)ethan-1-one as a yellow oil (hexanes/EtOAc : 90/10).

^{1}H NMR (500 MHz, chloroform-*d*) δ 7.89 (d, *J* = 8.2 Hz, 2H), 7.33 – 7.26 (m, 4H), 6.95 (t, *J* = 7.3 Hz, 1H), 6.89 (d, *J* = 7.8 Hz, 2H), 3.96 (t, *J* = 6.2 Hz, 2H), 2.92 – 2.84 (m, 2H), 2.59 (s, 3H), 2.13 (m, 2H).

^{13}C NMR (126 MHz, chloroform-*d*) δ 198.00, 159.02, 147.55, 135.35, 129.61, 128.90, 128.76, 120.87, 114.63, 66.63, 32.38, 30.66, 26.72.

Spectral data matched those previously reported.[8]

4-(Benzo[*b*]thiophen-5-yl)tetrahydro-2*H*-pyran (26)

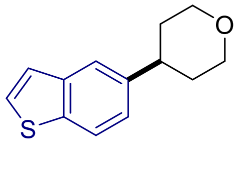

5-Bromobenzo[b]thiophene (42.6 mg, 0.2 mmol), 4-bromotetrahydro-2*H*-pyran (132 mg, 0.06 mL, 0.8 mmol), $Pd(OAc)_2$ (0.11 mg, 0.0005 mmol), AmPhos (2.7 mg, 0.01 mmol), Fe NPs (8 mg, 5% $FeCl_3$), Zn powder (52 mg, 0.8 mmol) and TMEDA (116.2 mg, 0.15 mL, 1.0 mmol) in 1.0 mL 2 wt % TPGS-750-M/H_2O were reacted at 45 °C for 40 h yielding 36.1 mg (83%) of 4-(benzo[*b*]thiophen-5-yl)tetrahydro-2*H*-pyran as a yellow oil (hexanes/EtOAc : 90/10).

^{1}H NMR (400 MHz, chloroform-*d*) δ 7.83 (d, *J* = 8.3 Hz, 1H), 7.68 (s, 1H), 7.44 (d, *J* = 5.4 Hz, 1H), 7.31 (d, *J* = 5.4 Hz, 1H), 7.24 (dd, *J* = 8.4, 1.3 Hz, 1H), 4.12 (dd, *J* = 11.1, 3.7 Hz, 2H), 3.57 (td, *J* = 11.6, 2.3 Hz, 2H), 2.88 (tt, *J* = 11.6, 4.1 Hz, 1H), 1.97 – 1.79 (m, 4H).

^{13}C NMR (126 MHz, chloroform-*d*) δ 142.28, 140.09, 137.85, 126.83, 123.89, 123.85, 122.58, 121.36, 68.58, 41.68, 34.41.

HRMS(EI): Calcd. for $C_{13}H_{14}OSH^{+}$: 219.0844 Found: 219.0853.

1-Methoxy-4-(phenylethynyl)benzene (27)

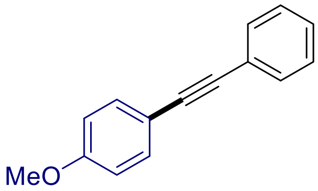

1-Bromoanisole (37.4 mg, 25 μL, 0.2 mmol), ethynylbenzene (40.9 mg, 44 μL, 0.4 mmol), $Pd(OAc)_2$ (0.0225 mg, 0.0001 mmol), XPhos (2.9 mg, 0.006 mmol), Fe NPs (8 mg, 5% $FeCl_3$) and DIPEA (51.7 mg, 0.07 mL, 0.4 mmol) in 0.4 mL 2 wt % TPGS-750-M/H_2O were reacted at 45 °C for 24 h yielding 39.6 mg (95%) of 1-methoxy-4-(phenylethynyl)benzene as a yellow crystal (hexanes/EtOAc : 97/3).

^{1}H NMR (400 MHz, chloroform-*d*) δ 7.57 – 7.45 (m, 4H), 7.39 – 7.29 (m, 3H), 6.96 – 6.84 (m, 2H), 3.83 (s, 3H).

^{13}C NMR (126 MHz, chloroform-*d*) δ 159.76, 133.19, 131.59, 128.44, 128.07, 123.74, 115.52, 114.14, 89.51, 88.20, 55.44.

Spectral data matched those previously reported.[9]

1-(4-Methoxyphenyl)naphthalene (29)

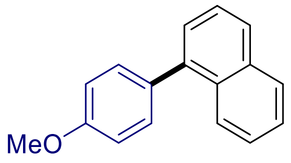

Followed the procedure for a gram scale reaction.

^{1}H NMR (400 MHz, chloroform-*d*) δ 7.98 – 7.89 (m, 2H), 7.85 (d, *J* = 8.2 Hz, 1H), 7.56 – 7.47 (m, 2H), 7.44 (m, 4H), 7.09 – 7.02 (m, 2H), 3.91 (s, 3H).

^{13}C NMR (101 MHz, chloroform-*d*) δ 159.09, 140.05, 133.98, 133.27, 131.97, 131.25, 128.40, 127.47, 127.05, 126.21, 126.06, 125.84, 125.54, 113.86, 55.50.

Spectral data matched those previously reported.[10]

Pyrrolidin-1-yl(4-(thiophen-3-yl)phenyl)methanone (32)

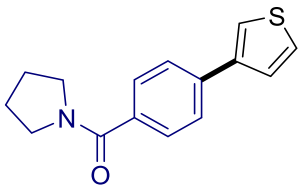

(4-Bromophenyl)(pyrrolidin-1-yl)methanone (50.8 mg, 0.2 mmol), thiophen-3-ylboronic acid (50.4 mg, 0.3 mmol), $Pd(OAc)_2$ (0.0225 mg, 0.0001 mmol), L1 ($FcPAd_2$) (4.9 mg, 0.01 mmol), Fe NPs (8 mg, 5% $FeCl_3$) and $K_3PO_4{\cdot}H_2O$ (69.2 mg, 0.3 mmol) in 0.4 mL 2 wt % TPGS-750-M/H_2O were reacted at 45 °C for 24 h yielding 48.8 mg (95%) of pyrrolidin-1-yl(4-(thiophen-3-yl)phenyl)methanone as a white solid (hexanes/EtOAc : 80/20).

^{1}H NMR (400 MHz, chloroform-*d*) δ 7.65 – 7.53 (m, 4H), 7.49 (t, *J* = 2.0 Hz, 1H), 7.39 (d, *J* = 2.0 Hz, 2H), 3.65 (t, *J* = 6.8 Hz, 2H), 3.47 (t, *J* = 6.4 Hz, 2H), 1.91 (ddt, *J* = 34.2, 13.0, 6.5 Hz, 4H).

^{13}C NMR (126 MHz, chloroform-*d*) δ 169.48, 141.56, 137.27, 135.79, 127.92, 126.60, 126.26, 126.21, 121.15, 49.74, 46.35, 26.53, 24.53.

HRMS(EI): Calcd. for $C_{15}H_{15}NOSNa^+$: 280.0772 Found: 280.0769.

2-Fluoro-4-(6-formylbenzo[d][1,3]dioxol-5-yl)benzonitrile (33)

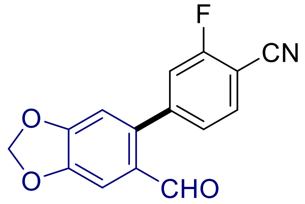

6-Bromobenzo[d][1,3]dioxole-5-carbaldehyde (45.8 mg, 0.2 mmol), (4-cyano-3-fluorophenyl)boronic acid (49.5 mg, 0.3 mmol), $Pd(OAc)_2$ (0.0225 mg, 0.0001 mmol), L1 ($FcPAd_2$) (4.9 mg, 0.01 mmol), Fe NPs (8 mg, 5% $FeCl_3$) and $K_3PO_4{\bullet}H_2O$ (69.2 mg, 0.3 mmol) in 0.4 mL 2 wt % TPGS-750-M/H_2O were reacted at 45 °C for 24 h yielding 27.4 mg (51%) of 2-fluoro-4-(6-formylbenzo[d][1,3]dioxol-5-yl)benzonitrile as a yellow oil (hexanes/EtOAc : 90/10).

^{1}H NMR (400 MHz, chloroform-*d*) δ 9.72 (s, 1H), 7.71 (t, J = 7.3 Hz, 1H), 7.49 (s, 1H), 7.26 – 7.21 (m, 2H), 6.80 (s, 1H), 6.14 (s, 2H).

^{13}C NMR (126 MHz, chloroform-*d*) δ 188.99, 162.89 (d, $J_{(C\text{-}F)}$ = 261.2 Hz), 152.59, 149.08, 145.37 (d, $J_{(C\text{-}F)}$ = 8.1 Hz), 139.65 (d, $J_{(C\text{-}F)}$ = 1.8 Hz), 133.47, 128.97, 126.80 (d, $J_{(C\text{-}F)}$ = 3.4 Hz), 118.16 (d, $J_{(C\text{-}F)}$ = 20.1 Hz), 113.65, 109.95, 107.36, 102.75, 101.39 (d, $J_{(C\text{-}F)}$ = 15.5 Hz).

^{19}F NMR (376 MHz, chloroform-*d*) δ -105.49.

HRMS(EI): Calcd. for $C_{15}H_8FNO_3H^+$: 270.0566 Found: 270.0561.

(Tetrahydrofuran-2-yl)methyl (*E*)-3-(4-(2-oxo-2-(pyrrolidin-1-yl)ethoxy)phenyl)acrylate (34)

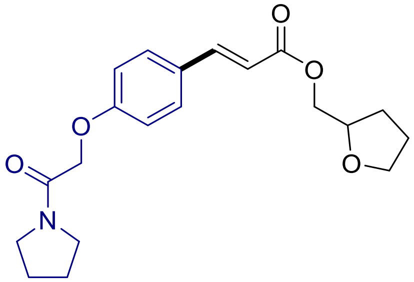

2-(4-Iodophenoxy)-1-(pyrrolidin-1-yl)ethan-1-one (66.2 mg, 0.2 mmol), (tetrahydrofuran-2-yl)methyl acrylate (62.5 mg, 59 μL, 0.4 mmol), Pd(tBu$_3$P)$_2$ (0.26 mg, 0.0005 mmol), L2 (FcPAd$_2$PtBu$_2$) (4.0 mg, 0.005 mmol), Fe NPs (2.8 mg, 1.8% $FeCl_3$), K_3PO_4 (127.4 mg, 0.6 mmol) and NaCl (70.2 mg, 1.2 mmol) in 0.4 mL 2 wt % TPGS-750-M/H_2O with 0.04 mL DMF were reacted at 45 °C for 40 h yielding 63.9 mg (89%) of (tetrahydrofuran-2-yl)methyl (*E*)-3-(4-(2-oxo-2-(pyrrolidin-1-yl)ethoxy)phenyl)acrylate as a yellow oil (hexanes/EtOAc : 80/20).

^{1}H NMR (400 MHz, chloroform-*d*) δ 7.63 (d, *J* = 16.0 Hz, 1H), 7.44 (d, *J* = 8.7 Hz, 2H), 6.93 (d, *J* = 8.7 Hz, 2H), 6.34 (d, *J* = 16.0 Hz, 1H), 4.63 (s, 2H), 4.27 (dd, *J* = 11.2, 3.2 Hz, 1H), 4.17 (qd, *J* = 6.9, 3.3 Hz, 1H), 4.09 (dd, *J* = 11.1, 6.9 Hz, 1H), 3.90 (q, *J* = 6.8 Hz, 1H), 3.80 (q, *J* = 7.9, 7.5 Hz, 1H), 3.49 (q, *J* = 6.6 Hz, 4H), 1.92 (m, 7H), 1.63 (dq, *J* = 11.9, 7.2 Hz, 1H).

^{13}C NMR (126 MHz, chloroform-*d*) δ 167.26, 166.08, 159.89, 144.61, 129.90, 128.03, 115.90, 115.12, 76.76, 68.58, 67.93, 66.60, 46.32, 46.07, 28.12, 26.35, 25.78, 23.89.

HRMS(EI): Calcd. for $C_{20}H_{25}NO_5H^+$: 360.1811 Found: 360.1797.

(*E*)-3-(4-((2,5-Dichloropyrimidin-4-yl)amino)phenyl)-1-morpholinoprop-2-en-1-one (35)

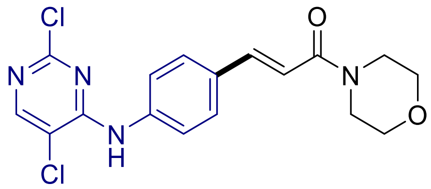

2,5-Dichloro-*N*-(4-iodophenyl)pyrimidin-4-amine (73.2 mg, 0.2 mmol), 1-morpholinoprop-2-en-1-one (56.5 mg, 0.4 mmol), Pd(tBu$_3$P)$_2$ (0.26 mg, 0.0005 mmol), L2 (FcPAd$_2$PtBu$_2$) (4.0 mg, 0.005 mmol), Fe NPs (2.8 mg, 1.8% $FeCl_3$), K_3PO_4 (127.4 mg, 0.6 mmol) and NaCl (70.2 mg, 1.2 mmol) in 0.4 mL 2 wt % TPGS-750-M/H_2O with 0.04 mL DMF were reacted at 45 °C for 40 h yielding 69.5 mg (92%) of (*E*)-3-(4-((2,5-dichloropyrimidin-4-yl)amino)phenyl)-1-morpholinoprop-2-en-1-one as a yellow oil (hexanes/ EtOAc : 50/50).

^{1}H NMR (500 MHz, chloroform-*d*) δ 8.23 (s, 1H), 7.70 – 7.65 (m, 3H), 7.56 (d, *J* = 8.5 Hz, 2H), 7.37 (s, 1H), 6.81 (d, *J* = 15.4 Hz, 1H), 3.70 (m, 8H).

^{13}C NMR (126 MHz, chloroform-*d*) δ 165.66, 158.32, 156.24, 155.04, 142.38, 138.30, 131.88, 128.88, 121.08, 116.15, 114.08, 66.99, 46.77, 41.71, 29.82.

Spectral data matched those previously reported.[11]

(*E*)-6-Chloro-*N*^{2}-(3-(4-(1-methyl-1*H*-indol-5-yl)styryl)phenyl)-1,3,5-triazine-2,4-diamine (44)

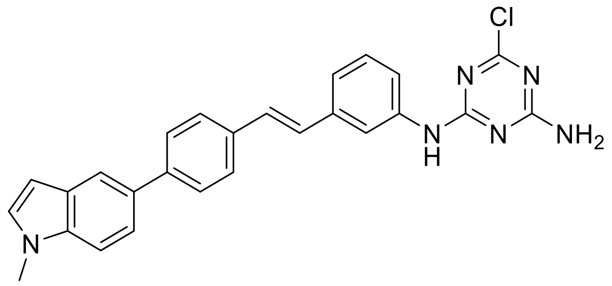

Followed the procedure for the one-pot sequence reaction.

^{1}H NMR (400 MHz, DMSO-d_6) δ 9.98 (s, 1H), 8.00 (s, 1H), 7.91 – 7.84 (m, 1H), 7.75 – 7.59 (m, 6H), 7.59 – 7.54 (m, 1H), 7.51 (s, 2H), 7.36 (d, *J* = 3.0 Hz, 1H), 7.32 (m, 2H), 7.25 (s, 2H), 6.49 (d, *J* = 3.1 Hz, 1H), 3.82 (s, 3H).

^{13}C NMR (126 MHz, DMSO-d_6) δ 168.42, 166.96, 164.05, 140.92, 139.36, 137.58, 136.10, 134.95, 130.84, 130.44, 128.90, 128.65, 128.32, 127.96, 127.11, 126.83, 121.12, 120.21, 119.70, 118.43, 118.25, 110.17, 100.88, 32.59.

HRMS(EI): Calcd. for $C_{26}H_{21}ClN_6H^+$: 453.1595 Found: 453.1616

References

(1) Handa, S.; Wang, Y.; Gallou, F.; Lipshutz B. H. Sustainable Fe–ppm Pd nanoparticle catalysis of Suzuki-Miyaura cross-couplings in water. *Science* **2015**, *349*, 1087-1091.

(2) Lipshutz, B. H.; Ghorai, S.; Abela, A. R.; Moser, R.; Nishikata, T.; Duplais, C.; Krasovskiy, A. *J. Org. Chem.* **2011**, *76*, 4379–4391.

(3) Khanapure, S. P.; Garvey, D. S.; Young, D. V.; Ezawa, M.; Earl, R. A.; Gaston, R. D.; Fang, X.; Murty, M.; Martino, A.; Shumway, M.; Trocha, M.; Marek, P.; Tam, S. W.; Janero, D. R.; Letts, L. G., Synthesis and Structure–Activity Relationship of Novel, Highly Potent Metharyl and Methcycloalkyl Cyclooxygenase-2 (COX-2) Selective Inhibitors. *J. Med. Chem.* **2003**, *46*, 5484-5504.

(4) Chakrabarty, I.; Akram, M. O.; Biswas, S.; Patil, N. T., Visible light mediated desilylative C(sp^2)–C(sp^2) cross-coupling reactions of arylsilanes with aryldiazonium salts under Au(i)/Au(iii) catalysis. *Chem. Commun.* **2018**, *54*, 7223-7226.

(5) Ge, S.; Hartwig, J. F., Highly Reactive, Single-Component Nickel Catalyst Precursor for Suzuki–Miyuara Cross-Coupling of Heteroaryl Boronic Acids with Heteroaryl Halides. *Angew. Chem., Int. Ed.* **2012**, *51*, 12837-12841.

(6) Fu, H.-Y.; Xu, N.; Pan, Y.-M.; Lu, X.-L.; Xia, M., Emission behaviours of novel V- and X-shaped fluorophores in response to pH and force stimuli. *Phys. Chem. Chem. Phys.* **2017**, *19*, 11563-11570.

(7) Mandali, P. K.; Chand, D. K., Palladium nanoparticles catalyzed Sonogashira reactions for the one-pot synthesis of symmetrical and unsymmetrical diarylacetylenes. *Catal. Commun.* **2014**, *47*, 40-44.

(8) Lauer, M. G.; Thompson, M. K.; Shaughnessy, K. H., Controlling Olefin Isomerization in the Heck Reaction with Neopentyl Phosphine Ligands. *J. Org. Chem.* **2014**, *79*, 10837-10848.

(9) Hu, Y.; Wong, M. J.; Lipshutz, B. H. ppm Pd-Containing Nanoparticles as Catalysts for Negishi Couplings … in Water. *Angew. Chem., Int. Ed.* **2022**, *61*, e202209784.

(10) Nie, X.; Liu, S.; Zong, Y.; Sun, P.; Bao, J., Facile synthesis of substituted alkynes by nano-palladium catalyzed oxidative cross-coupling reaction of arylboronic acids with terminal alkynes. *J. Organomet. Chem.* **2011**, *696*, 1570-1573.

(11) Tang, Y.-Q.; Lu, J.-M.; Shao, L.-X., NHC–Pd(II)–Im (NHC = N-heterocyclic carbene; Im = 1-methylimidazole) complexes as efficient catalysts for Suzuki-Miyaura coupling reactions of aryl chlorides. *J. Organomet. Chem.* **2011**, *696*, 3741-3744.

(12) Pang, H.; Hu, Y.; Yu, J.; Gallou, F.; Lipshutz, B. H. Water-Sculpting of a Heterogeneous Nanoparticle Precatalyst for Mizoroki–Heck Couplings under Aqueous Micellar Catalysis Conditions. *J. Am. Chem. Soc.* **2021**, *143*, 3373-3382.

^{1}H NMR, ^{13}C NMR and ^{19}F NMR spectra

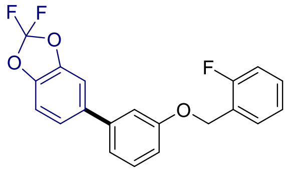

4, ^{1}H NMR

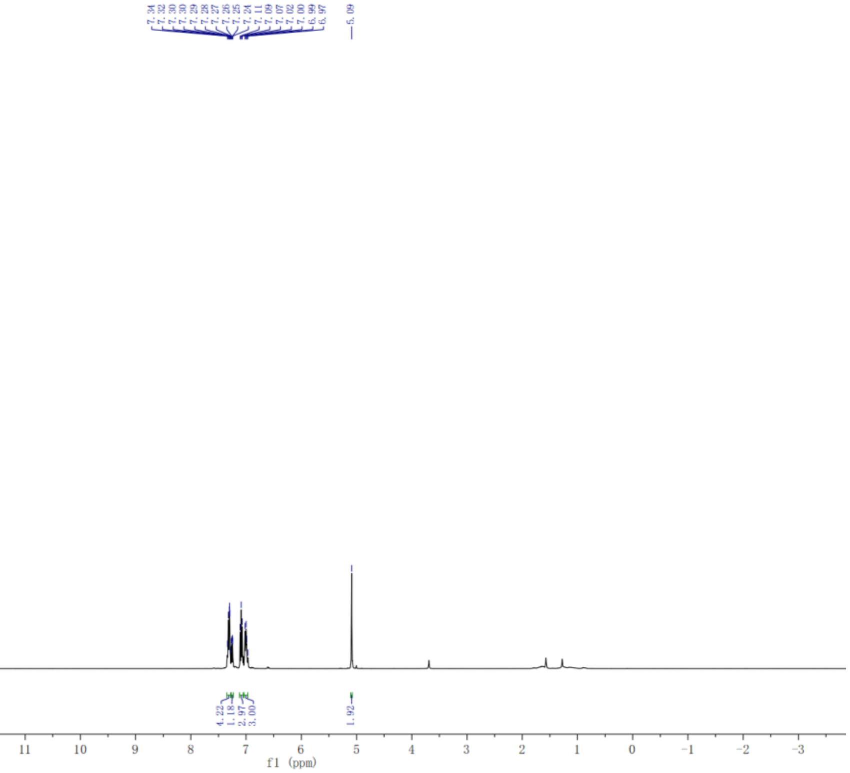

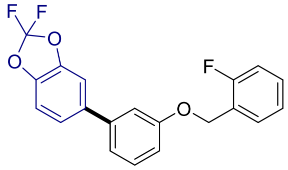

4, ^{13}C NMR

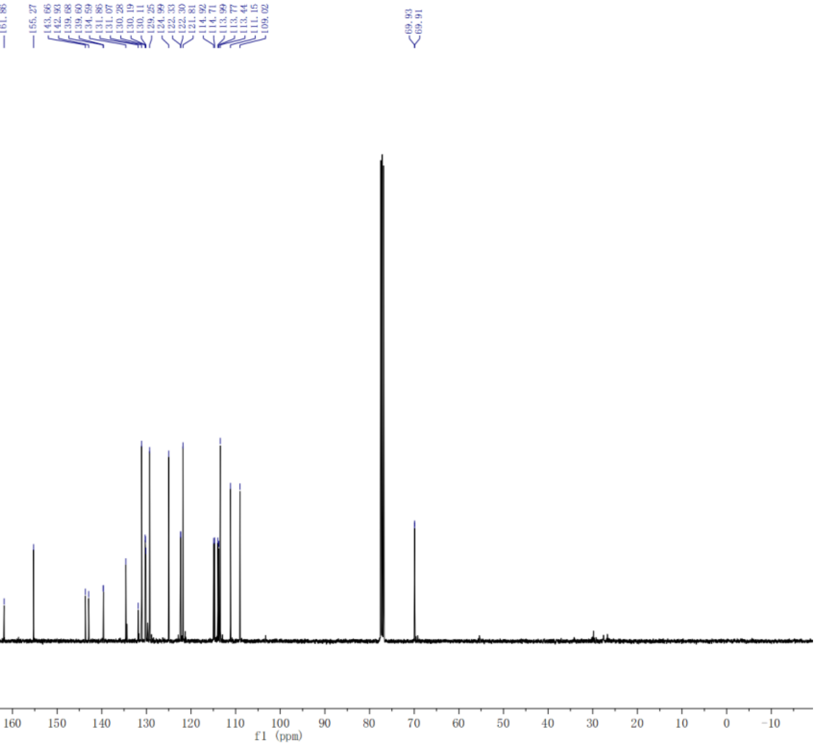

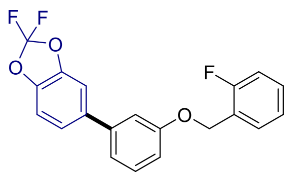

4, ^{19}F NMR

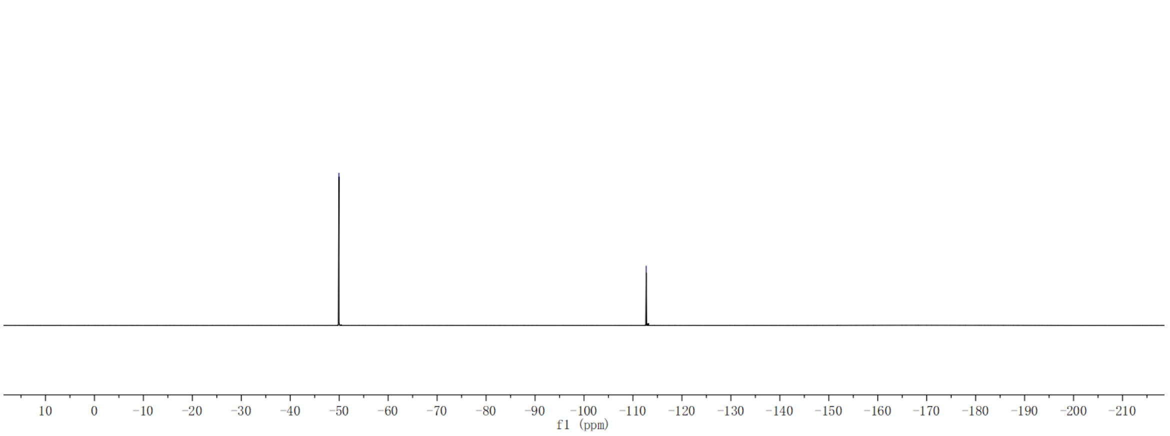
-49.94
-112.70
10 0 -10 -20 -30 -40 -50 -60 -70 -80 -90 -100 -110 -120 -130 -140 -150 -160 -170 -180 -190 -200 -210
f1 (ppm)

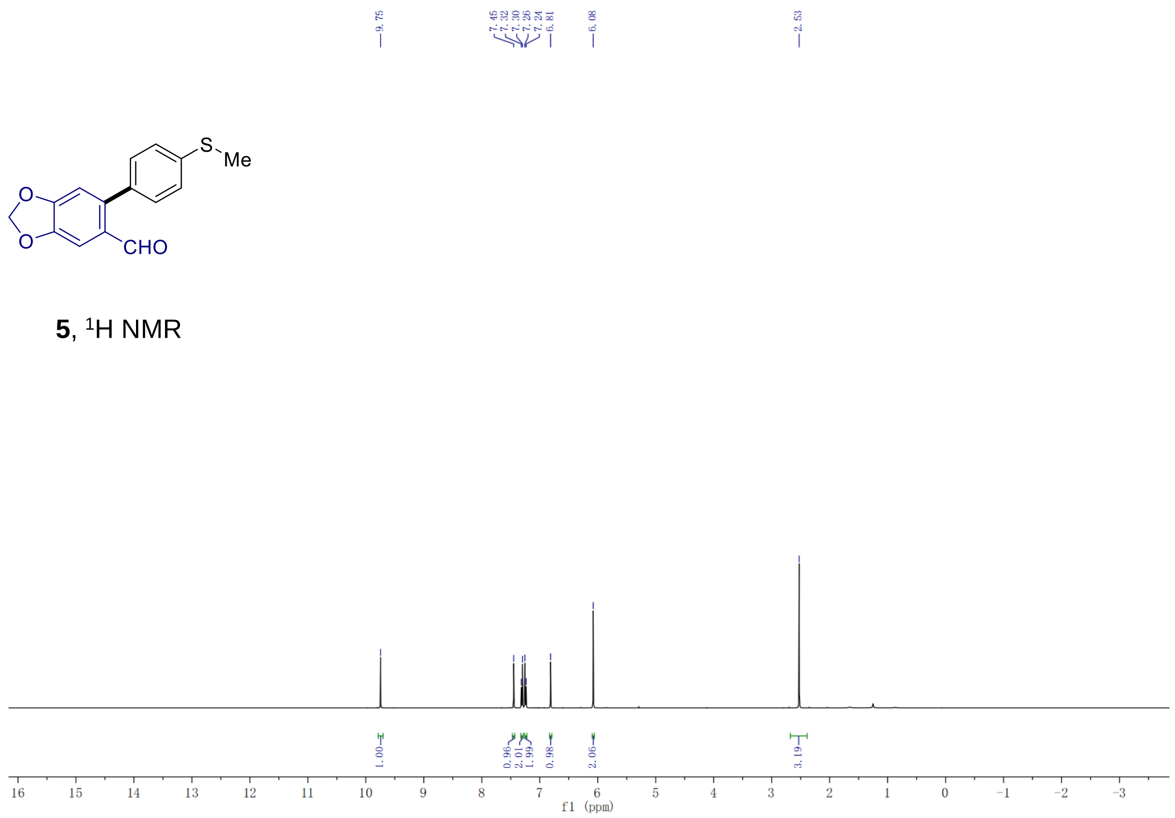

5, ^{1}H NMR

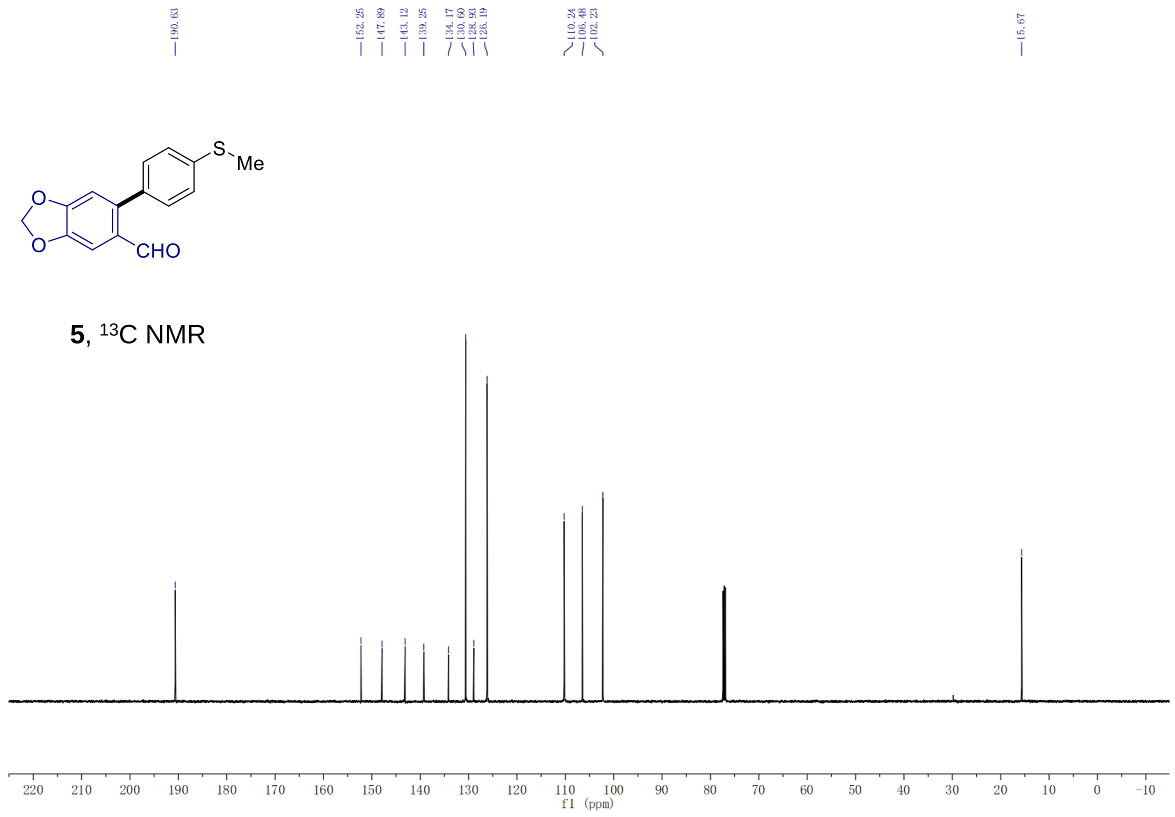

5, ^{13}C NMR

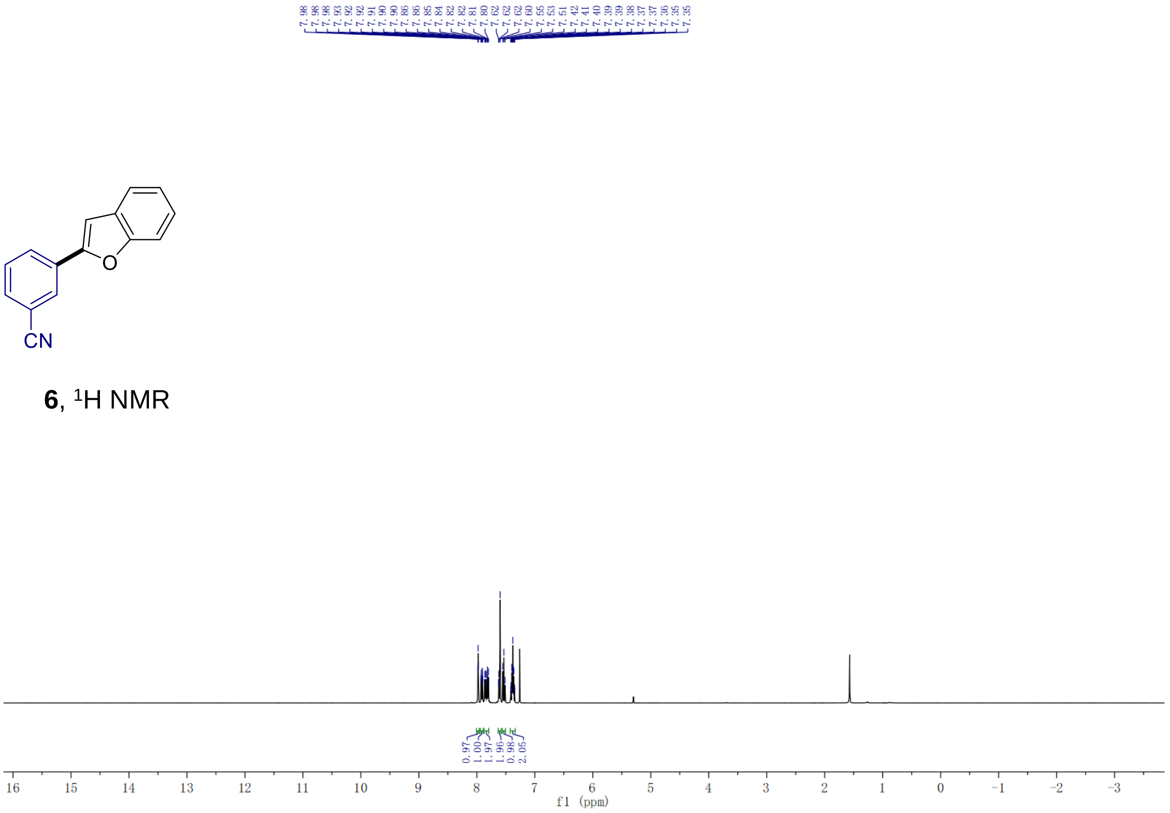

6, ^{1}H NMR

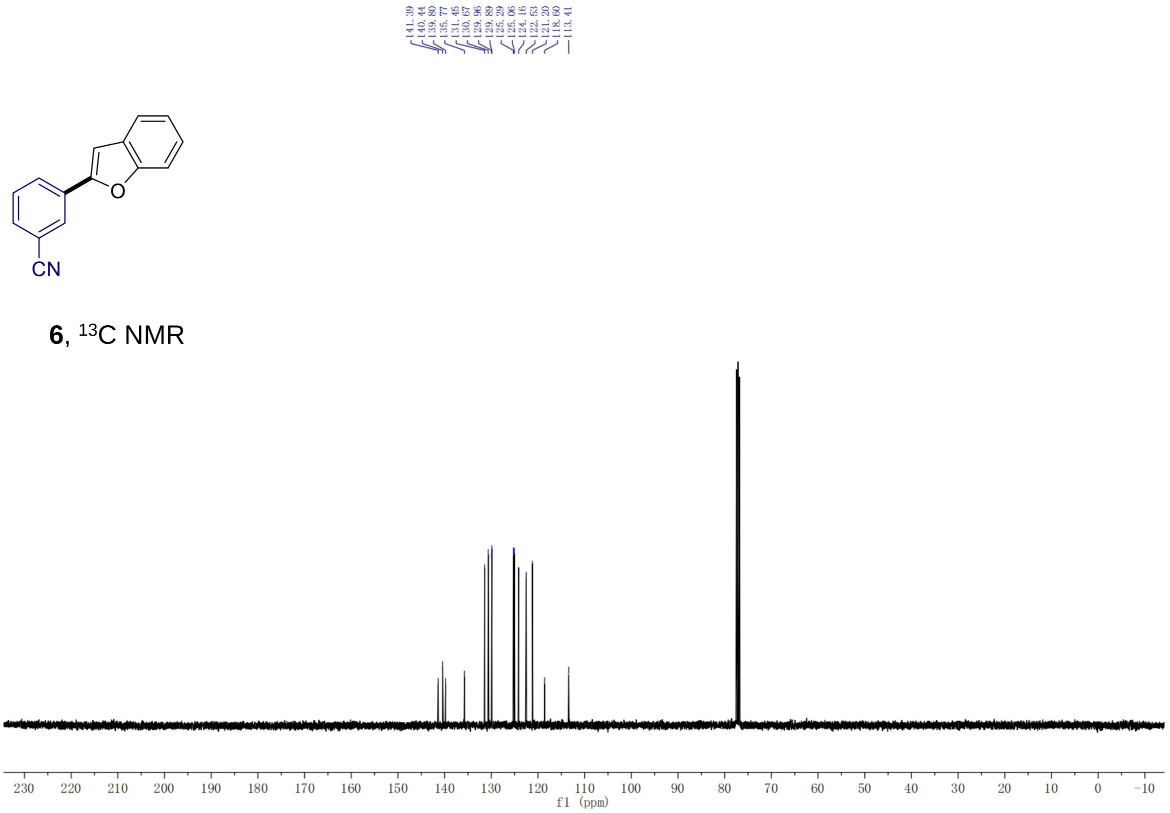

6, ^{13}C NMR

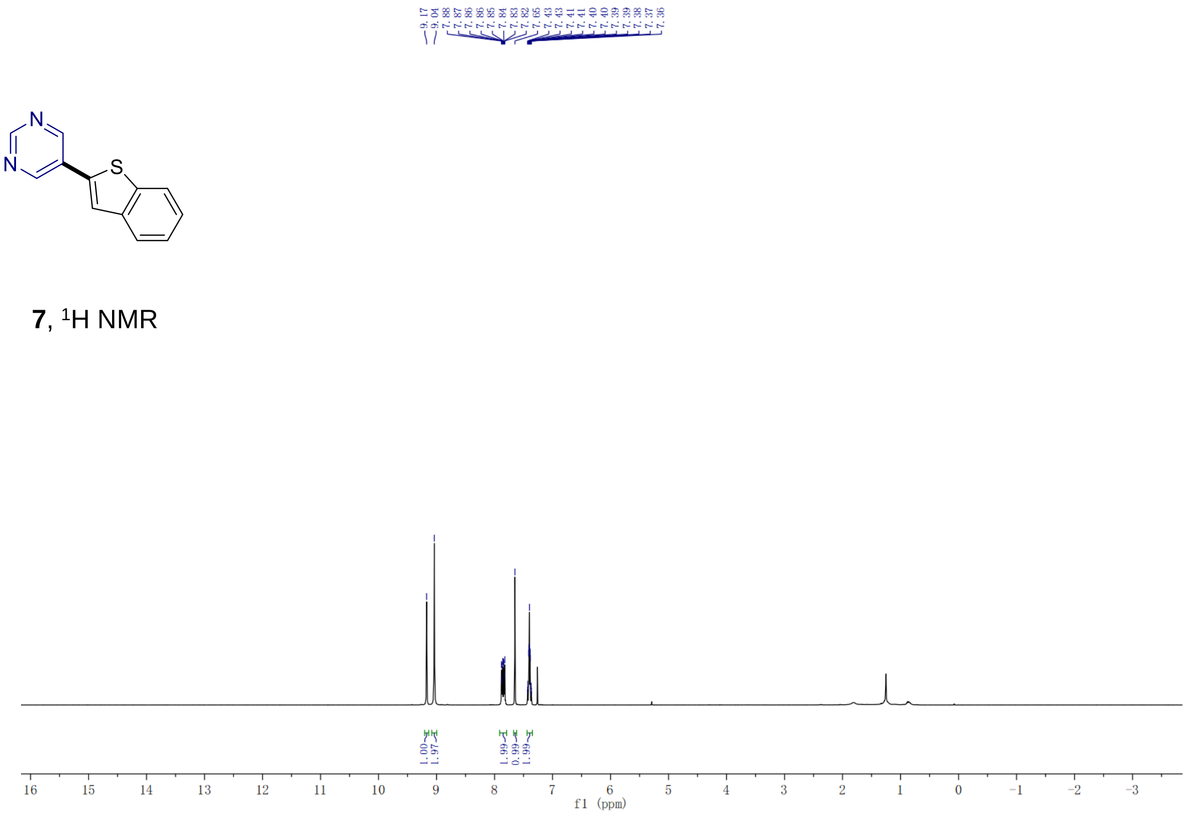

7, ^{1}H NMR

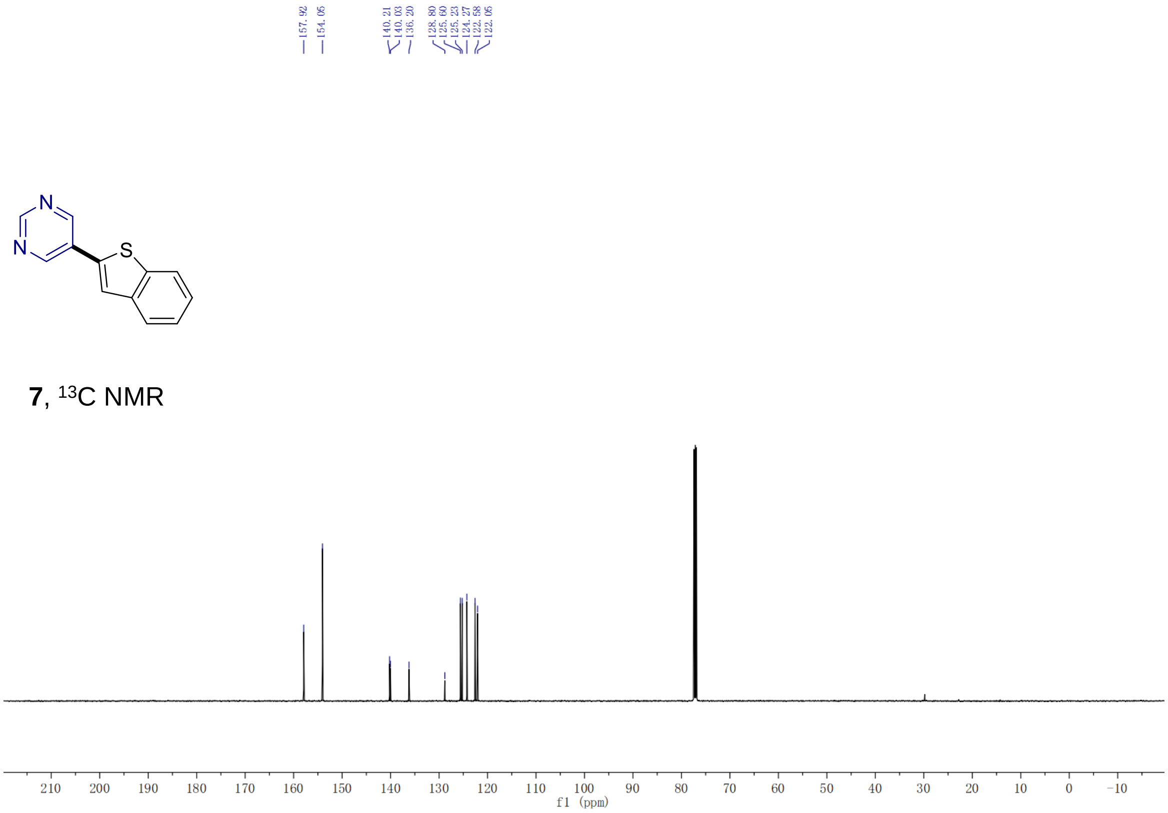

7, ^{13}C NMR

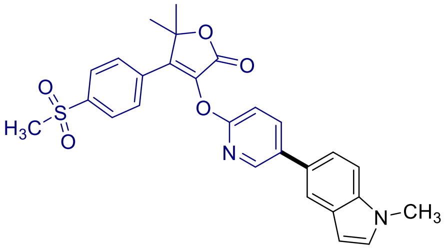

8, ^{1}H NMR

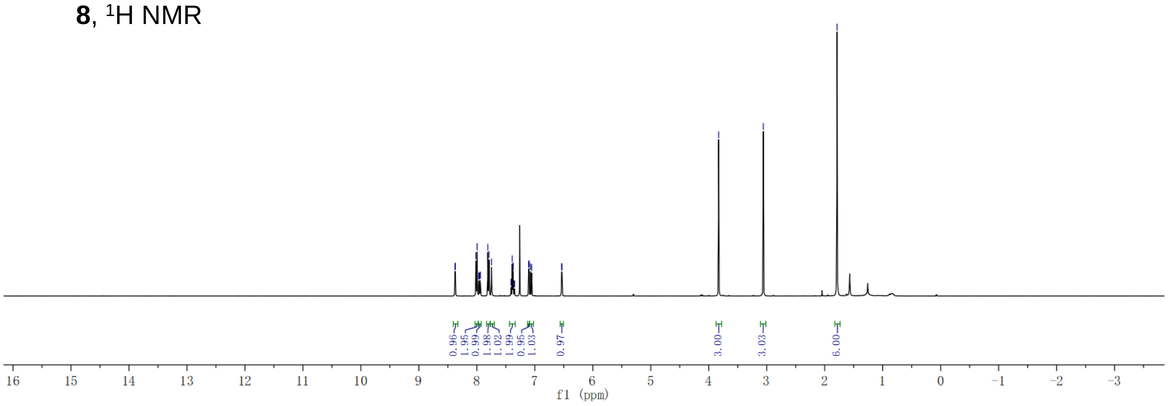

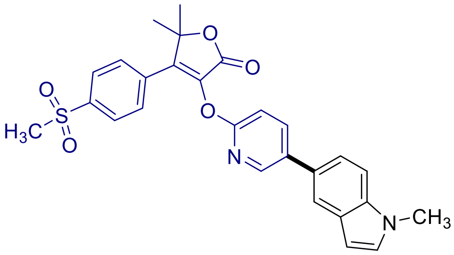

8, ^{13}C NMR

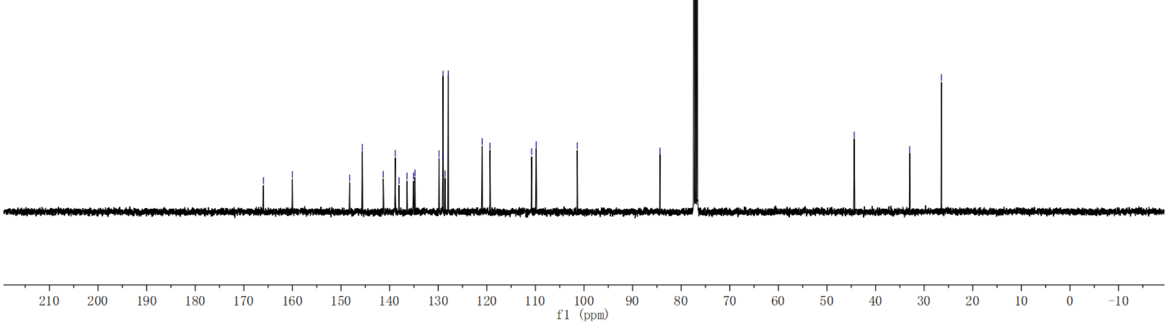

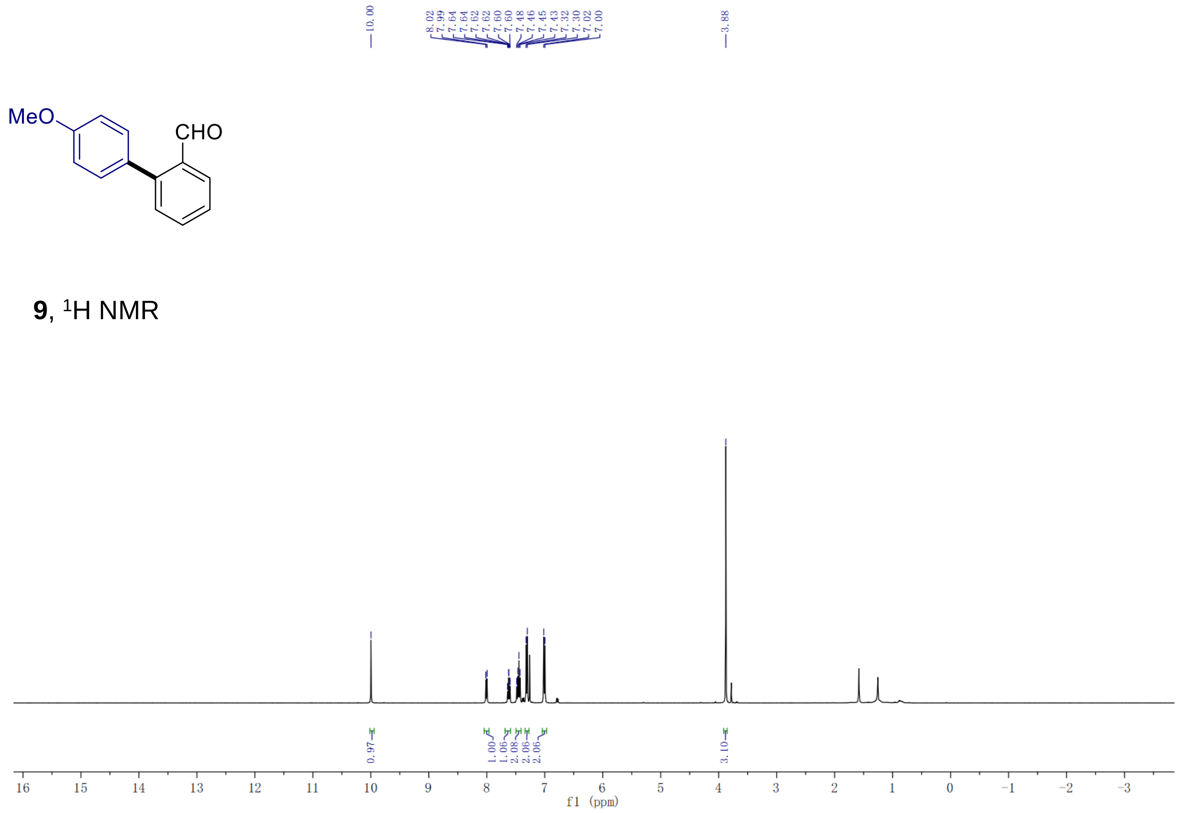

9, ^{1}H NMR

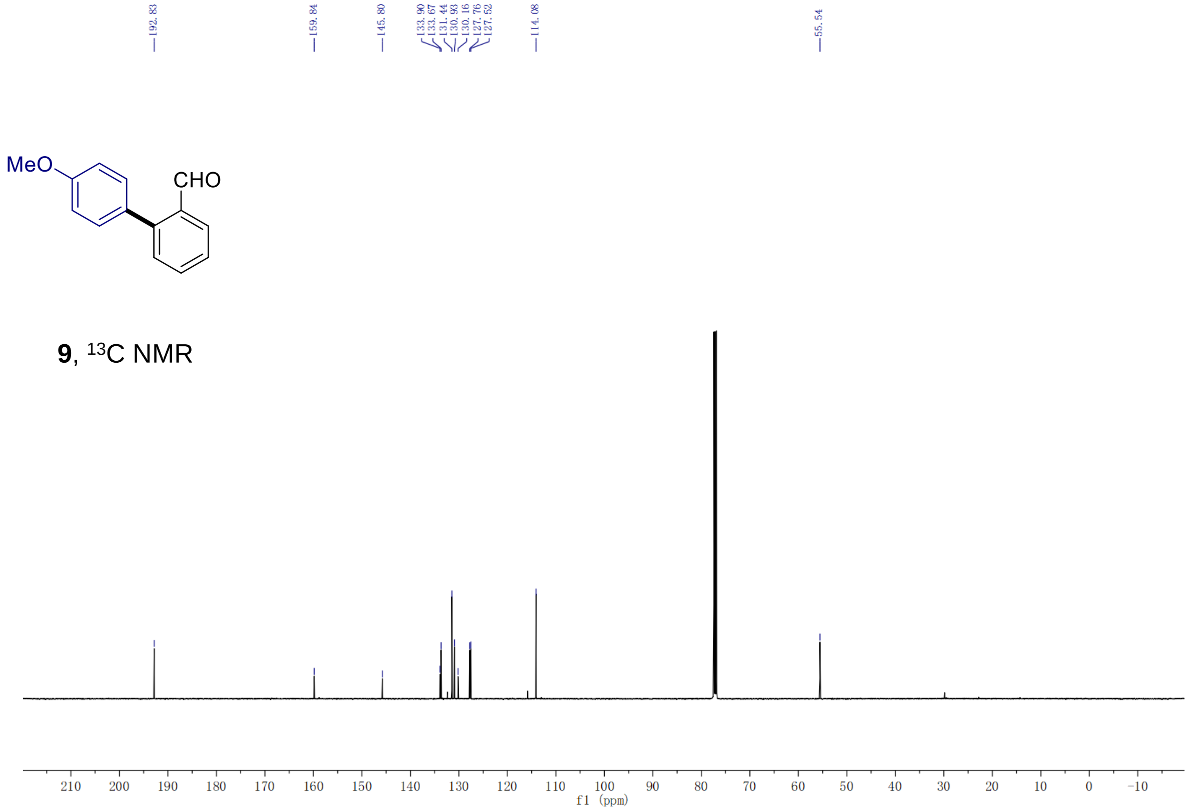

9, ^{13}C NMR

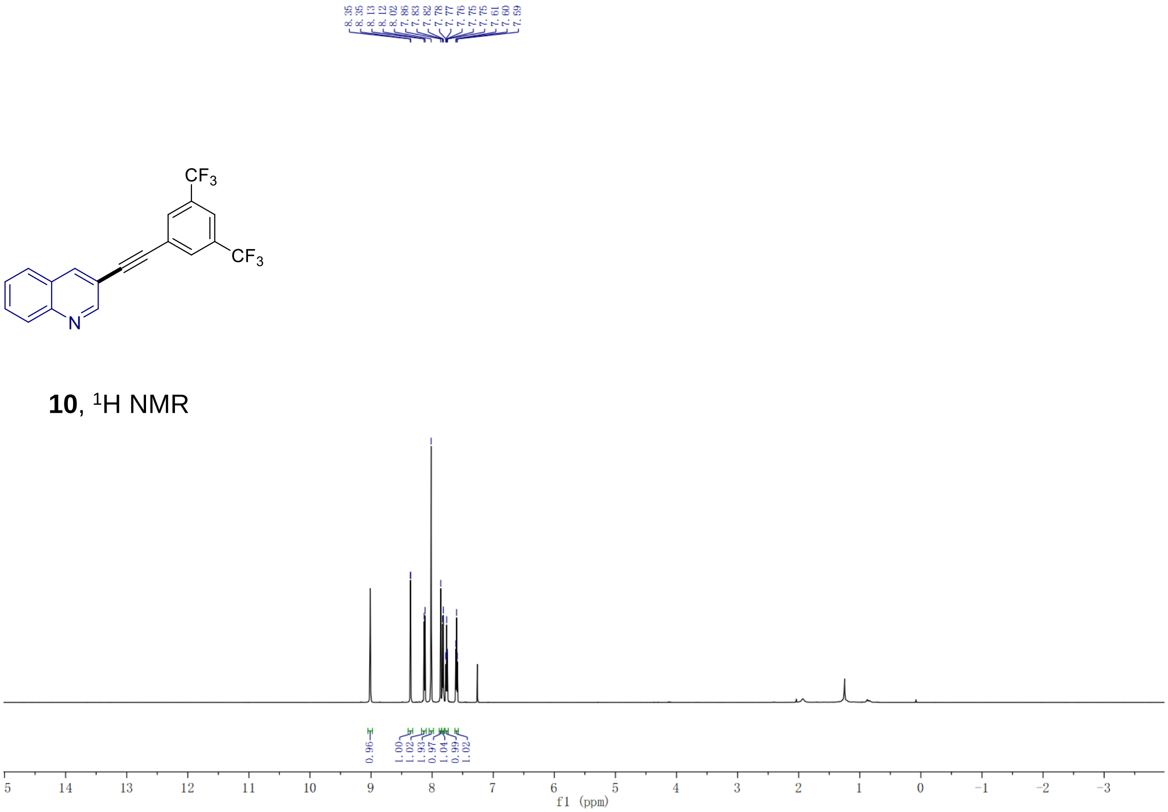

10, ^{1}H NMR

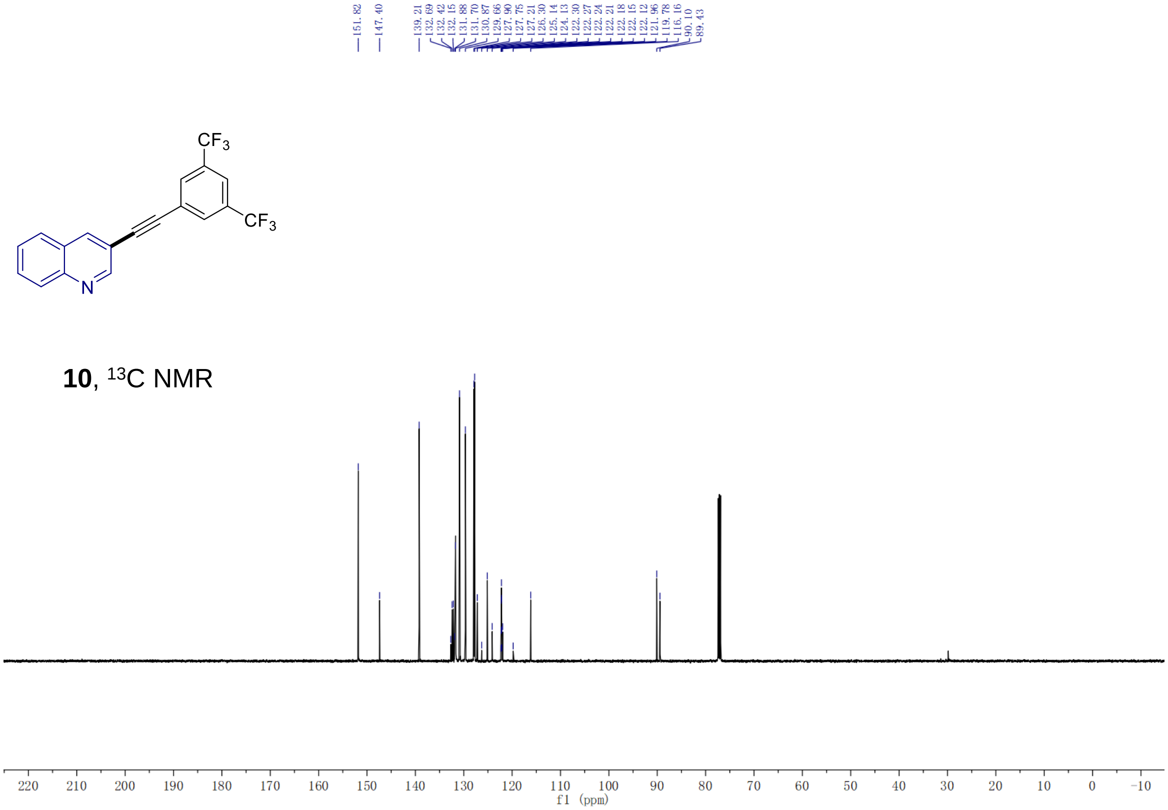

10, ^{13}C NMR

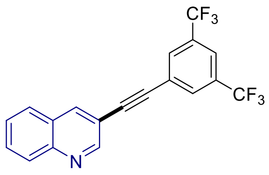

10, ^{19}F NMR

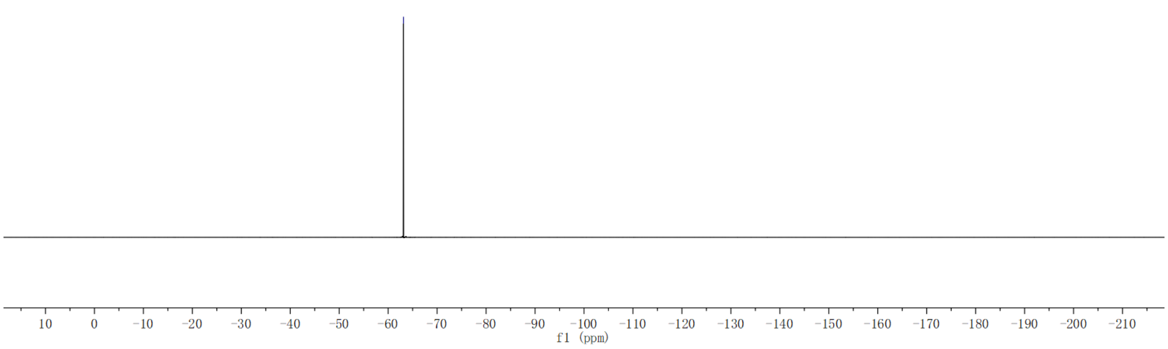

—63.14
10
0
−10
−20
−30
−40
−50
−60
−70
−80
−90
−100
−110
−120
−130
−140
−150
−160
−170
−180
−190
−200
−210
f1 (ppm)

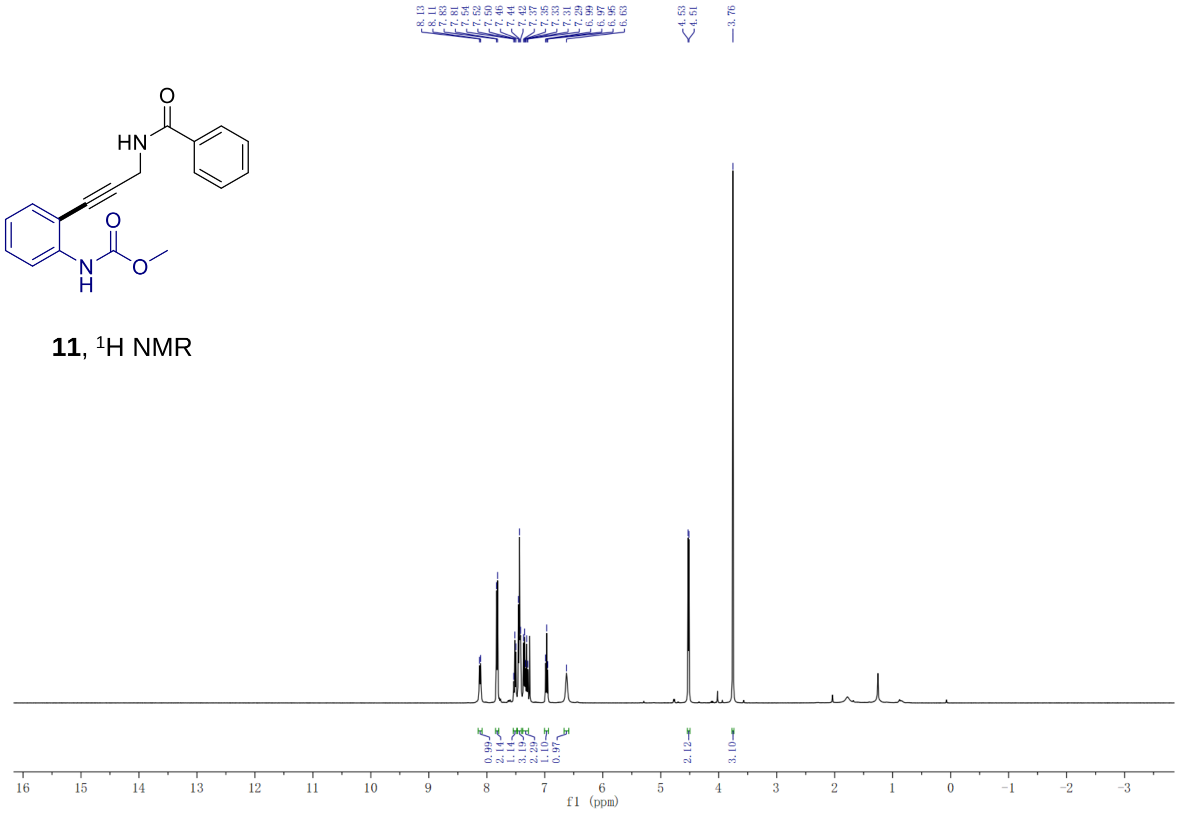

11, ¹H NMR

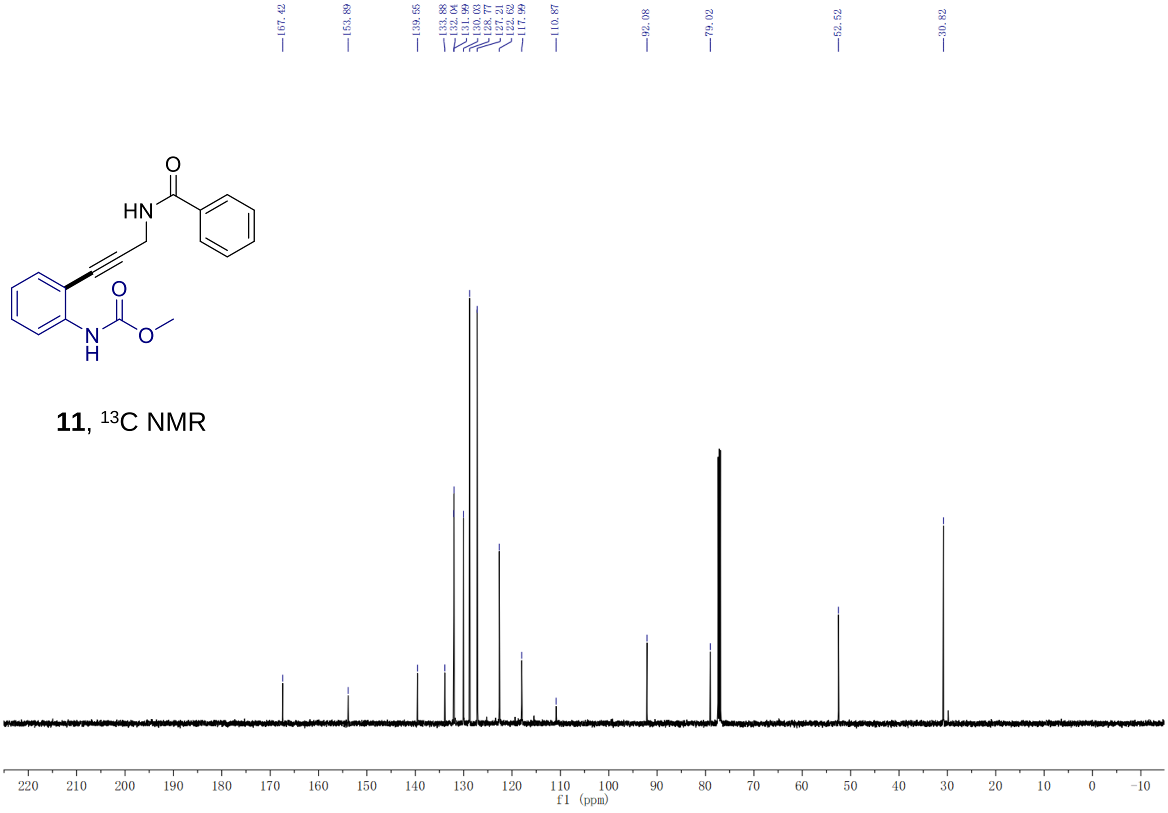

11, ¹³C NMR

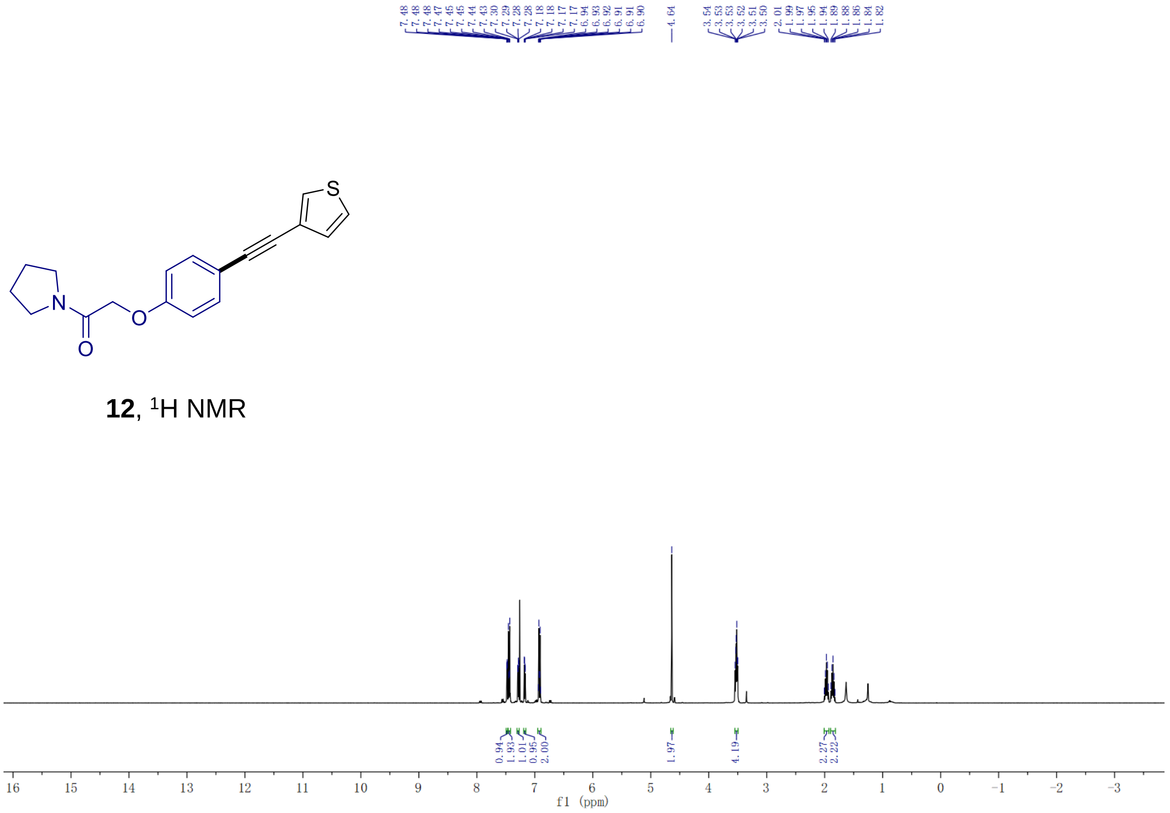
7.48 7.48 7.48 7.47 7.45 7.45 7.44 7.43 7.30 7.29 7.28 7.28 7.18 7.18 7.17 7.17 6.94 6.93 6.92 6.91 6.91 6.90
4.64
3.54 3.53 3.53 3.52 3.51 3.50
2.01 1.99 1.97 1.95 1.94 1.89 1.88 1.86 1.84 1.82
S
N
O
O
12, 1H NMR
0.94 1.93 1.01 0.95 2.00
1.97
4.19
2.27 2.22
f1 (ppm)

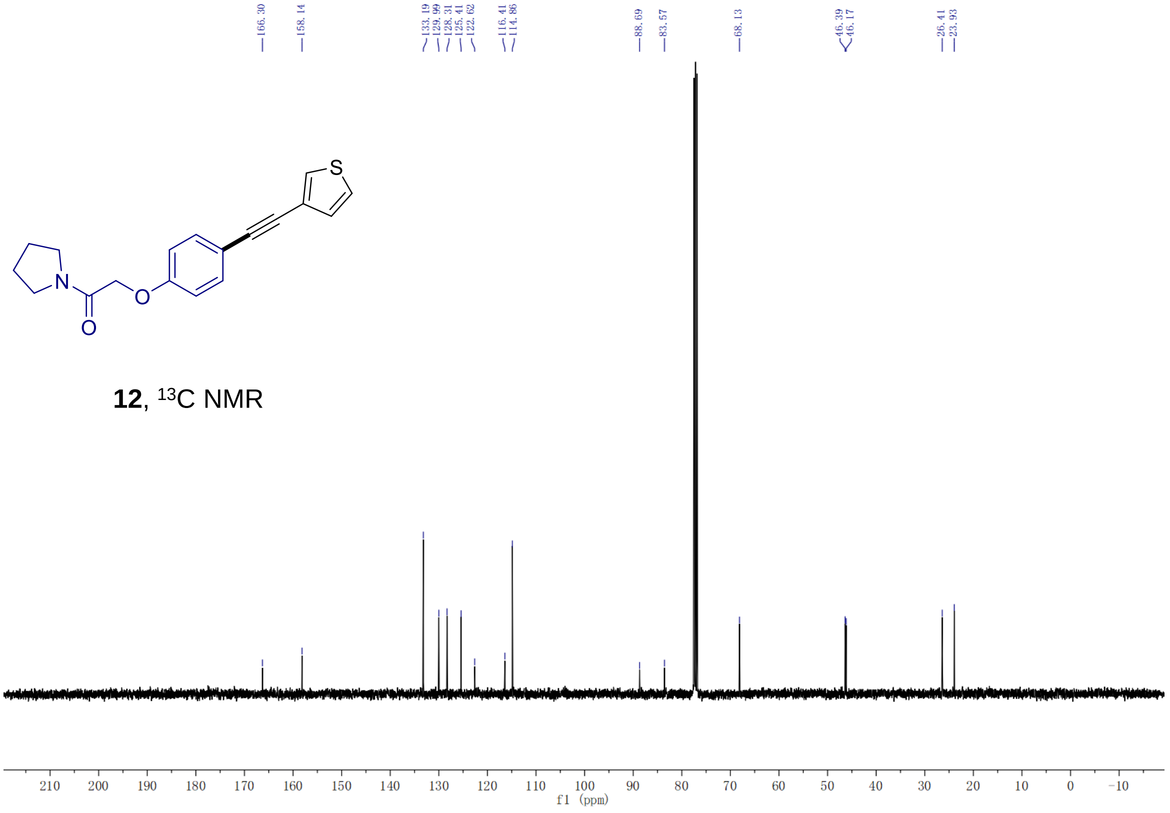
166.30
158.14
133.19 129.99 128.31 125.41 122.62
116.41 114.86
88.69
83.57
68.13
46.39 46.17
26.41 23.93
S
N
O
O
12, 13C NMR
f1 (ppm)

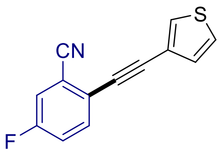

13, ^{1}H NMR

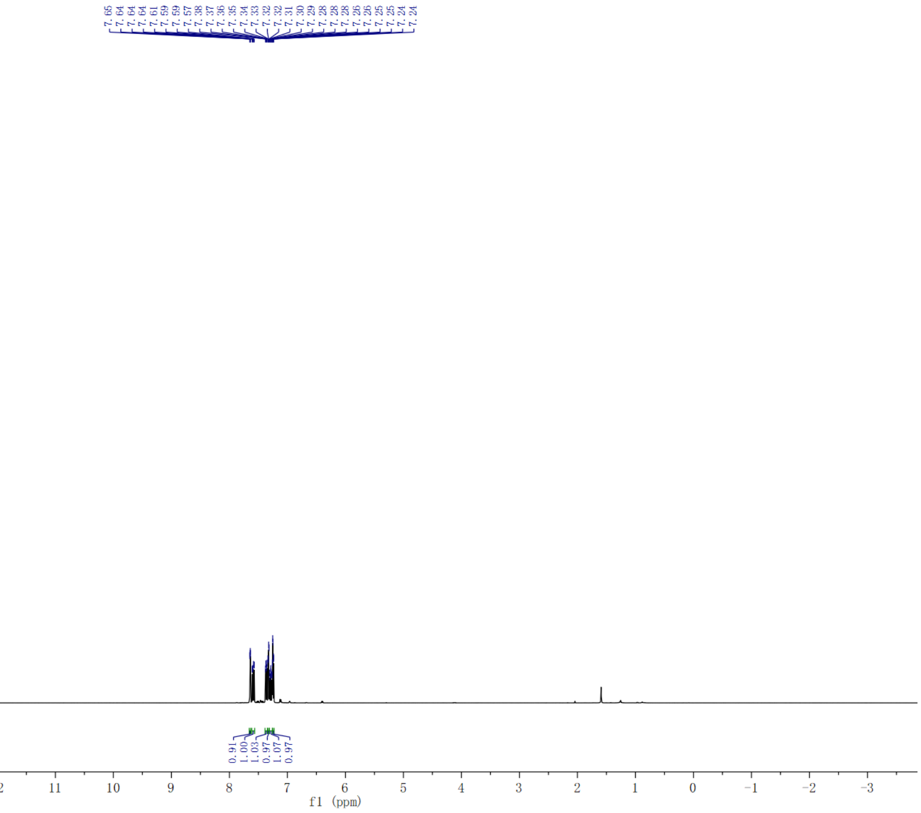

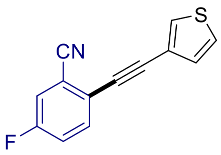

13, ^{13}C NMR

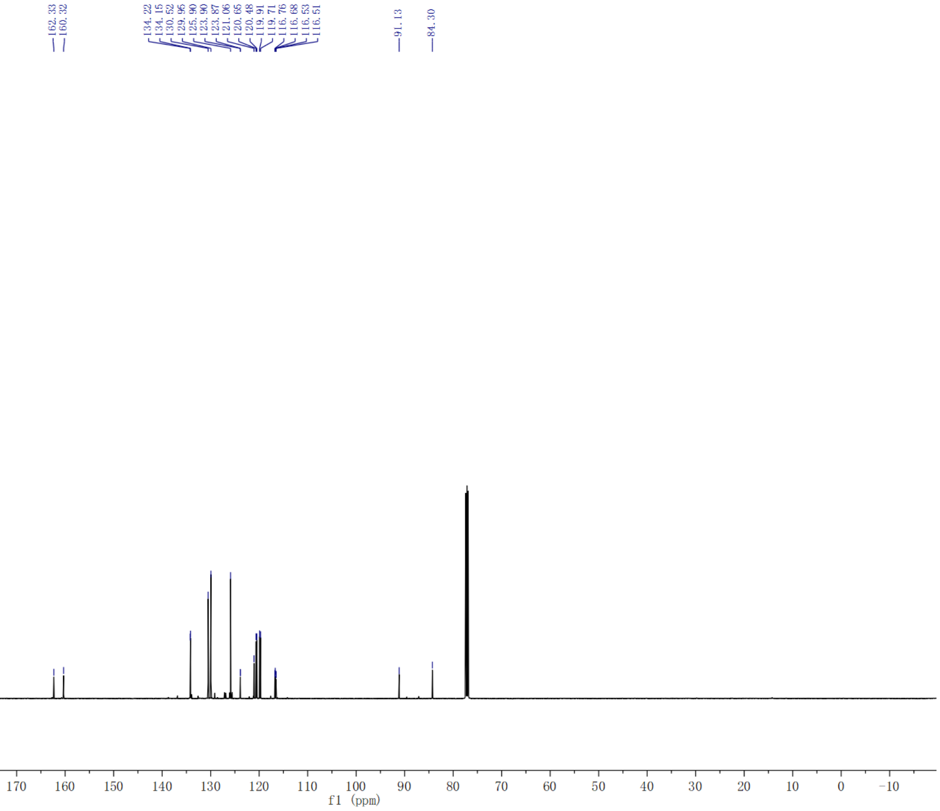

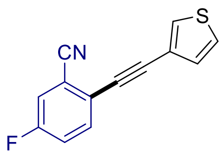

13, ^{19}F NMR

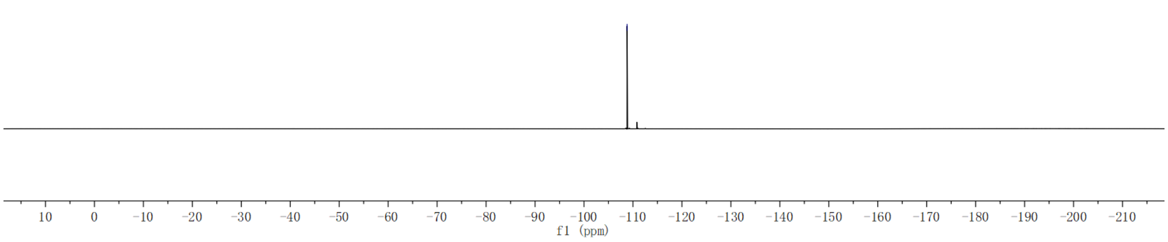

−108.80
10
0
−10
−20
−30
−40
−50
−60
−70
−80
−90
−100
−110
−120
−130
−140
−150
−160
−170
−180
−190
−200
−210
f1 (ppm)

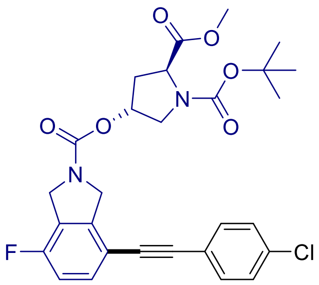

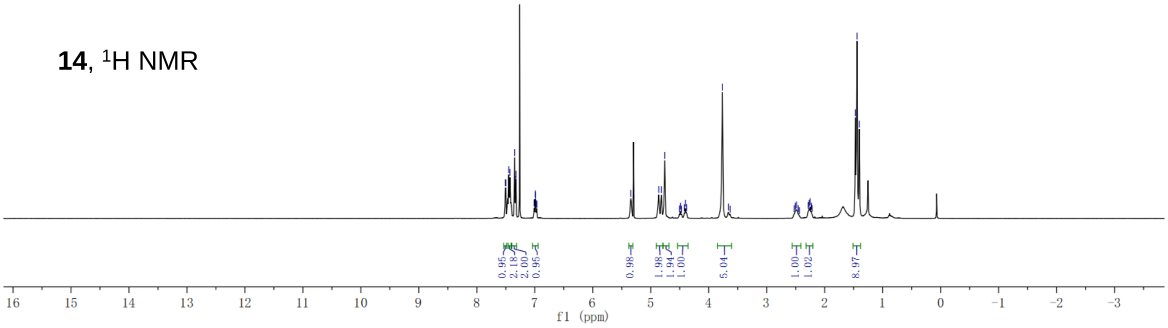

14, ^{1}H NMR

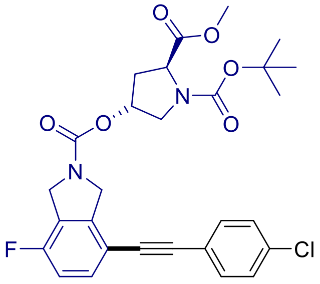

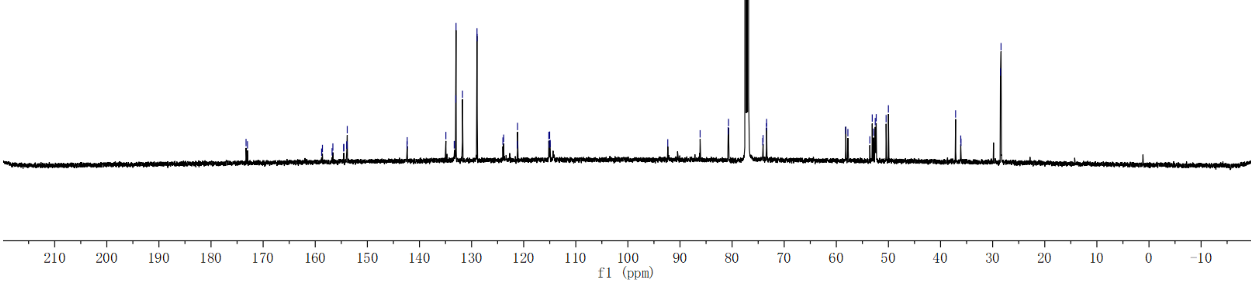

14, ^{13}C NMR

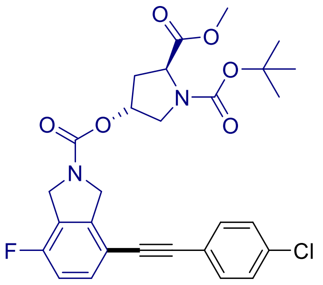

14, ^{19}F NMR

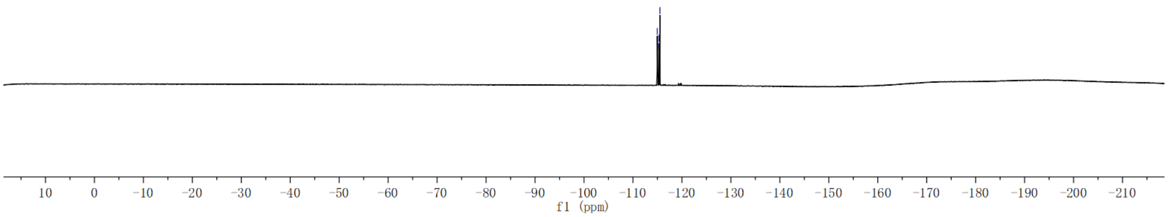
-114.97
-115.07
-115.30
-115.51
10 0 -10 -20 -30 -40 -50 -60 -70 -80 -90 -100 -110 -120 -130 -140 -150 -160 -170 -180 -190 -200 -210
f1 (ppm)

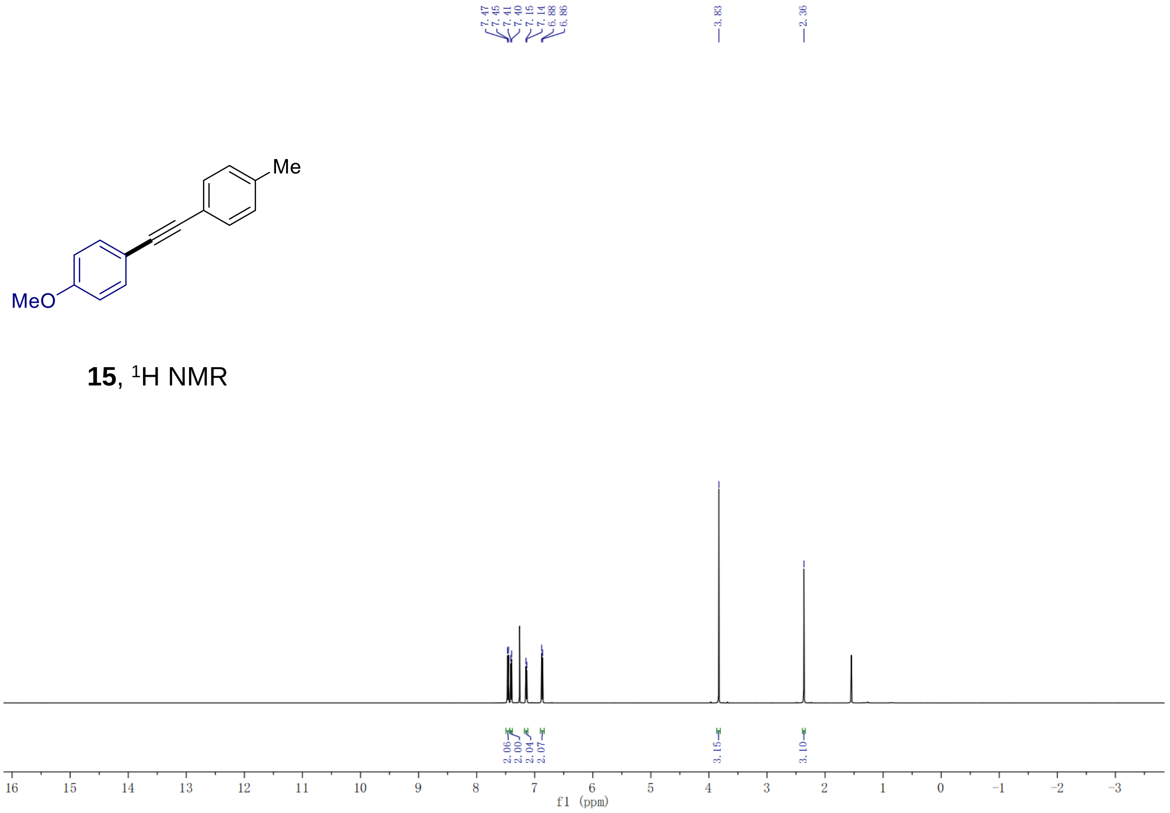
7.47
7.45
7.41
7.40
7.15
7.14
6.88
6.86
3.83
2.36
Me
MeO
15, ¹H NMR
2.06
2.00
2.04
2.07
3.15
3.10
16 15 14 13 12 11 10 9 8 7 6 5 4 3 2 1 0 -1 -2 -3
f1 (ppm)

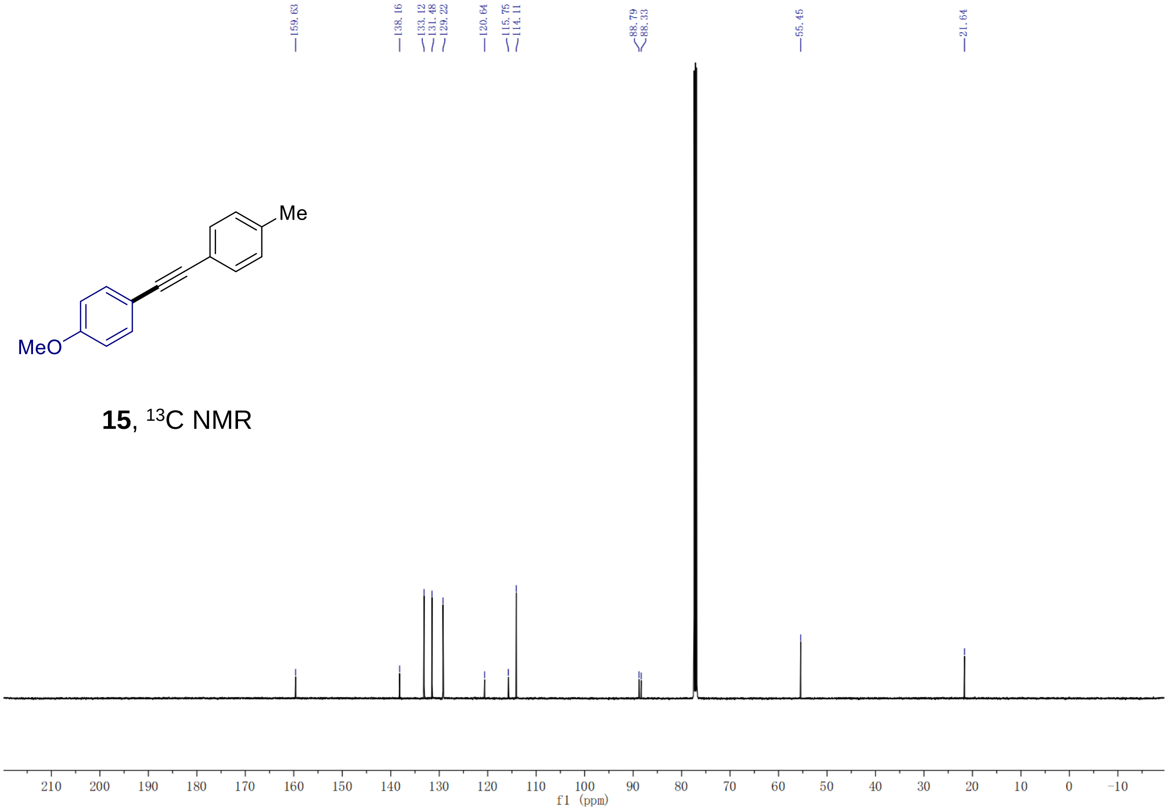
159.63
138.16
133.12
131.48
129.22
120.64
115.75
114.11
88.79
88.33
55.45
21.64
Me
MeO
15, ¹³C NMR
210 200 190 180 170 160 150 140 130 120 110 100 90 80 70 60 50 40 30 20 10 0 -10
f1 (ppm)

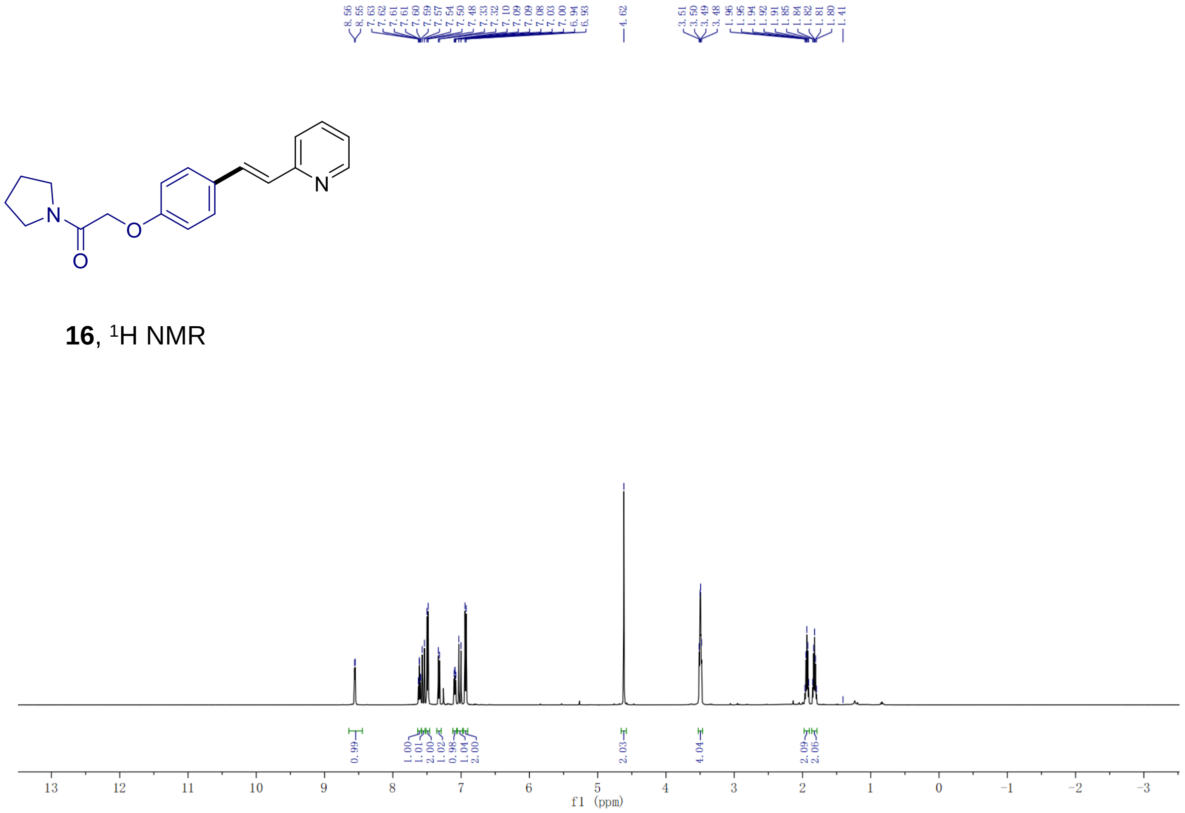

16, ^{1}H NMR

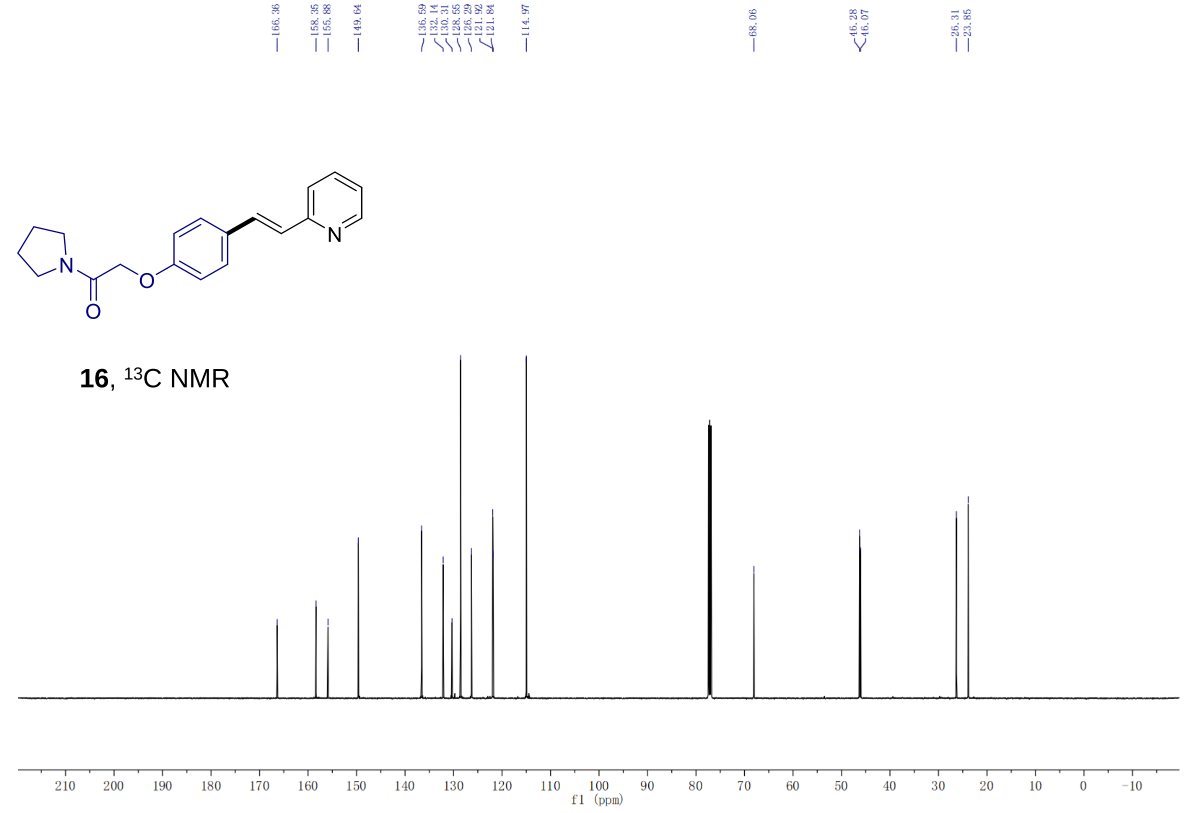

16, ^{13}C NMR

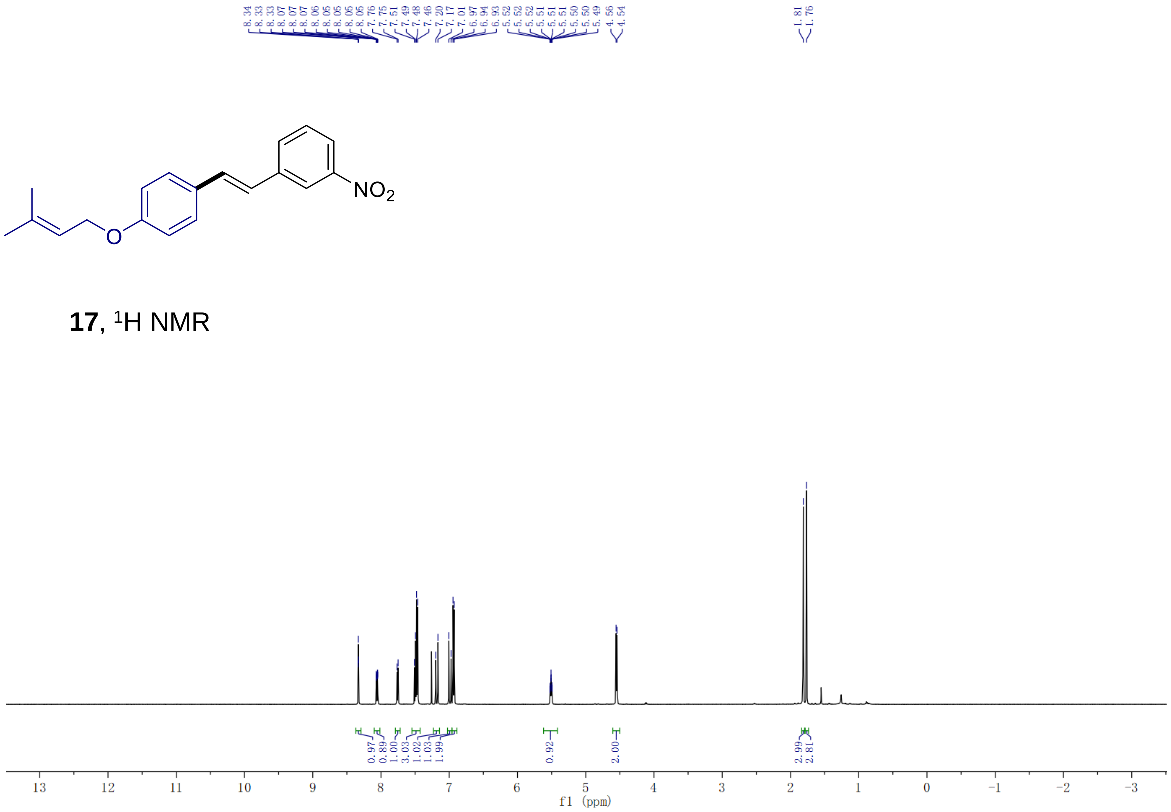

17, ^{1}H NMR

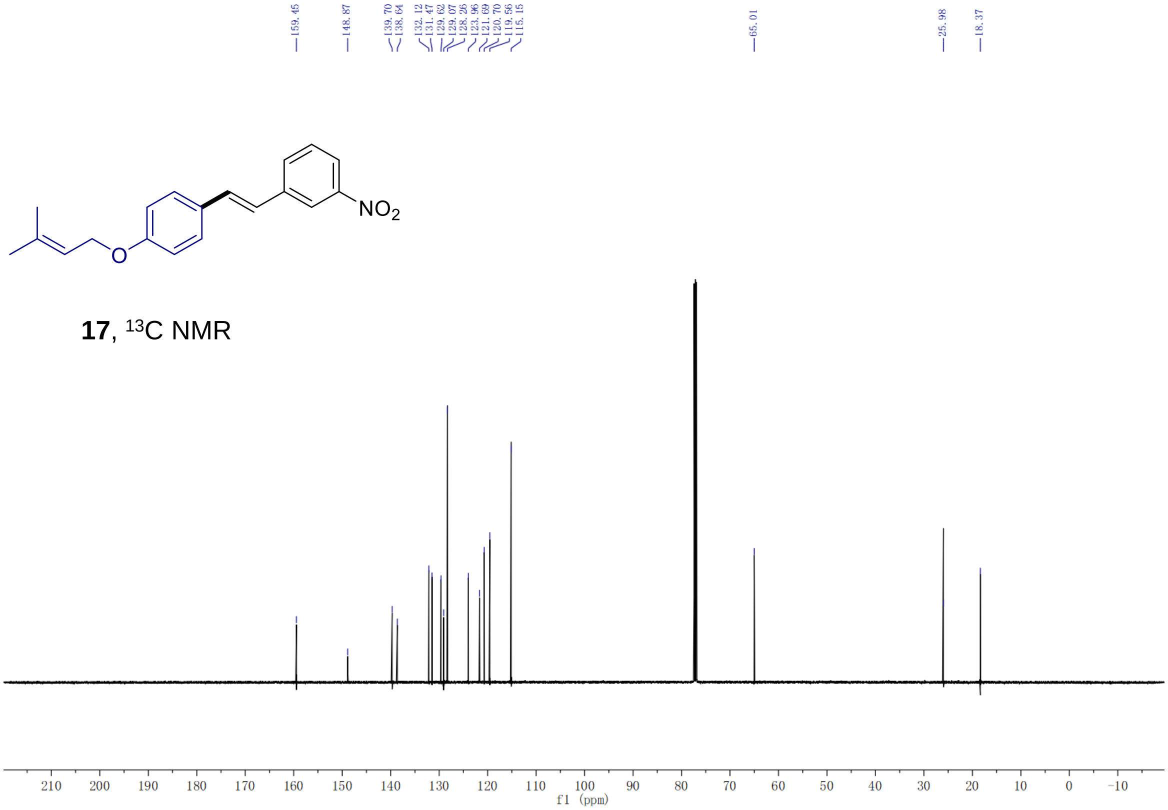

17, ^{13}C NMR

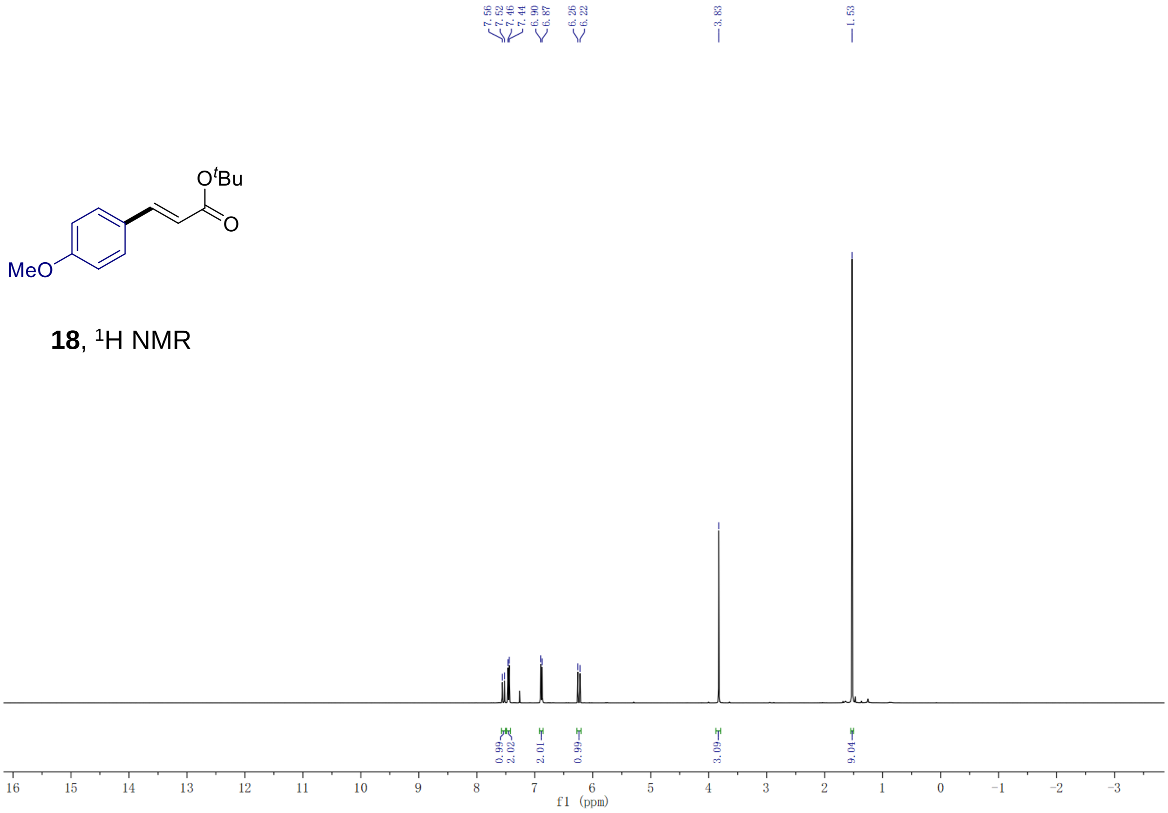

7.56
7.52
7.46
7.44
6.90
6.87
6.26
6.22
3.83
1.53
OᵗBu
O
MeO
18, ¹H NMR
0.99
2.02
2.01
0.99
3.09
9.04
16 15 14 13 12 11 10 9 8 7 6 5 4 3 2 1 0 −1 −2 −3
f1 (ppm)

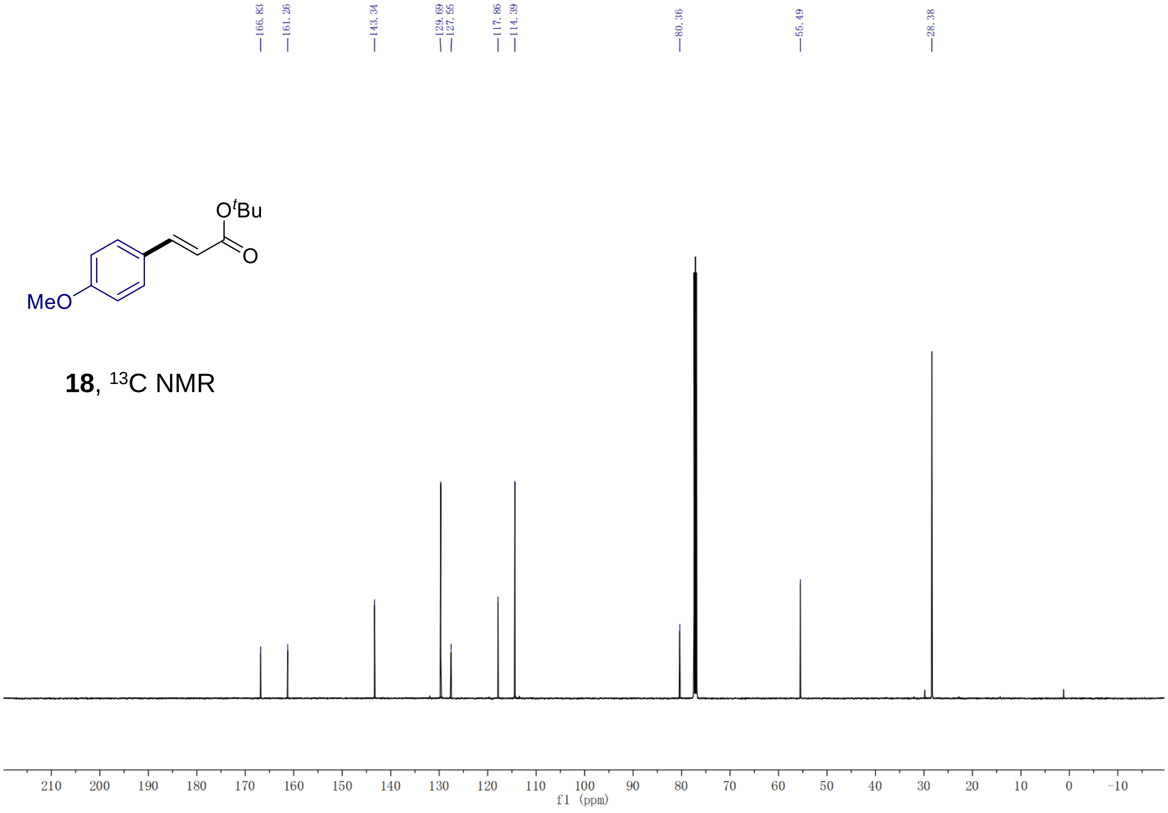

166.83
161.26
143.34
129.69
127.55
117.86
114.39
80.36
55.49
28.38
OᵗBu
O
MeO
18, ¹³C NMR
210 200 190 180 170 160 150 140 130 120 110 100 90 80 70 60 50 40 30 20 10 0 −10
f1 (ppm)

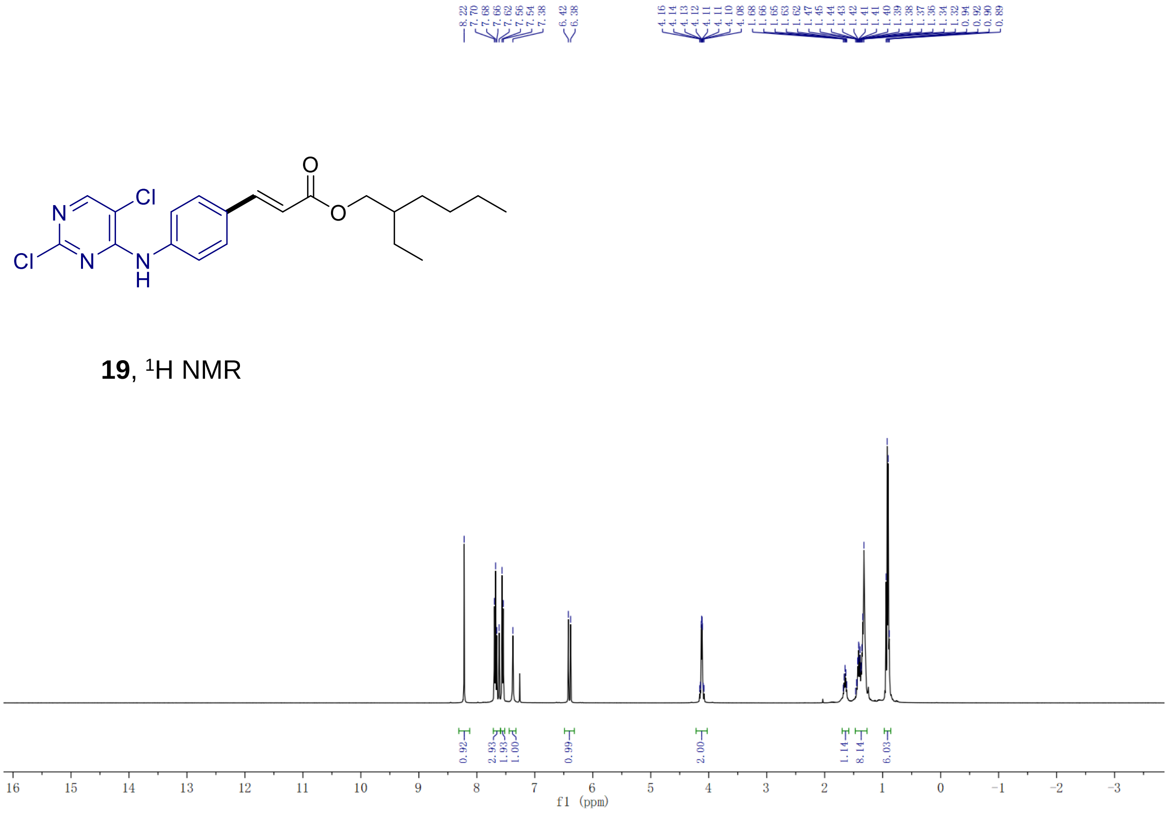

19, ^{1}H NMR

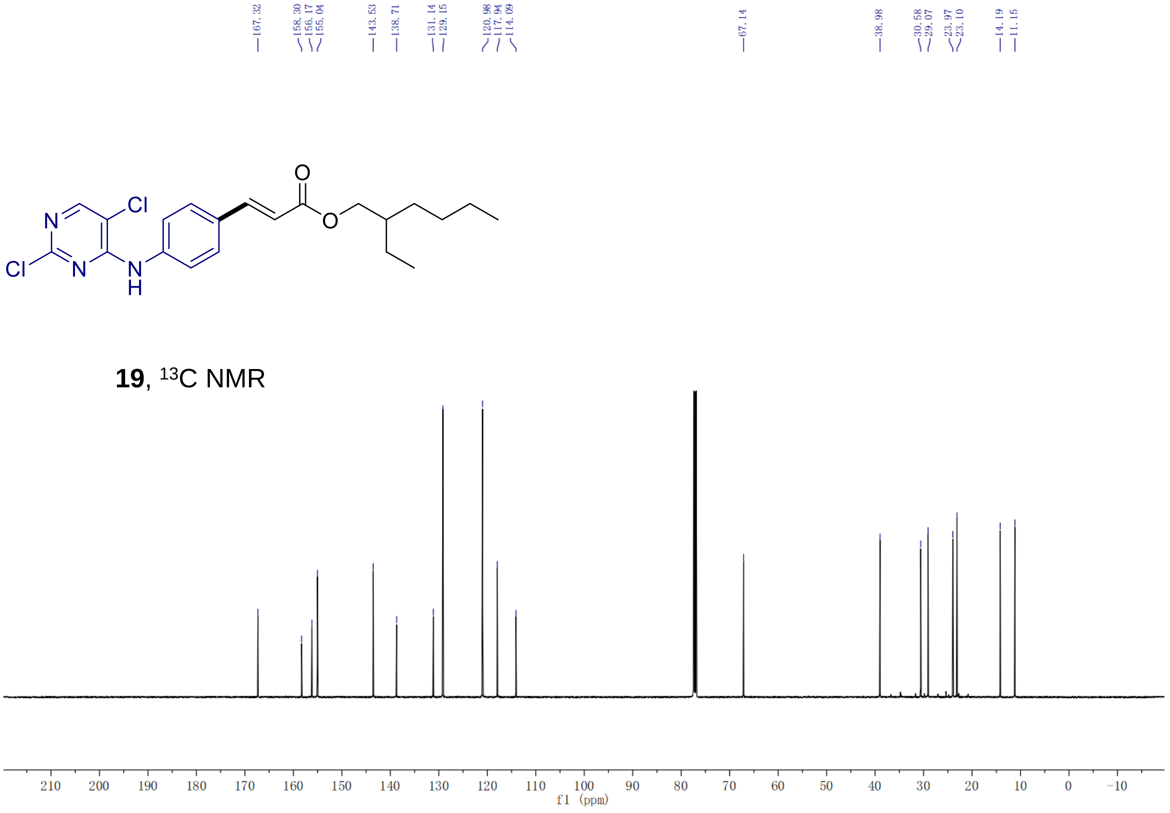

19, ^{13}C NMR

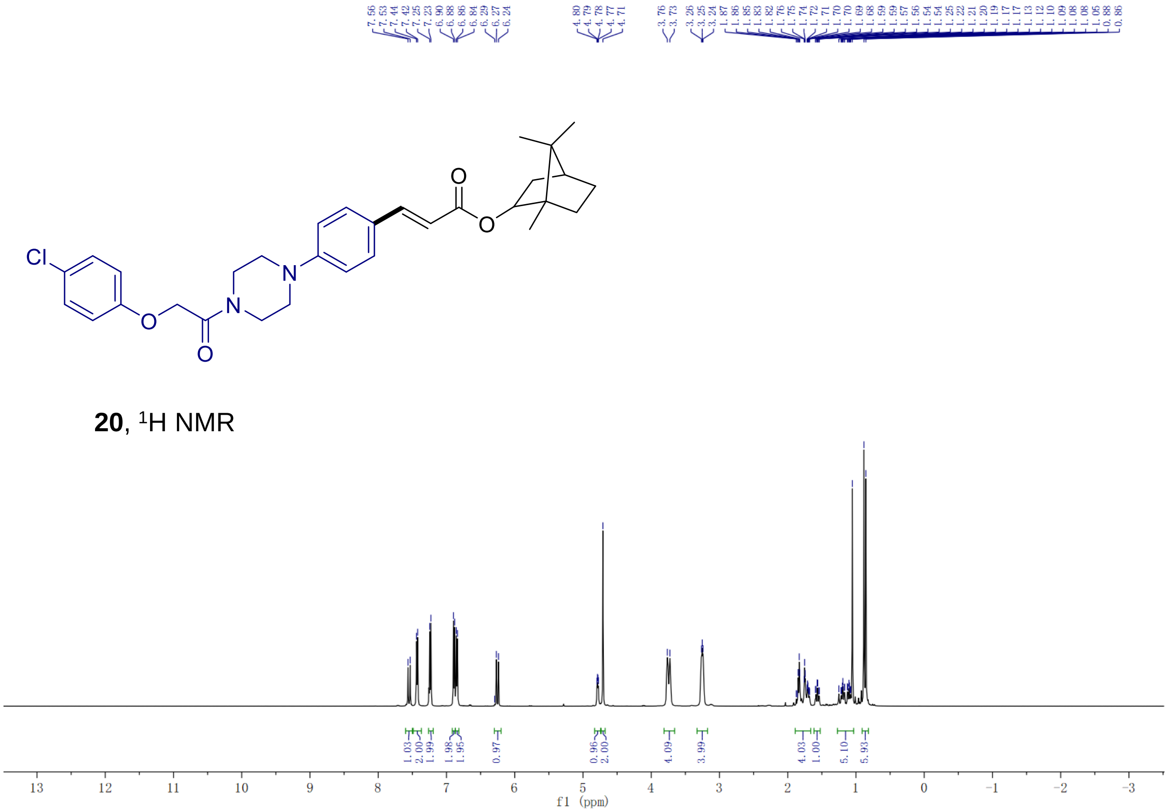

20, ^{1}H NMR

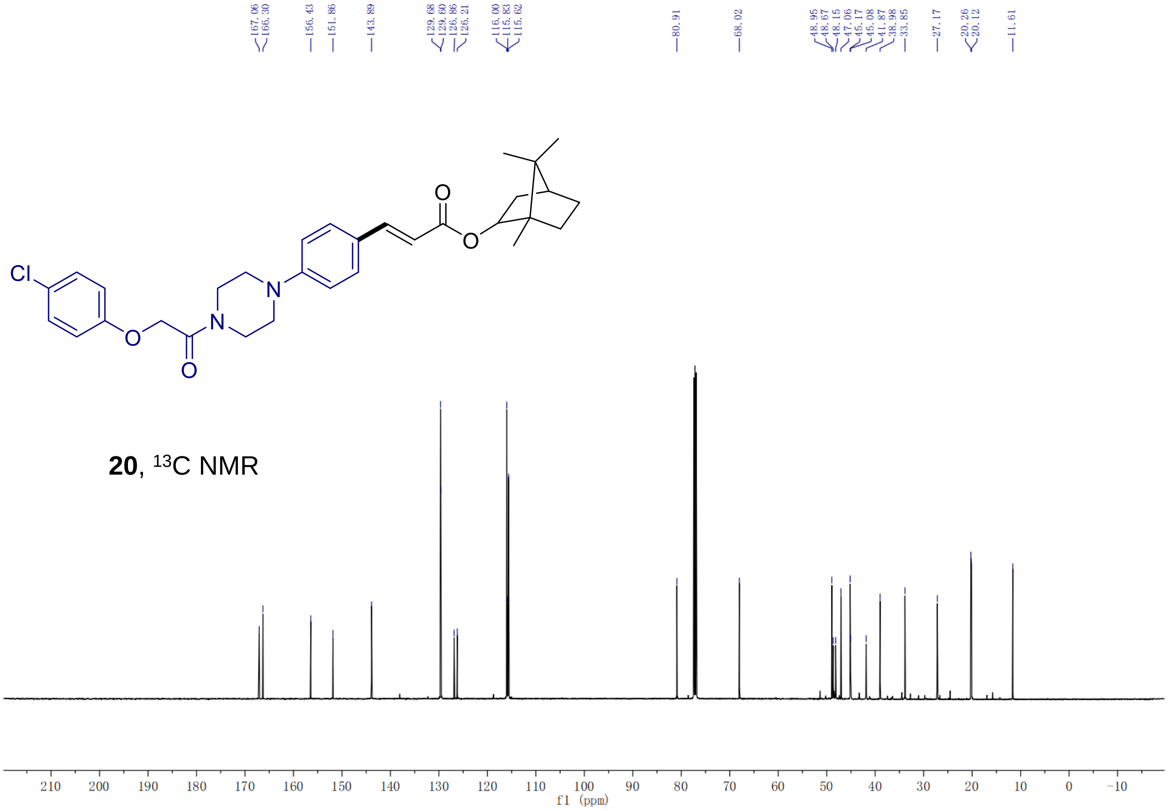

20, ^{13}C NMR

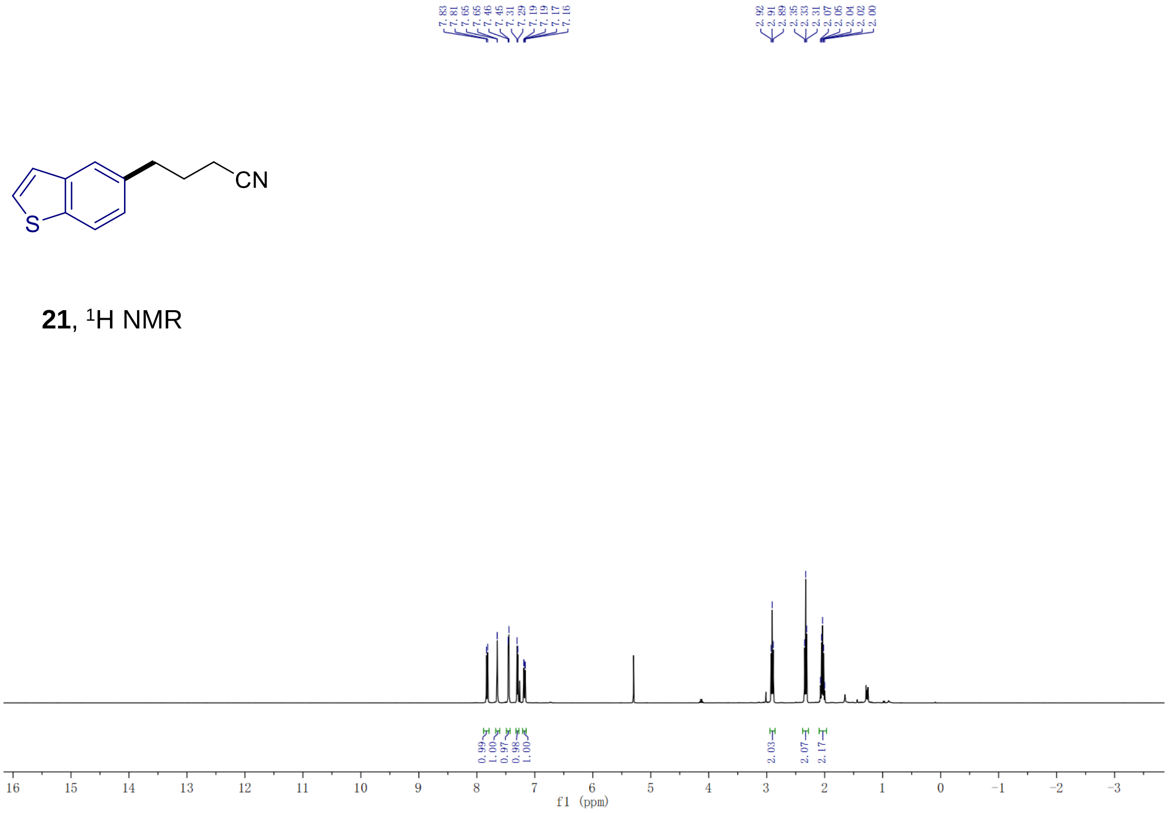

21, ^{1}H NMR

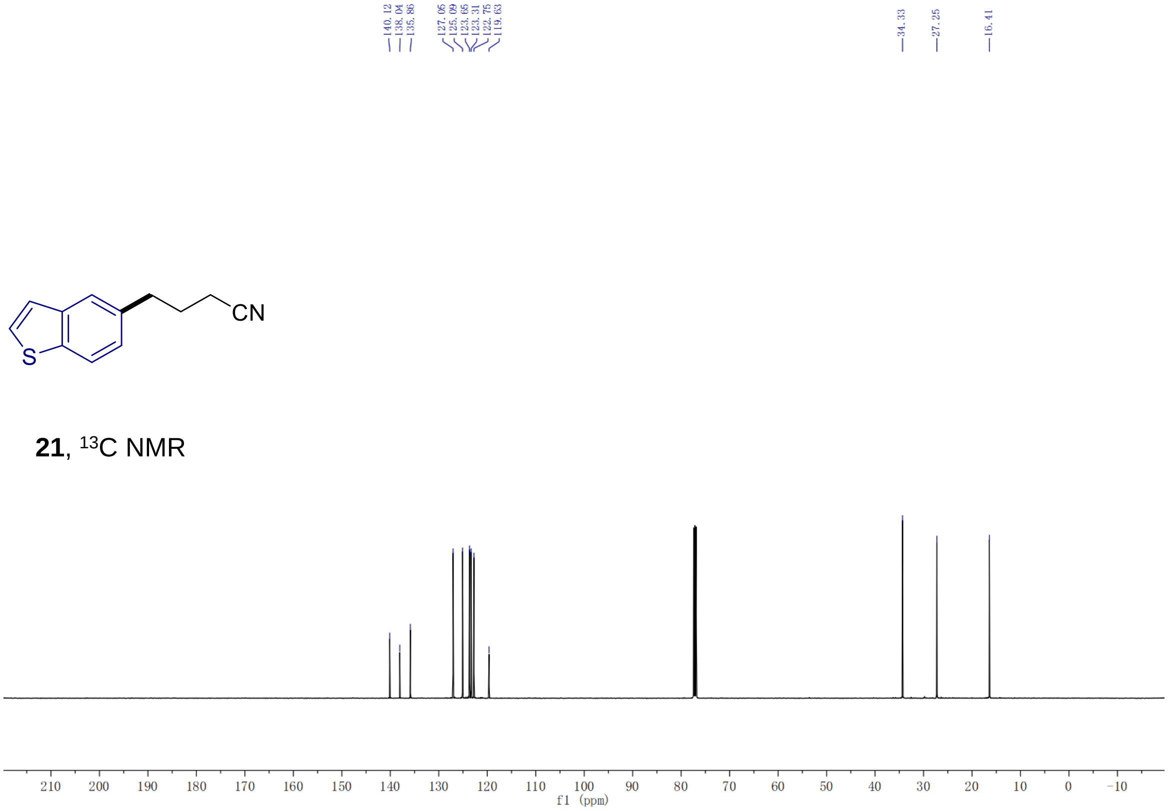

21, ^{13}C NMR

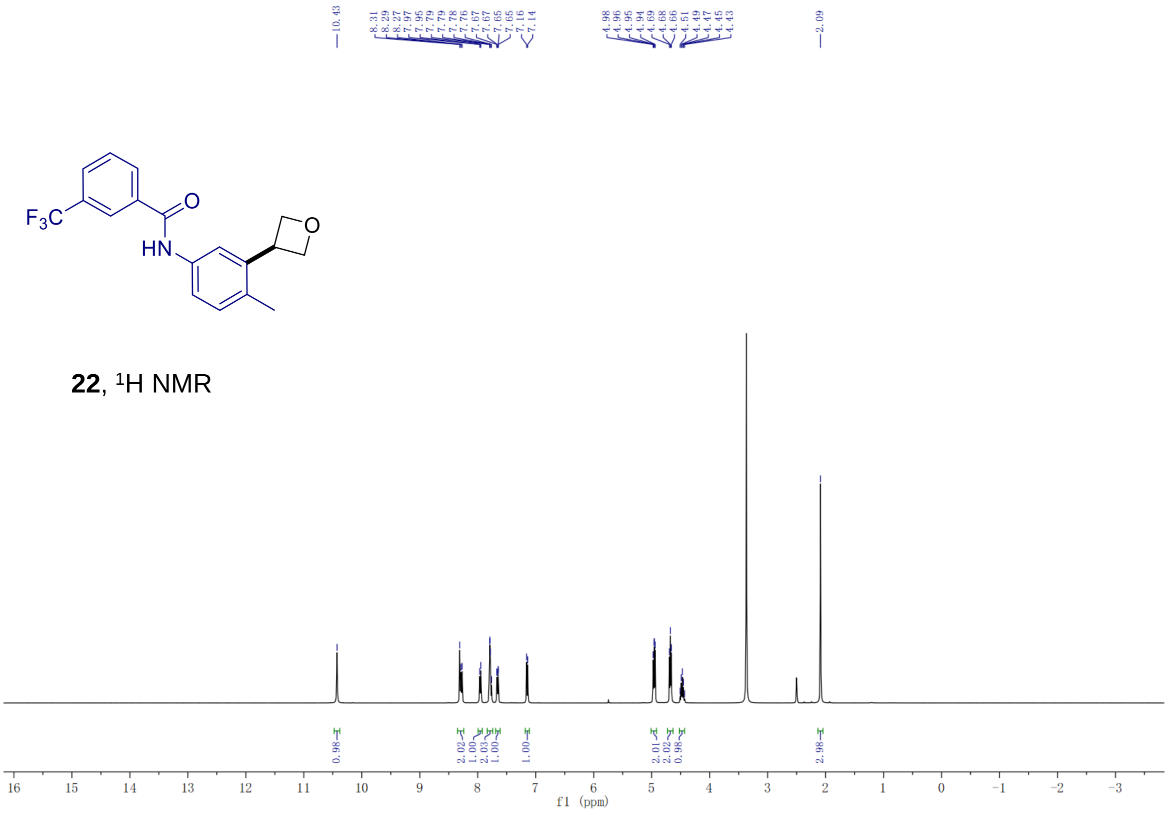
10.43
8.31 8.29 8.27 7.97 7.95 7.79 7.79 7.78 7.76 7.67 7.67 7.65 7.16 7.14
4.98 4.96 4.95 4.94 4.69 4.68 4.66 4.51 4.49 4.47 4.45 4.43
2.09
F3C
O
HN
O
22, 1H NMR
0.98
2.02 1.00 2.03 1.00
1.00
2.01 2.02 0.98
2.98
f1 (ppm)

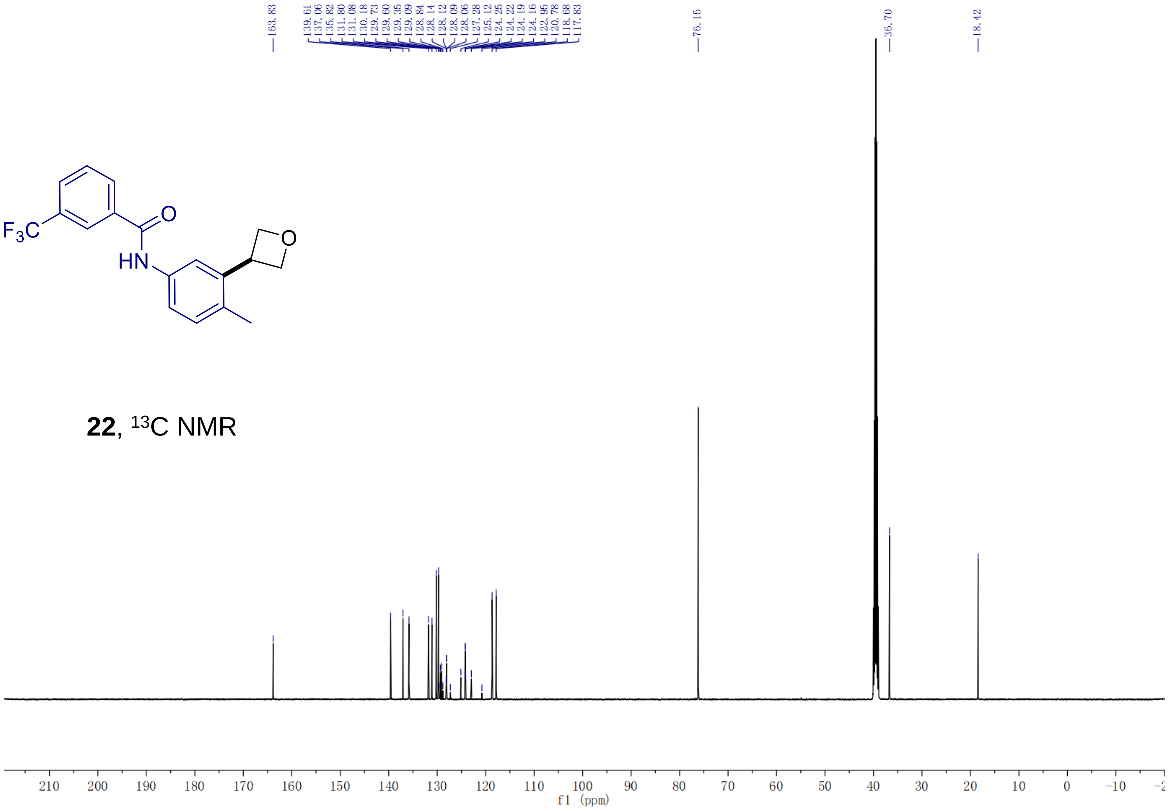
163.83
139.61 137.06 135.82 131.80 131.08 130.18 129.73 129.60 129.35 129.09 128.84 128.14 128.12 128.09 128.06 127.28 125.12 124.25 124.22 124.19 124.16 122.96 120.78 118.68 117.83
76.15
36.70
18.42
F3C
O
HN
O
22, 13C NMR
f1 (ppm)

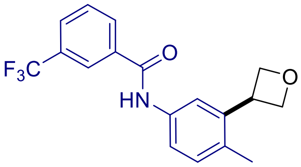
F3C
O
HN
O

22, ^{19}F NMR

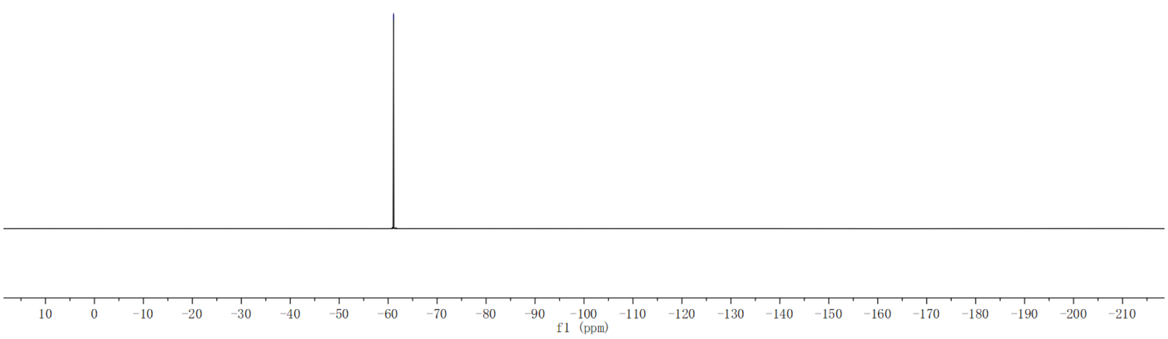
-61.09
10
0
-10
-20
-30
-40
-50
-60
-70
-80
-90
-100
-110
-120
-130
-140
-150
-160
-170
-180
-190
-200
-210
f1 (ppm)

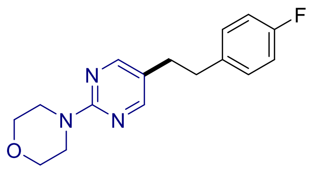

23, ¹H NMR

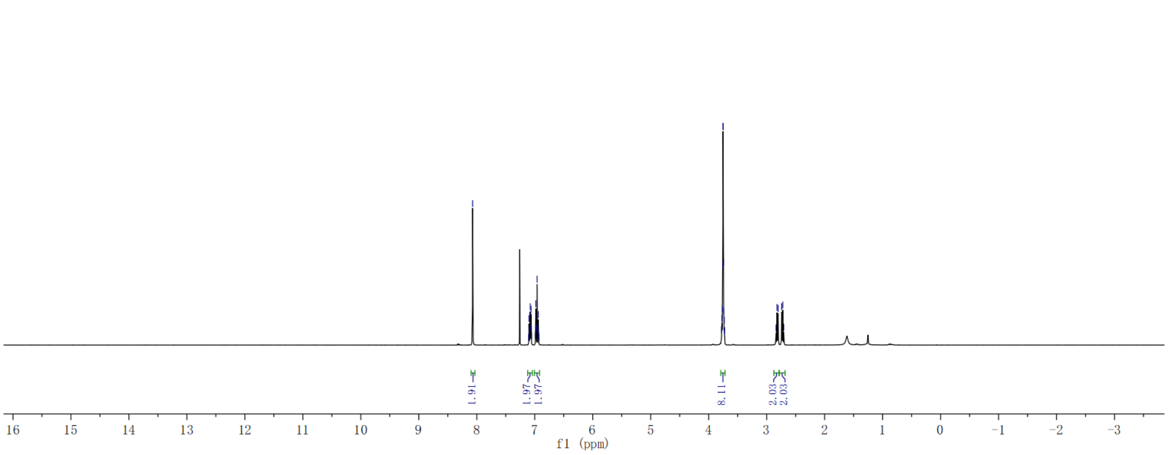

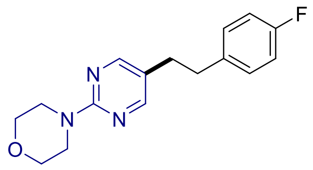

23, ¹³C NMR

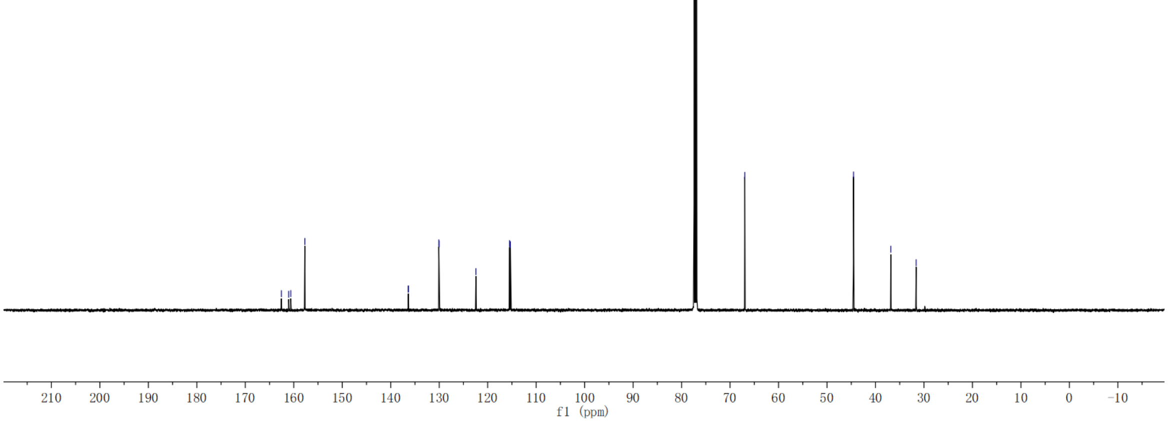

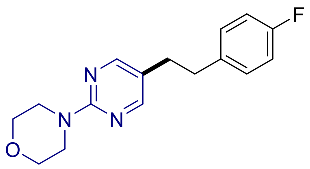

23, ^{19}F NMR

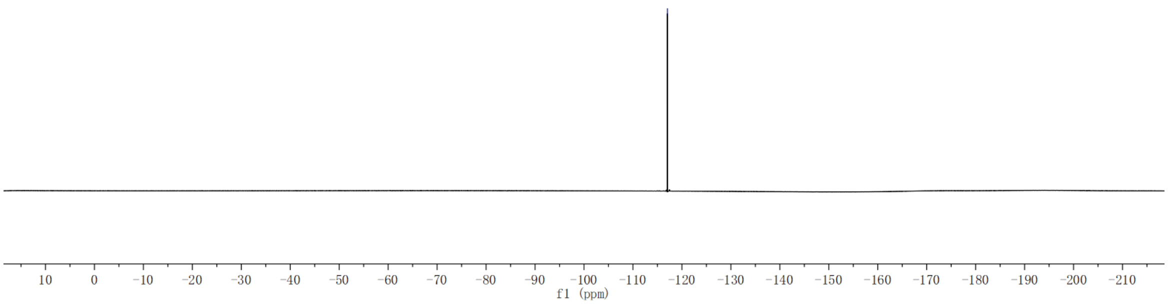

−117.04
10
0
−10
−20
−30
−40
−50
−60
−70
−80
−90
−100
−110
−120
−130
−140
−150
−160
−170
−180
−190
−200
−210
f1 (ppm)

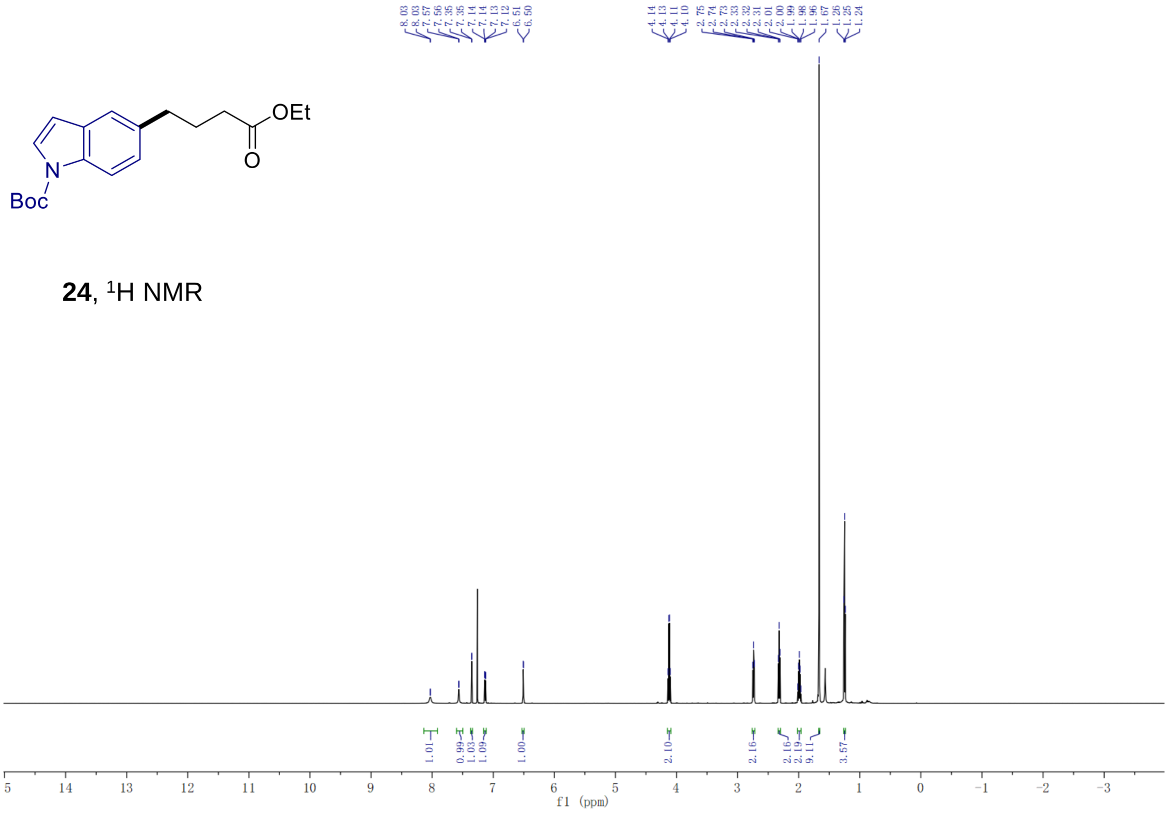

24, ^{1}H NMR

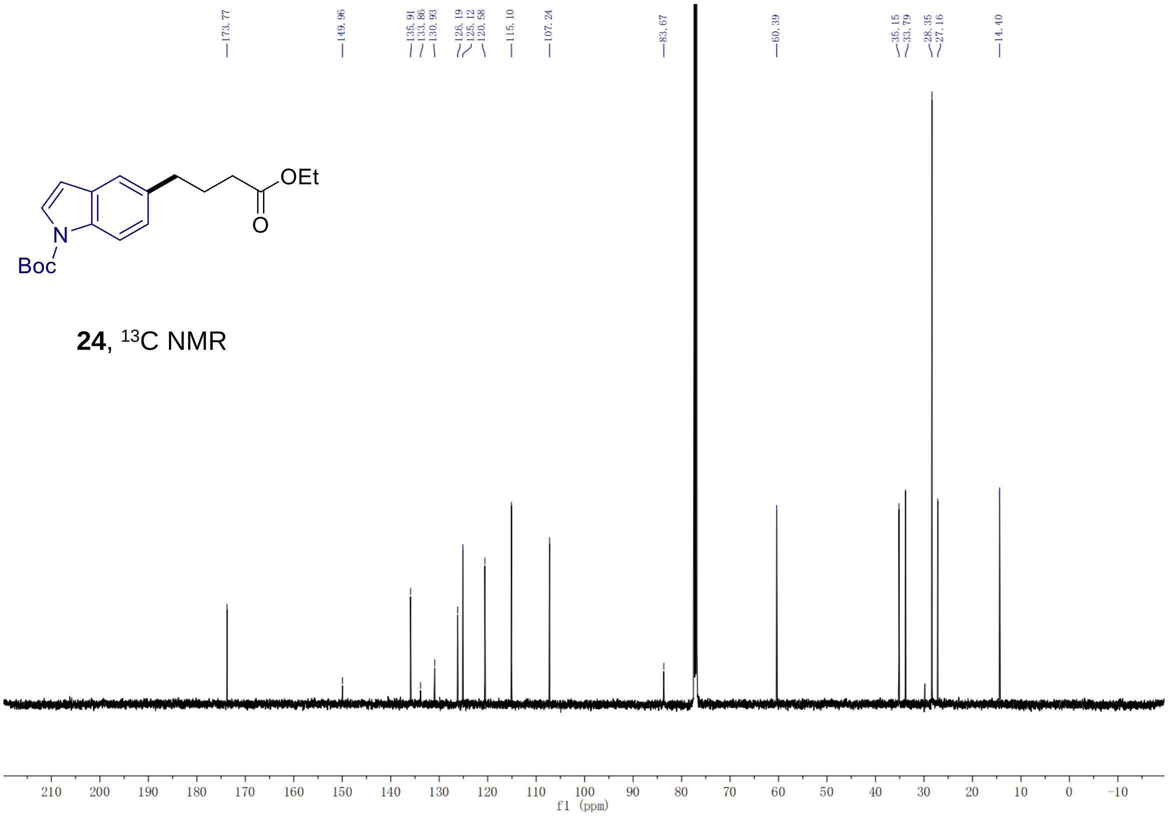

24, ^{13}C NMR

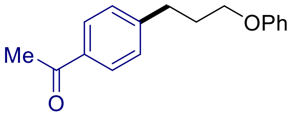

25, ^{1}H NMR

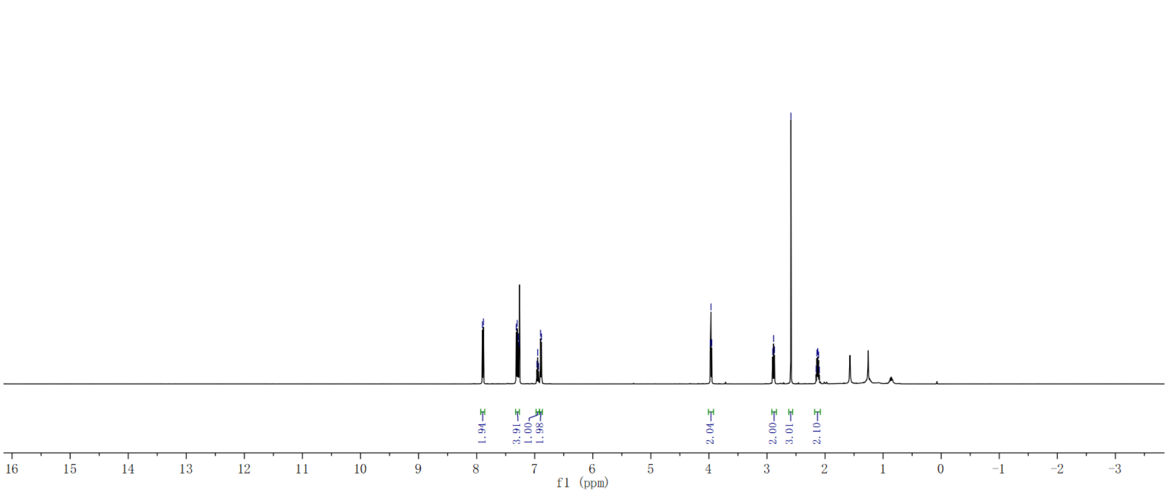

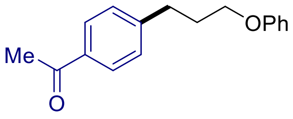

25, ^{13}C NMR

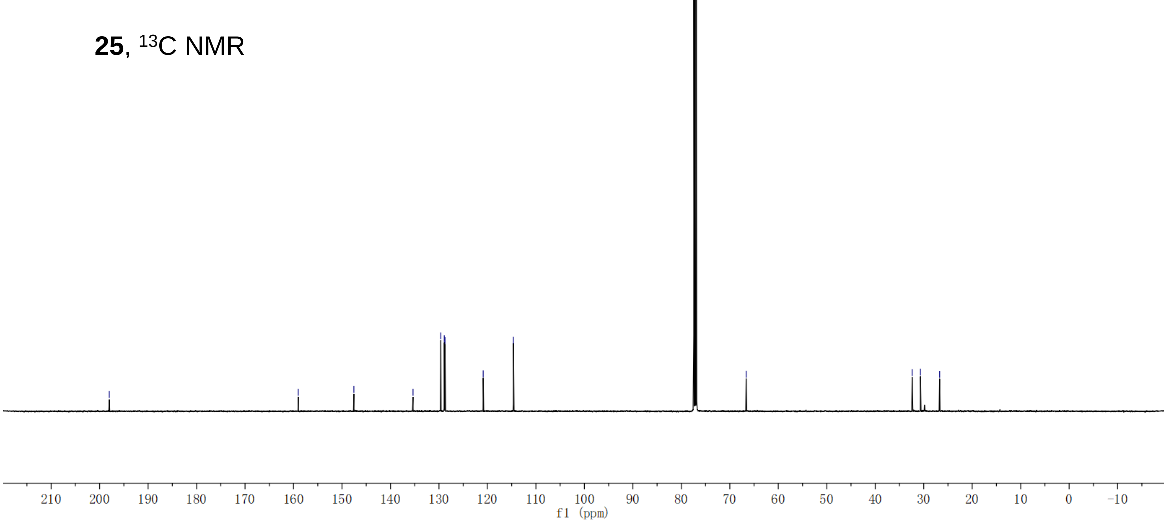

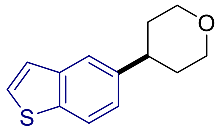

26, ^{1}H NMR

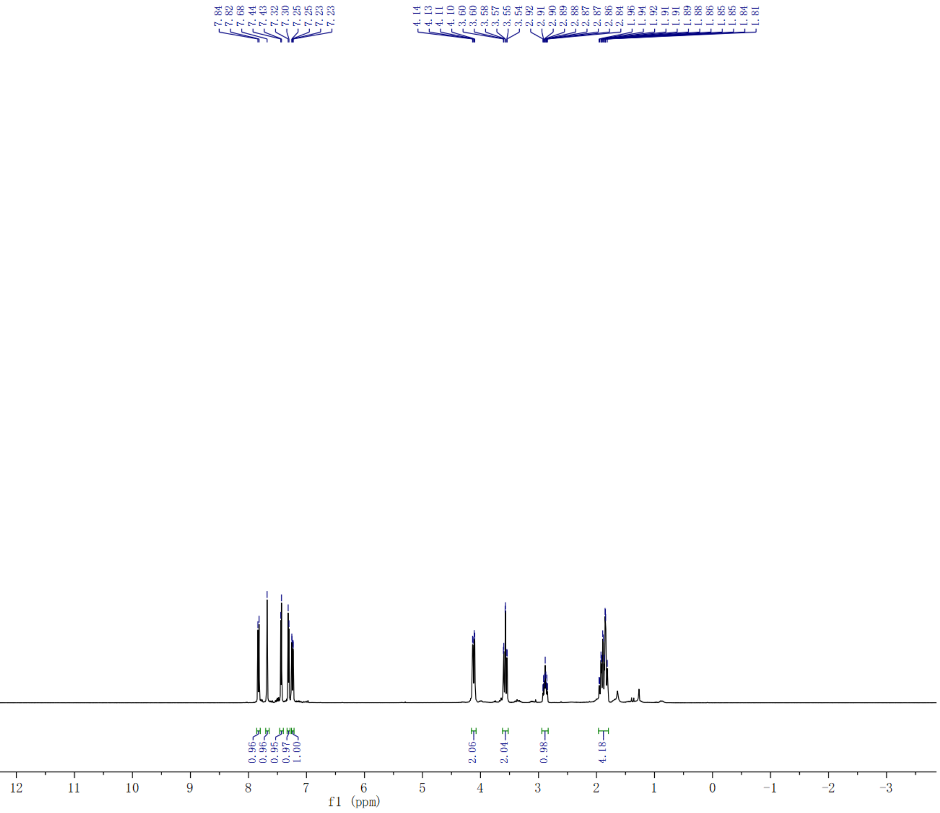

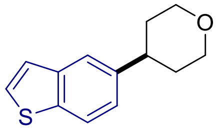

26, ^{13}C NMR

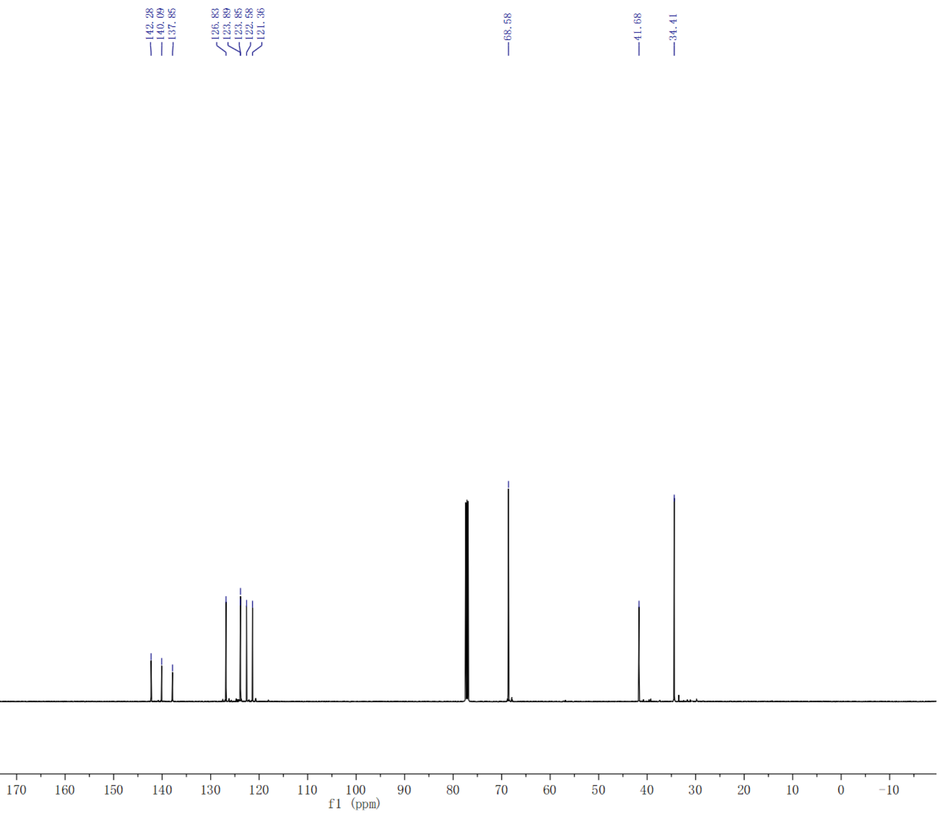

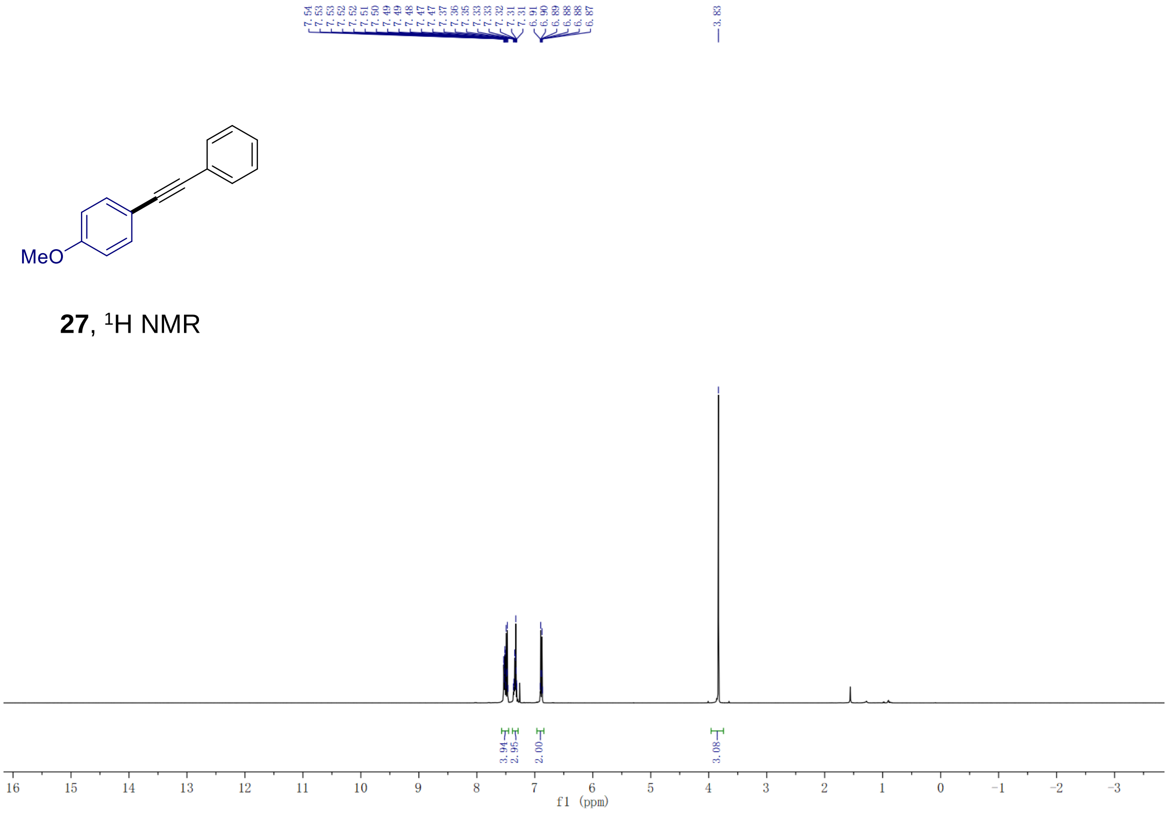

27, ^{1}H NMR

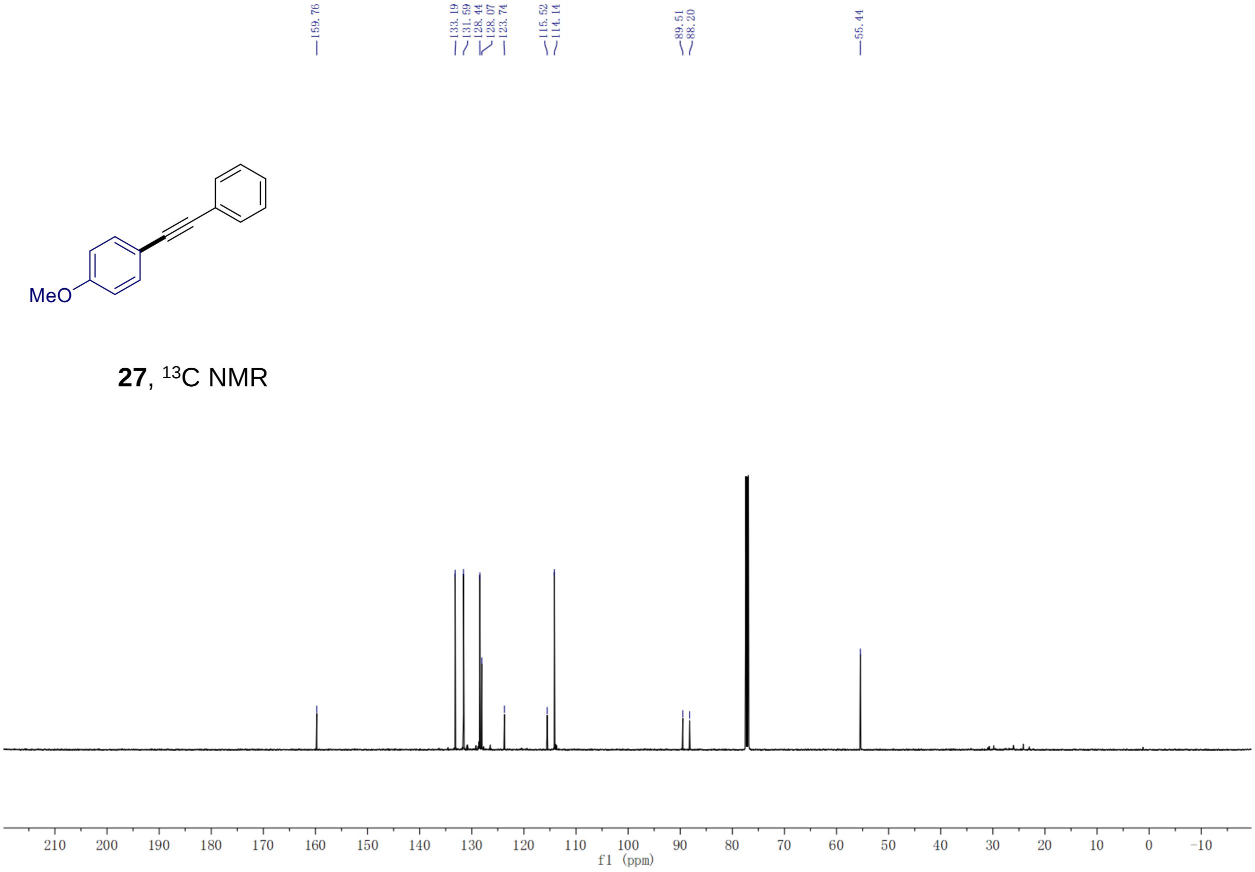

27, ^{13}C NMR

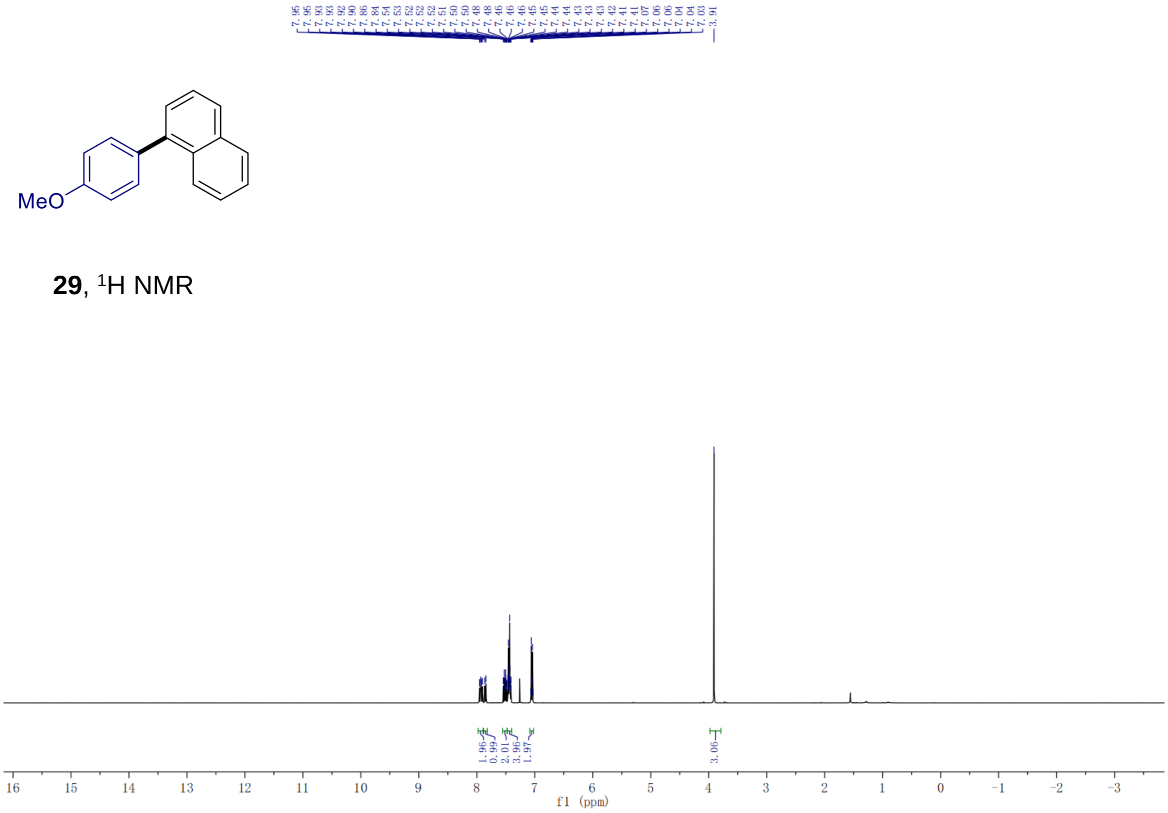

29, ^{1}H NMR

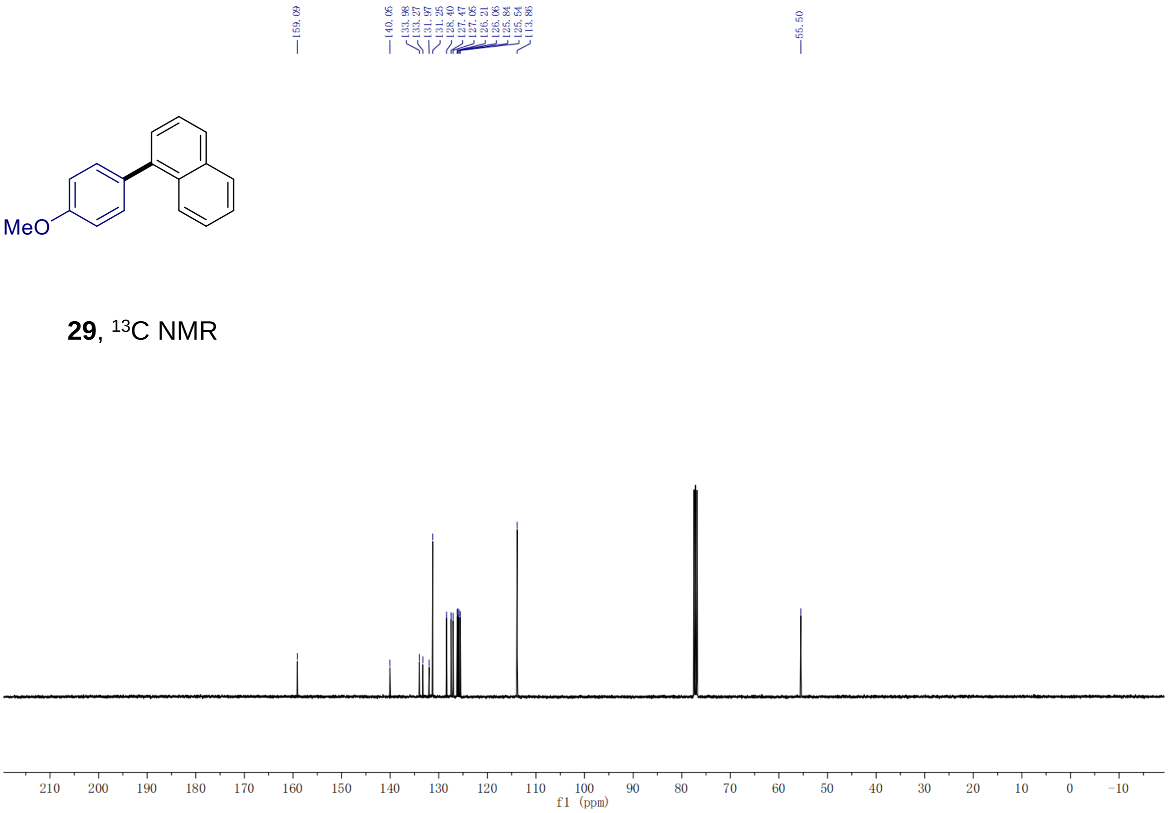

29, ^{13}C NMR

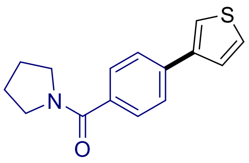

32, ^{1}H NMR

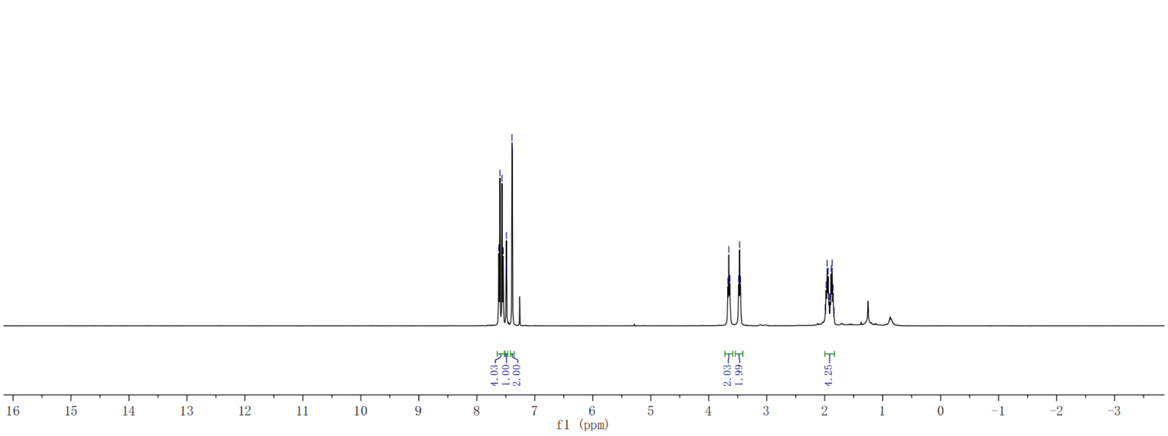

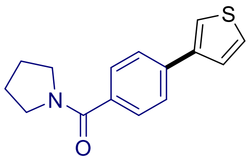

32, ^{13}C NMR

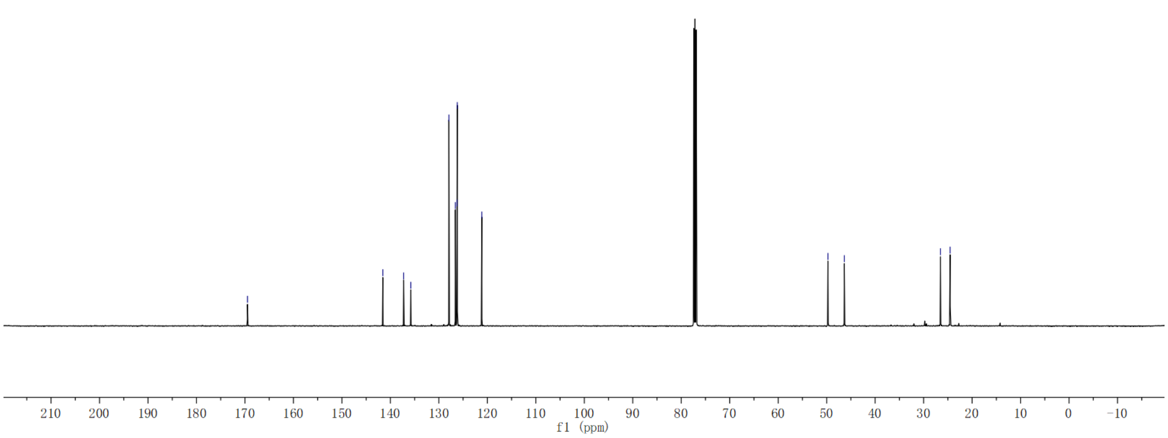

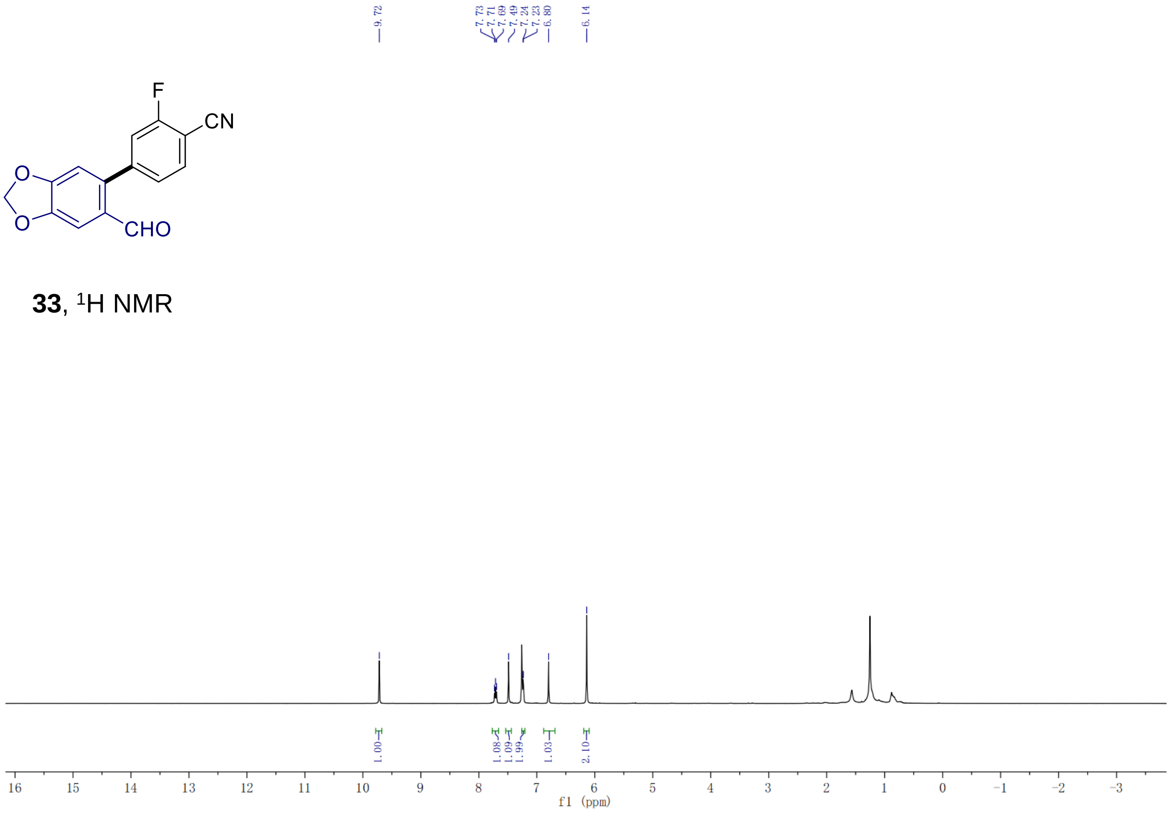

33, ^{1}H NMR

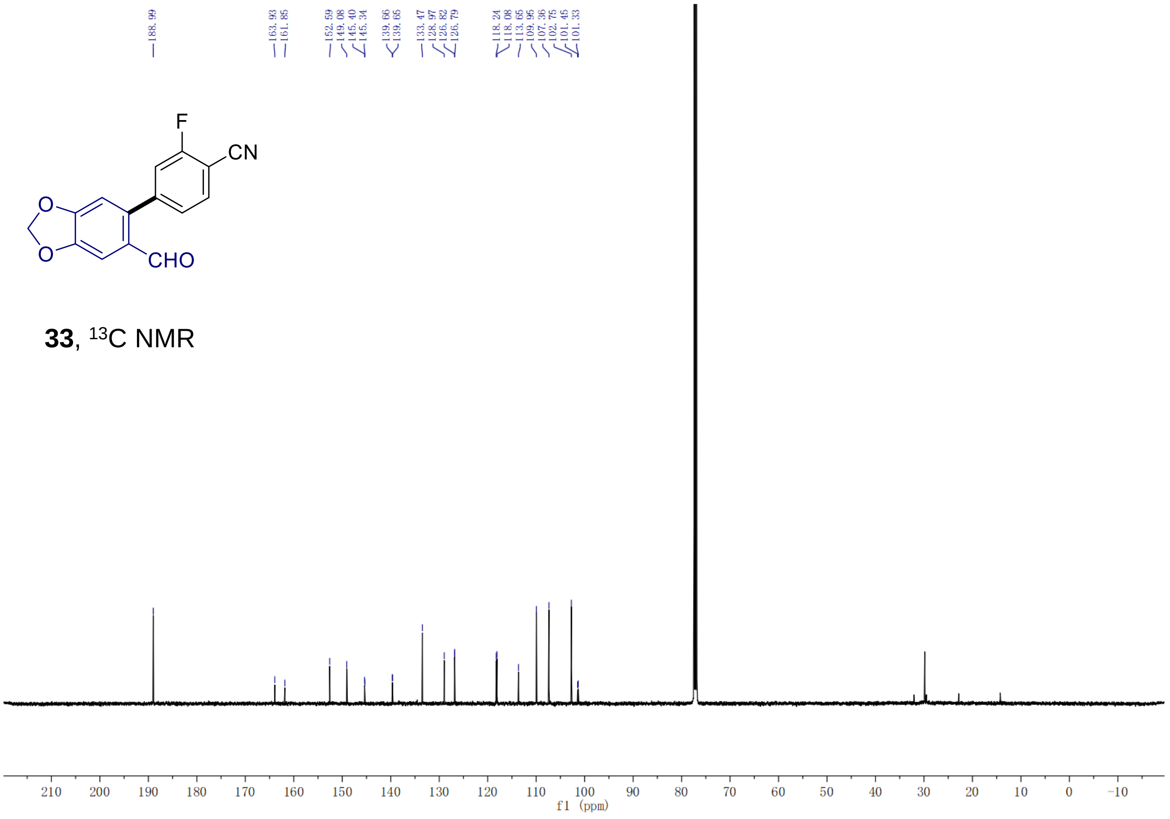

33, ^{13}C NMR

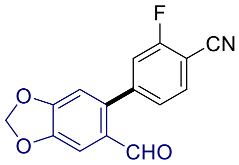
F
CN
O
O
CHO

33, ^{19}F NMR

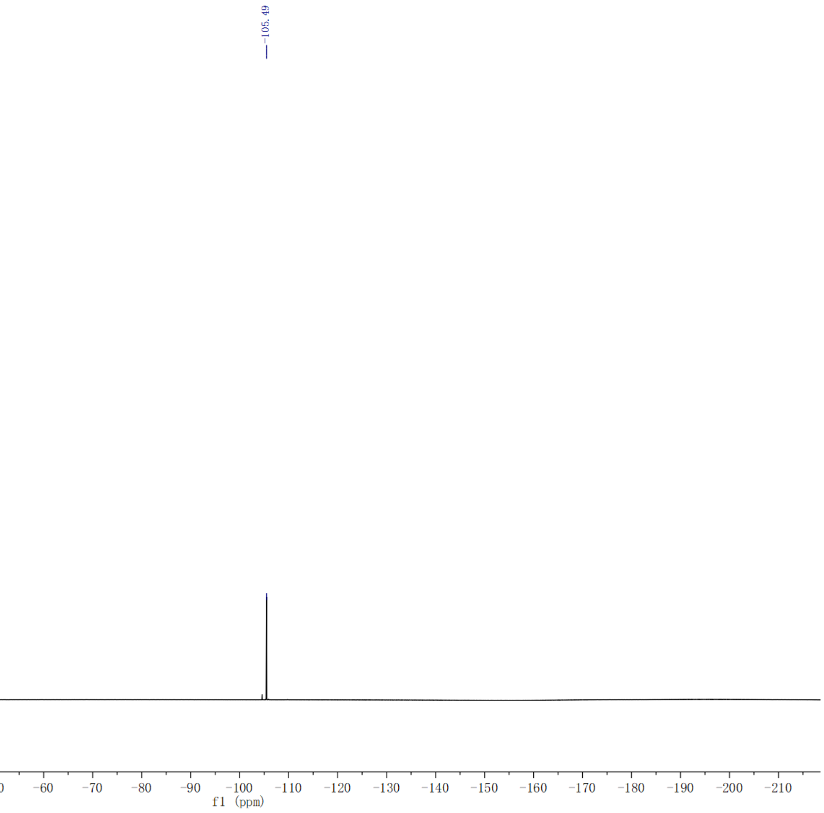
−105.49
10
0
−10
−20
−30
−40
−50
−60
−70
−80
−90
−100
−110
−120
−130
−140
−150
−160
−170
−180
−190
−200
−210
f1 (ppm)

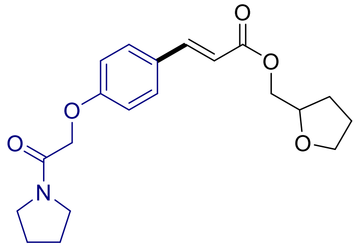

35, ¹H NMR

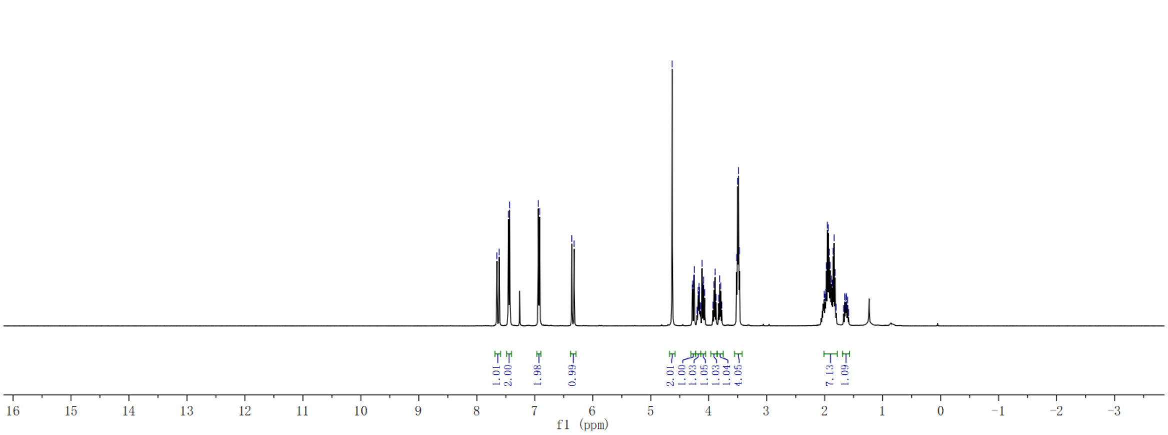

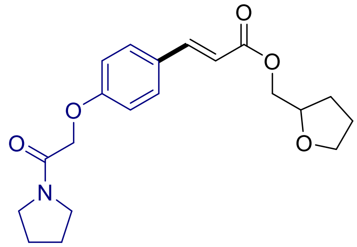

35, ¹³C NMR

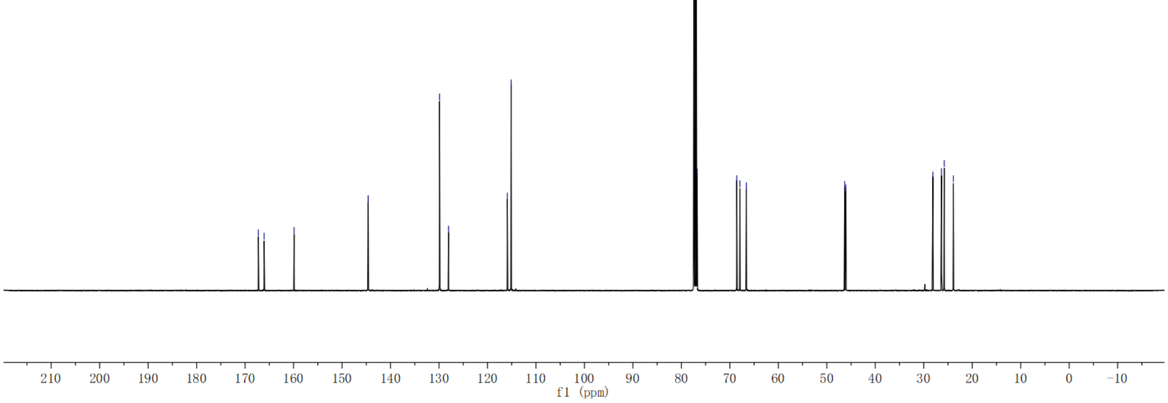

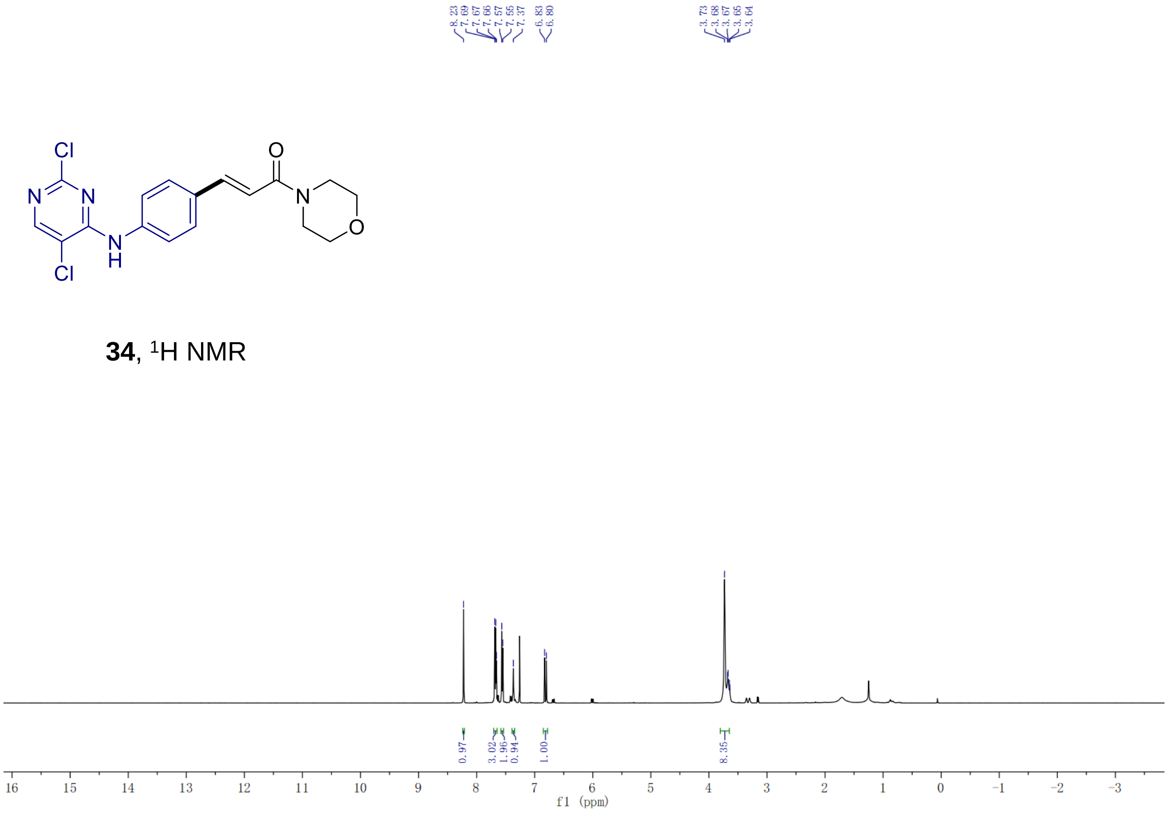

34, ^{1}H NMR

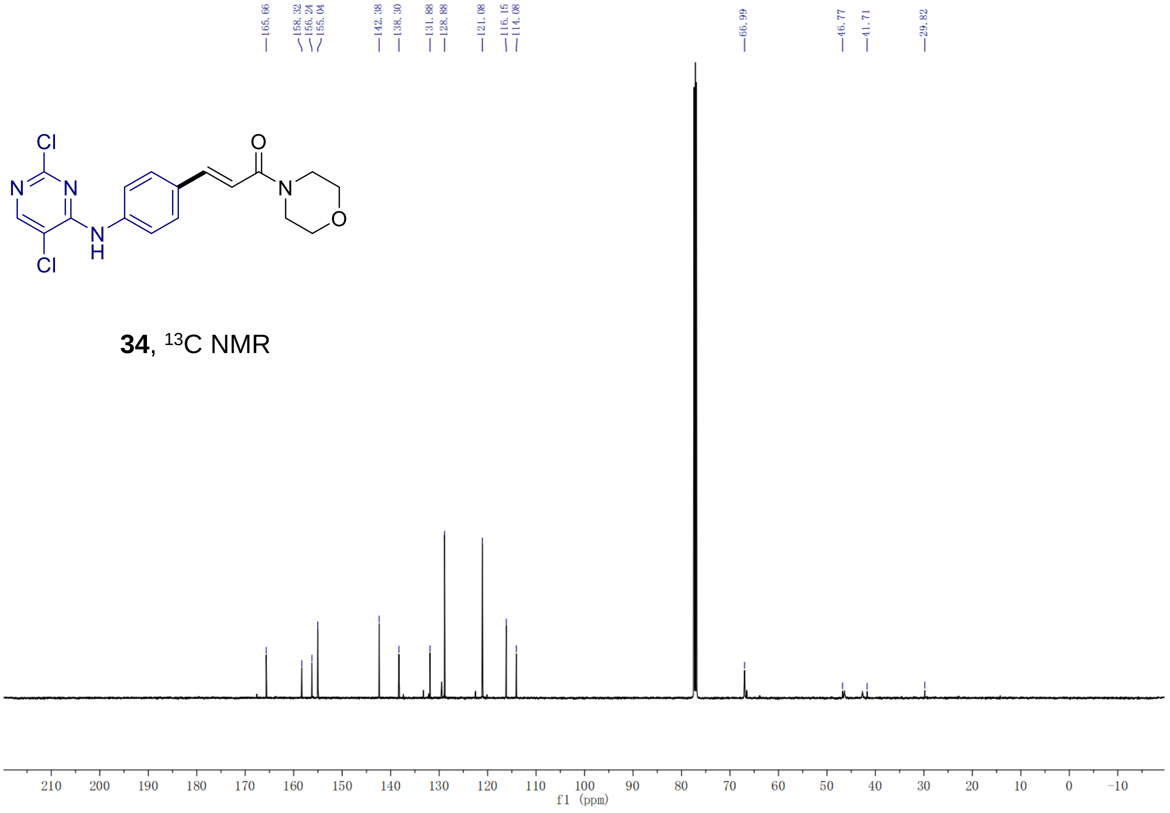

34, ^{13}C NMR

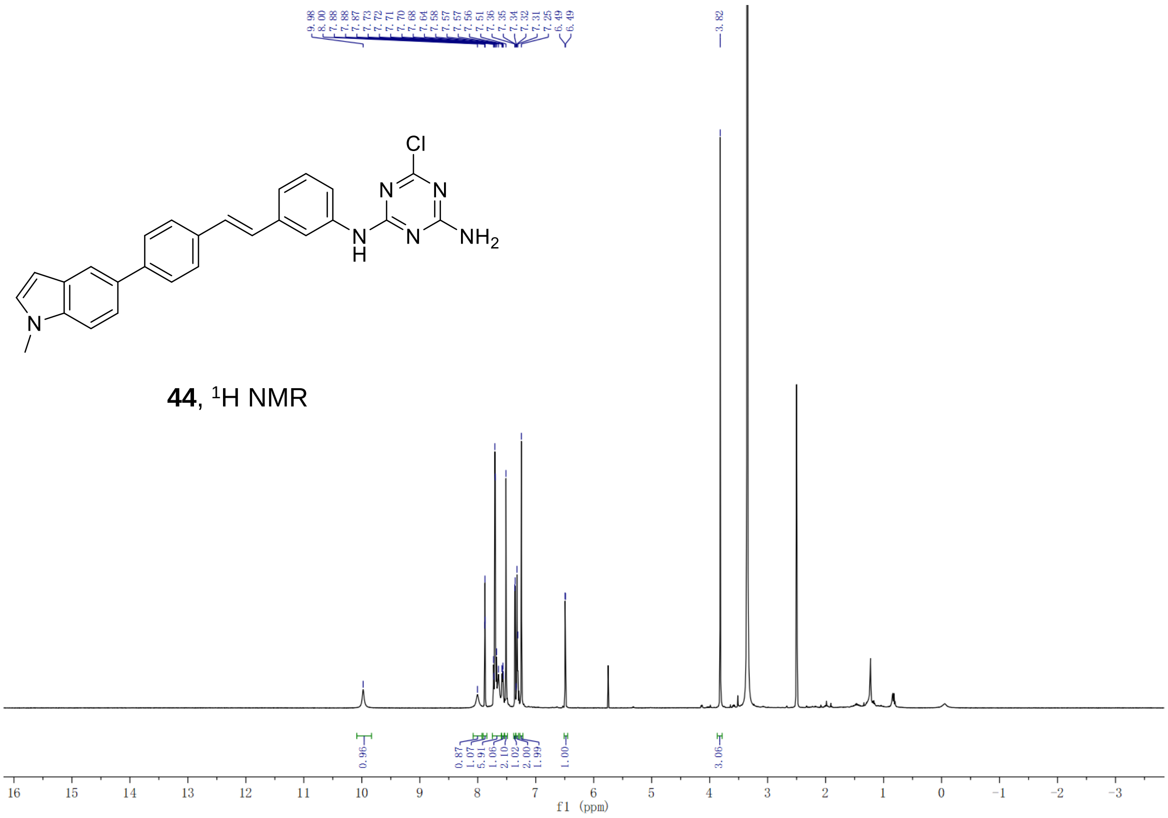

44, ^{1}H NMR

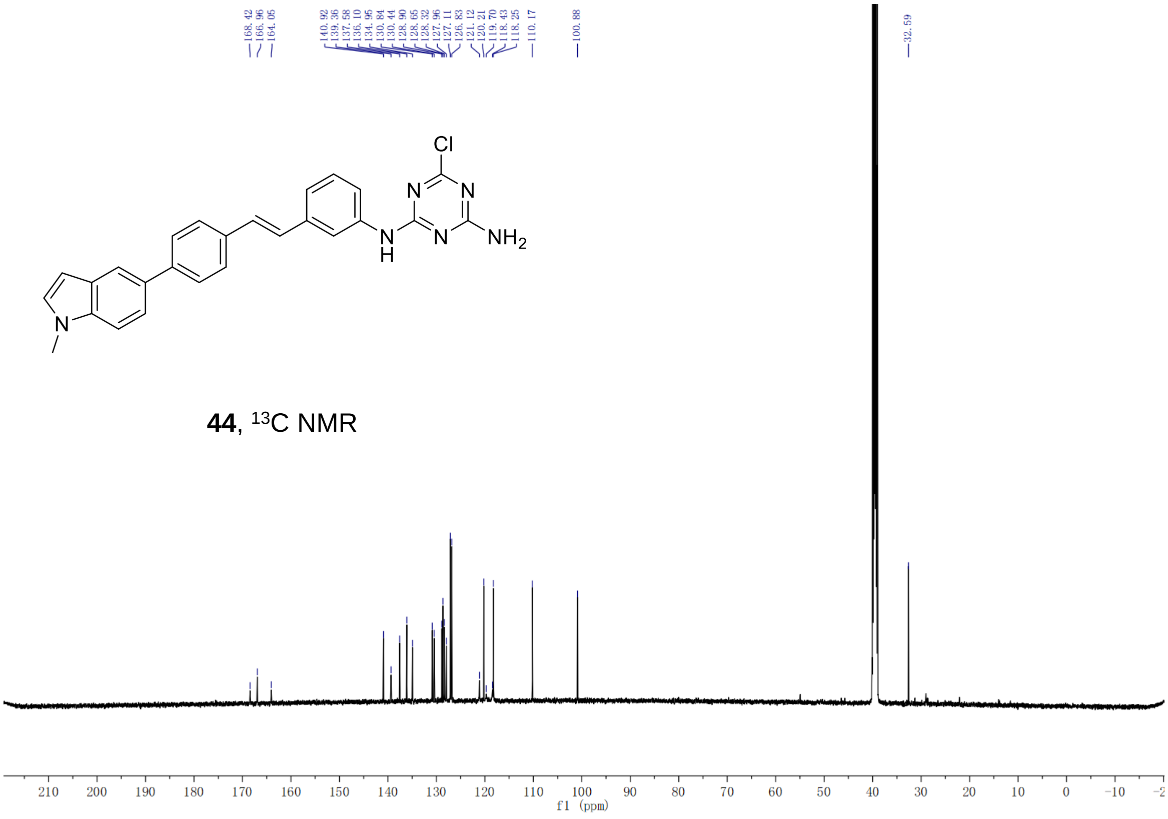

44, ^{13}C NMR
